# Thermally coupled solid hydrogen storage and carbon capture for balancing intermittent renewable energy

**DOI:** 10.1038/s41467-026-72035-1

**Published:** 2026-04-21

**Authors:** Alexander R. P. Harrison, George J. Fulham, Haoliang Hong, Binjian Nie

**Affiliations:** 1 Department of Engineering Science, University of Oxford, Oxford, UK; 2 Department of Chemical Engineering, Imperial College London, London, UK; 3 Department of Chemical Engineering and Biotechnology, University of Cambridge, Cambridge, UK

**Keywords:** Chemical engineering, Carbon capture and storage, Chemical hydrogen storage, Energy modelling

## Abstract

Wind turbines provide renewable power with near-zero CO_2_ emissions, but struggle to achieve steady electricity supply, owing to inherent wind speed variability. Hence, clean energy carriers, such as ‘green’ hydrogen from electrolysis, are required to balance daily power output, and minimise reliance on dispatchable fossil fuels during periods of insufficient wind. Here, we present a system for integrating solid-state hydrogen storage with carbon capture via magnesium looping, using waste heat from the hydrogen storage reaction to drive the process. Incorporating magnesium looping as thermo-chemical energy storage overcomes a major limitation of solid-state hydrogen storage (poor thermal efficiency), and offsets CO_2_ emissions from the use of back-up gas turbine capacity. Thermal integration of the MgH_2_ storage improved round-trip efficiency (conversion from electricity to stored H_2_, and back to electricity) to  ~ 19%, comparable to liquid or gas storage, whereas MgH_2_ alone without heat recovery is limited to  ~ 4%. We model power supply and energy storage over five years for onshore and offshore windfarms using real-world data, finding combined hydrogen storage with magnesium looping is the only system able to meet daily electricity demand and compensate for seasonal wind capacity factor variation, while offsetting CO_2_ operating emissions from flexible gas deployment.

## Abstract (web summary)

This work proposes a system that integrates solid hydrogen storage with carbon capture using magnesium looping. It uses waste heat to improve efficiency, balancing wind energy supply and reducing emissions, potentially achieving near net-zero carbon intensity in electricity generation.

## Introduction

In order to provide a stable electricity supply to users, energy systems based on intermittent renewable power rely on a combination of energy storage, excess generation capacity, and backup fossil fuel infrastructure. Wind energy, generated using onshore and offshore windfarms, forms a key component of global plans for decarbonised power generation^1,2^, both for delivering electricity directly to users via the electricity grid, and for production of power-to-X green fuels (where X = e.g., hydrogen^3,4^, methanol^5^, or ammonia^6^). However, wind power output is inherently intermittent over multiple timescales, from random sub-minute fluctuations as a result of air turbulence^7,8^ and diurnal variation with air and ground temperature^9,10^, to multiple day Dunkelflaute events of anomalously low wind speeds^11^, and seasonal variation arising from long-term weather patterns^12,13^.

**Fig. 1:**
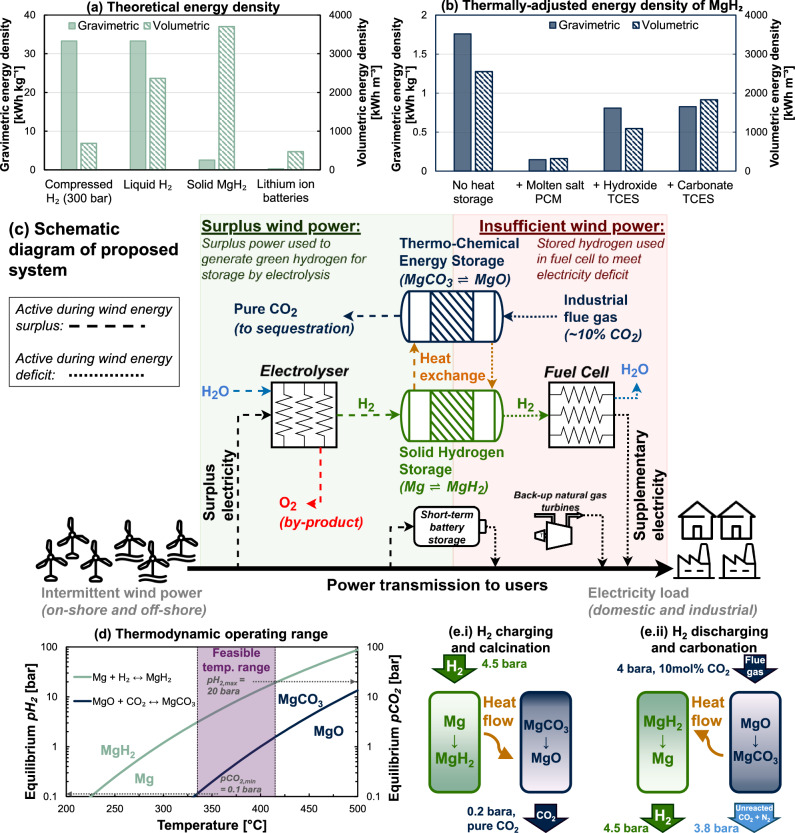
Coupled solid-hydride hydrogen storage and magnesium looping TCES. **a** Comparison of theoretical energy density between gaseous and liquid H_2_, hydrogen stored in magnesium hydride, and lithium ion batteries. **b** Effective energy density of MgH_2_ hydrogen storage combined with energy recovery materials (NaNO_3_ PCM, Mg(OH)_2_-MgO or MgCO_3_-MgO TCES), with the mass of energy recovery material set such that all of the reaction enthalpy of MgH_2_ decomposition to Mg + H_2_ can be recovered. **c** Simplified schematic of the coupled hydrogen storage and carbon capture system modelled in this work, showing streams and unit operations active during a net energy surplus (green pane and dashed lines) or during a net energy deficit (red pane and dotted lines), with a flowchart summarising the modelled system operation given in Supplementary Note 4 and Supplementary Fig. S2. **d** Theoretical equilibrium partial pressures of H_2_ and CO_2_ for the reactions Mg + H_2_*⇌* MgH_2_ and MgO + CO_2_*⇌* MgCO_3_, where the shaded operating region corresponds to the temperature range 335–415 °C, such that *pH*_2_ ≤ 20 bara and *pCO*_2_ ≥ 0.1 bara. If the system were operated at a higher set point temperature (> 415 °C), the equilibrium hydrogen pressure of MgH_2_ would exceed the output pressure of the PEM electrolyser, and would therefore require additional pre-compression of the H_2_ feed. Similarly, if the system were operated at a lower setpoint temperature (< 335 °C), the equilibrium CO_2_ pressure during CO_2_ release from MgCO_3_ calcination would be lower than the partial pressure of CO_2_ in the flue gas feed. **e** Schematic showing heat flow between reactions during hydrogen storage and release, with arrows indicating direction of heat transfer, and gas flows in and out of each reactor. Stream pressures shown correspond to operation at a nominal setpoint of 350 °C, with a pressure swing between carbonation and calcination.

Grid-scale batteries can effectively balance short-term variation in renewable energy supply over the course of a day^14,15^ with a relatively fast response time, but have a maximum feasible discharge time of around 4 h per cell^16^, and high cost per unit of energy stored^17^. Therefore, in order to balance daily and seasonal variation in electricity supply and demand (shown in Supplementary Note 1 and Supplementary Fig. S1), medium- (> 1 week) to long-duration (> 1 month) energy storage is required^12,13,18^, with hydrogen-based energy storage showing greater energy density than battery systems, and lower long-term storage costs^13,19,20^. Of hydrogen storage methods (shown in Fig. 1a), solid hydrogen storage in the form of metal hydrides^21^ offers the highest theoretical volumetric energy density, and improved safety relative to gaseous or liquid storage owing to a reduced risk of H_2_ leakage or boil-off^22,23^.

During periods of strong wind, excess energy can be used in an electrolyser to split water, with the H_2_ reacted reversibly with a metal (M) to form a hydride (MH_2_), releasing heat. Then, during periods of low electricity supply, heat is supplied to thermally decompose the hydride, releasing hydrogen gas for use in a fuel cell or gas turbine^4,24^ to generate electricity. Hydride-based systems for stationary and mobile hydrogen storage have been applied at the pilot scale (100–10,000 kg of hydride)^24,25^, using Mg-based alloys to absorb H_2_ and form MgH_2_ (with up to 7.6 wt% H_2_ capacity in pure MgH_2_).

However, substantial amounts of heat are required to drive the endothermic reaction MgH_2_ → Mg + H_2_ ($\Delta H=+ 10.4{{{\rm{kWh}}}}\,{{{{\rm{kg}}}}}_{{H}_{2}}^{-1}$), wasting > 30% of the lower heating value of the stored hydrogen^4,26^.

Previous research (compiled in Supplementary Note 2 and Supplementary Table S1) has combined hydride-based H_2_ storage with materials for thermal energy recovery to minimise the amount of wasted heat during storage and discharging^22,27^, focussing on latent heat storage using phase change materials (PCMs, e.g., nitrate salts^28,29^, eutectic alloys^24^, or hydrocarbon waxes^30,31^), and thermo-chemical energy storage (TCES) using the reversible dehydration of magnesium hydroxide (Mg(OH)_2_*⇌*MgO + H_2_O, $\Delta H=+ 1.2{{{\rm{kWh}}}}\,{{{{\rm{kg}}}}}_{{H}_{2}O}^{-1}$)^32–34^. However, with both PCMs and hydroxide-based TCES systems, the energy storage material is applied solely for internal heat recovery rather than driving a useful reaction, with a trade-off between the energy efficiency and effective energy density of the system^28,33^, when the mass and volume of both materials are considered (shown in Fig. 1b).

Other studies have also pointed towards the potential for thermal energy export from hydrogen storage^35–37^, but have not considered coupling the hydrogenation reactions with a useful endothermic chemical process, and do not always account for the thermal energy input required to discharge stored hydrogen (discussed in Supplementary Note 2 and Supplementary Table S2). For example, a recent analysis^37^ considered the use of electrolysis and solid-state hydrogen storage for H_2_ production from renewables, where heat evolved during hydrogenation of a Mg-based alloy was integrated with a solid-oxide electrolyser to pre-heat the feed water for high-temperature electrolysis (c. 800 °C), improving overall electrolyser efficiency by c. 10%. However, endothermic hydrogen discharging assumed availability of an abundant, relatively high-grade (≥300 °C) industrial waste heat source, or the use of electrical heating with poor exergy efficiency. Moreover, effects of daily variation in renewable energy supply on system operation were not within the scope of the study, instead using average solar irradiation data to compare the relative levelised cost of H_2_ production and storage between locations, and, as the system was designed to produce H_2_ as a product, temporary fluctuations in output were acceptable, as opposed to meeting a specified electricity demand.

Here, we present a system for combining metal hydride H_2_ storage with carbon capture via magnesium looping (MgL)^38,39^ for supplying stable, low-carbon electricity to users, with a simplified schematic of the process shown in Fig. 1c. By exploiting the overlap in operating temperature between hydride storage and MgL (c. 335–415 °C, shown in Fig. 1d), excess heat from hydrogen storage is used to drive a carbon capture process, which simultaneously acts as thermo-chemical energy storage^40–42^.

On days with a net surplus of wind energy, excess power is used in a low-temperature polymer electrolyte membrane electrolyser (PEM, operating at c. 50 °C, with relatively rapid start-up and shut-down^43^) to generate hydrogen, which is stored in the form of MgH_2_. The heat of reaction is used to calcine MgCO_3_ (MgCO_3_*⇌* MgO + CO_2_, $\Delta H=+ 0.73\,{{{\rm{kWh}}}}\,{{{{\rm{kg}}}}}_{{CO}_{2}}^{-1}$), producing a stream of pure CO_2_ (shown in Fig. 1e.i). For this work, we considered the energy required to compress the CO_2_ generated to 150 bara for pipeline transport^44^, with subsequent undersea sequestration or utilisation in a separate process outside the system boundary.

The purpose of the storage system as designed is to allow for pseudo-continuous power output to users at specified capacity, by using energy storage to balance variation in daily wind power supply. In the event of a net daily shortfall in wind energy, the MgO is carbonated by capturing CO_2_ from industrial flue gas (c. 10vol% CO_2_), generating heat to decompose the MgH_2_ and liberate hydrogen gas (shown in Fig. 1e.ii), which is then converted to electricity in a PEM fuel cell. The hydrogen used here for energy storage remains within the closed system rather than being exported as a product, avoiding the technical and commercial challenges associated with the transport and distribution of hydrogen as a fuel^19,45^.

This study considers how carbonate-based TCES can be applied in solid-state hydrogen storage with simultaneous carbon capture, using real-world wind energy data to evaluate the operation and cost of the overall system. We demonstrate that, in principle, the combined hydride-TCES system can achieve stable electricity generation at a comparable cost to gas turbine power plants, while also capturing CO_2_. By comparing our proposed technology with alternative systems for hydrogen and energy storage, we show that hydride storage with carbonate TCES is the only system able to achieve net-zero emissions. Without using the reaction heat to drive parallel carbon capture, the economic and environmental benefits of hydride storage alone are limited, with the heat integration proposed here overcoming longstanding problems with the technology.

For practical reactor systems incorporating heat recovery from hydrogen storage, the geometric design of the reactor has a marked effect on heat transfer efficiency, with e.g., segmented beds^28^, or optimised reactor aspect ratio^29^ improving performance. However, for the purposes of system analysis, a 0-dimensional thermodynamic model provides useful insights into system behaviour and efficiency, allowing for future optimisation of both reactors, or combining both hydrogen storage and TCES into a single vessel^34^.

## Results

### Experimental measurements of hydrogen sorption and magnesium looping

**Fig. 2:**
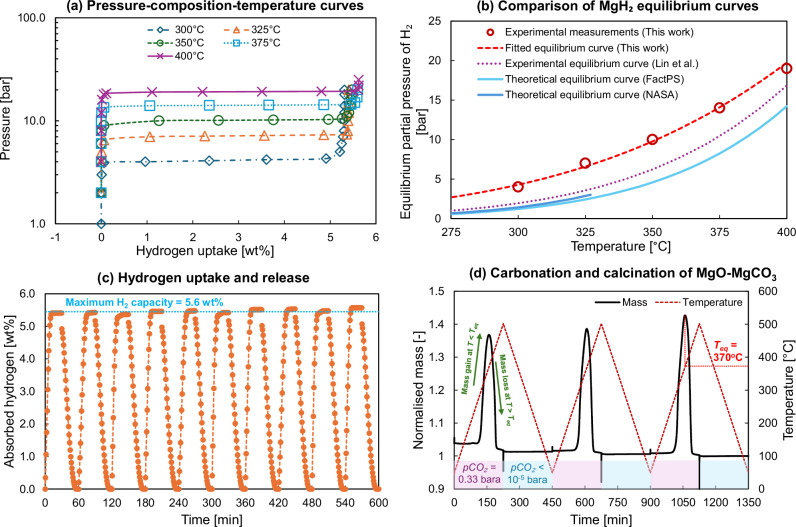
Experimental verification of hydrogen absorption and magnesium looping characteristics. **a** Hydrogen absorption pressure-composition-temperature (PCT) isotherms for the Mg-based hydrogen storage alloy prepared here, **b** fitted equilibrium curve of hydrogen partial pressure against temperature, as compared with values reported by Lin et al.^46^ for an Mg-based alloy material, and values calculated for Mg + H_2_*⇌* MgH_2_ using values from the FactPS^47^ and NASA Glenn coefficient thermodynamic databases^48^ (with the latter only providing data for MgH_2_ up to 327 °C), **c** ten cycles of hydrogenation (pH_2_ = 20 bara) and dehydrogenation in He (20 bara total pressure, pH_2_ = 0 bara) at 350 °C for the Mg-based material prepared, **d** three carbonation-calcination cycles from 50 to 500 °C at a ramp rate of 2 °C min^−1^ for MgO-based material in the thermogravimetric analyser (TGA), normalised with respect to the final mass. Construction lines indicate the estimation of the equilibrium temperature (*T*_*eq*_) at pCO_2_ = 0.33 bara from the maximum mass gain; at temperatures exceeding *T*_*eq*_, the sample loses mass from carbonate decomposition. Irreversible mass loss in the first cycle corresponds to removal of adsorbed impurities from exposure to laboratory air.

Experimental measurements of hydrogen and CO_2_ sorption, shown in Fig. 2, were performed to estimate realistic maximum capacities of Mg and MgO-based sorbent materials, respectively, and to validate the estimated material properties from thermodynamic calculations. Hydrogen absorption isotherms over the temperature range 300–400 °C are shown in Fig. 2a. The maximum hydrogen uptake by the Mg-alloy for measurements at ≥320 °C was 5.6 wt%, below the theoretical value of 5.9 wt% for an alloy composed of 76.5 wt% Mg. Furthermore, the equilibrium partial pressures estimated from experiments (shown in Fig. 2b) exceeded previous values for hydrogenation of MgH_2_-based material reported by Lin et al.^46^, and values calculated for reaction of pure Mg to MgH_2_ from databases of material thermodynamic properties (FactPS^47^, and NASA Glenn coefficients^48^). Therefore, the maximum hydrogen capacity of the Mg-based alloy used in calculations was set at 5.6 wt% for the base-case scenario as a conservative estimate. For all temperatures investigated, ${p}_{{H}_{2}}$ at equilibrium was < 20 bar, allowing for hydrogen to be delivered directly to the storage reactor from the PEM electrolyser (operating at 20 bara) without pre-compression. Figure 2c shows ten cycles of hydrogen uptake at pH_2_ = 20 bara and release for the Mg-based alloy at 350 °C in a batch reactor, with the material absorbing 5.6 ± 0.1 wt% H_2_, with no reduction in H_2_ capacity over 10 repeated cycles. Helium gas was used to lower the pH_2_ during desorption by displacing hydrogen gas; in a real hydrogen storage process, a temperature swing would be applied to induce desorption to avoid diluting the hydrogen produced^46^.

Figure 2d shows the carbonation of the MgO-based material during heating in CO_2_. At a CO_2_ partial pressure of 0.33 bara, the sample started to carbonate at around 300 °C, gaining 39 ± 3 wt% CO_2_, averaged over three cycles. Once the equilibrium temperature at *pCO*_2_ = 0.33 bara was exceeded, the sample then began to decarbonate at 370 ± 1 °C, slightly higher than the theoretical equilibrium temperature of 365 °C. The theoretical maximum CO_2_ uptake for complete conversion of MgO → MgCO_3_ is 87 wt% (determined from reaction stoichiometry); however, in practice sorbent rarely reach the theoretical maximum as a result of formation of an impermeable carbonate shell around the particles from volume expansion during carbonation, inhibiting inward CO_2_ transport^49^. Nevertheless, the measured CO_2_ capacity of 39 wt% was similar to reported experimental values^40,50^ of c. 40 wt%. Therefore, for the pessimistic case a CO_2_ capacity of 35 wt% was assumed, for the base-case, the reported value from literature of around 40 wt% was used^40^, and for the optimistic case, a higher value of 50 wt% was used^50^, allowing for improvements in sorbent design and composition to mitigate mass transfer limitation.

### System design and round-trip efficiency

**Fig. 3:**
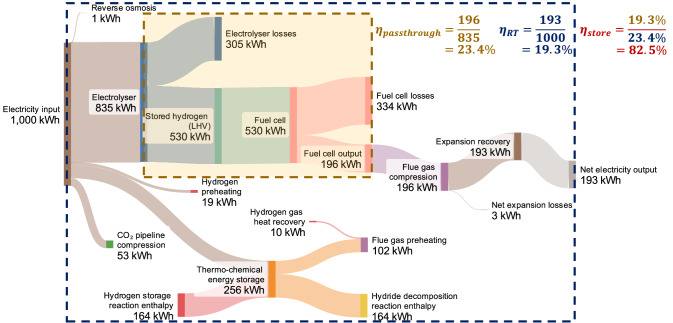
Energy conversions during hydrogen storage and release. Simplified Sankey diagram for the conversion of 1000 kWh of input electricity to hydrogen (c. $16\,{{{{\rm{kg}}}}}_{{H}_{2}}$), and subsequent conversion back to electricity with values given under base-case modelling assumptions, with an overall round-trip electrical efficiency of around 0.19. Heat evolved by the hydrogen storage reaction was assumed to be stored in the TCES material by calcination of MgCO_3_ at a nominal setpoint of 350 °C, releasing 353 kg of CO_2_ for subsequent compression and pipeline transportation. The TCES material was later discharged by carbonation of the MgO with an equivalent mass of CO_2_ from compressed flue gas, in order to generate heat to liberate the stored hydrogen and pre-heat the flue gas feed. Note that the steps do not necessarily occur chronologically in the order shown, as e.g., power from the wind farm is used to compress flue gas during fuel cell start-up. For transmission purposes, electricity generated from the windfarm and the fuel cell were considered to be equivalent, neglecting any energy losses during voltage conversion. Ambient heat losses from natural convection were estimated at a constant value of 32 kWh m^−1^d^−1^ based on the external dimensions and operating temperatures of the reaction vessels, corresponding to around 2–4% of total lost energy depending on the system configuration. Dashed boxes indicate the system boundaries used to calculate round-trip efficiency, *η*_*RT*_ (Eq. (5)), and pass-through efficiency, *η*_*passthrough*_ (Eq. (6)), with the storage efficiency defined as the ratio *η*_*RT*_/*η*_*passthrough*_ (Eq. (7)).

For the storage system shown in Fig. 1c, the overall efficiency is determined by the electrical efficiency of the electrolyser and fuel cell for converting electricity into hydrogen and vice versa, the reaction enthalpies of the hydrogenation and carbonation reactions, and the amount of energy required to compress the feed gases to reactor pressure. The main energy conversions when electricity is converted to hydrogen, and back to electricity when needed, are shown in the Sankey diagram in Fig. 3, estimated using base-case modelling assumptions (summarised in Supplementary Note 4, Supplementary Figs. S5 and S4, Supplementary Note 5, and Supplementary Table S5), with assumed PEM electrolyser and fuel cell efficiencies of $0.019\,{{{{\rm{kg}}}}}_{{H}_{2}}\,{{{{\rm{kWh}}}}}^{-1}$ and $12.3\,{{{\rm{kWh}}}}\,{{{{\rm{kg}}}}}_{{H}_{2}}^{-1}$ (37% ${{{{\rm{LHV}}}}}_{{H}_{2}}$) respectively, based on reported values from literature^51–53^. As shown in Supplementary Note 4 and Supplementary Fig. S4, a reactor operating temperature of 350 °C provided the optimal trade-off in thermodynamic efficiency for both reactions, giving the highest overall round-trip efficiency of conditions investigated. The reactors were assumed to be insulated and nominally isothermal, with additional heat supplied to compensate for ambient heat losses by electrical heating, as shown in the process flow diagram, Supplementary Fig. S3.

During hydrogen conversion to electricity for the system as configured here, the net electricity generated in the fuel cell is used to compress the flue gas feed to the TCES reactor, in order to achieve sufficient *pCO*_2_ to drive the exothermic carbonation reaction. The pressurised gas leaving the reactor (N_2_ and unreacted CO_2_) was then expanded to ambient pressure, generating electricity for delivery to users. As described in Supplementary Note S4.6, the gas leaving the reactor is at a higher temperature (here, 350 °C) than the flue gas compressor outlet (200 °C), allowing some conversion of heat to work via the Brayton cycle, and therefore giving relatively low net energy losses (here, 3 kWh) as a result of compression and expansion operations, despite irreversible losses in turbomachinery and consumption of gas in the carbonation reaction. Mass flow rates of process streams are given in Supplementary Note 6 and Supplementary Table S7.

Accounting for the additional work required to store and release hydrogen, but neglecting ambient heat losses, *η*_*RT*_ was around 0.19, i.e., corresponding to a storage efficiency of *η*_*store*_ = 0.825. From the definition of the passthrough efficiency, the maximum theoretical round-trip efficiency is given by *η*_*RT*_ = *η*_*passthrough*_ = 23.4%, with full energy recovery from hydrogen storage. Hence, the storage system achieved 82.5% of the maximum theoretical efficiency. Maximum and minimum round-trip efficiencies of *η*_*RT*,*opt*_ = 0.22 and *η*_*RT*,*pess*_ = 0.09 were estimated under optimistic and pessimistic modelling assumptions, respectively, based on the range of model input parameters reported in Supplementary Table S5.

For a solid MgH_2_-based hydrogen storage system with no heat storage, the approximate round-trip efficiency was given by Eq. (1), assuming, for fair comparison, that electrical heating was used to maintain isothermal reactor operation (with further commentary on alternative heating configurations using hydrogen combustion in Supplementary Note 4).

1 $${\eta }_{RT,noheatstorage}\,\approx \,{\xi }_{elec}\cdot \left({\xi }_{fuellcell}\,-\,\Delta {H}_{{H}_{2}}\right)=0.04$$

and the corresponding storage efficiency was *η*_*store*_ = 0.16. Therefore, around 84% of theoretically available energy was lost relative to the pass-through efficiency, and hence, incorporating heat storage using magnesium carbonate improved net round-trip efficiency approximately fivefold.

Possibly counter-intuitively, the round-trip efficiency of the system was maximised by allocating a fraction of the input electricity to calcine the TCES material via electrical heating (here, 92 kWh), rather than directing all of the energy to electrolysis. Storing some of the input energy as heat rather than hydrogen allowed for some process heating requirements to be shifted from the energy-deficient discharging step to the energy-abundant charging step, improving overall *η*_*RT*_ from 0.15 to 0.19 (shown in Supplementary Note 4). If all the available surplus power were directed towards electrolysis, a greater mass of hydrogen would be stored; however, as a result of energy losses in the electrolyser, less heat would be stored in the TCES material. Hence, during discharging, insufficient heat would be available from the TCES material to fully discharge the stored hydrogen, requiring the use of additional electrical heating at a point in the cycle where less energy is available in total (as the hydrogen is discharged during periods of net electricity deficit).

The effects of altering other process parameters (reactor temperature, driving force for heat transfer, CO_2_ feed concentration, and CO_2_ capture efficiency) are discussed in Supplementary Note 4 and Supplementary Fig. S4.

### Modelled operation with windfarm data

**Fig. 4:**
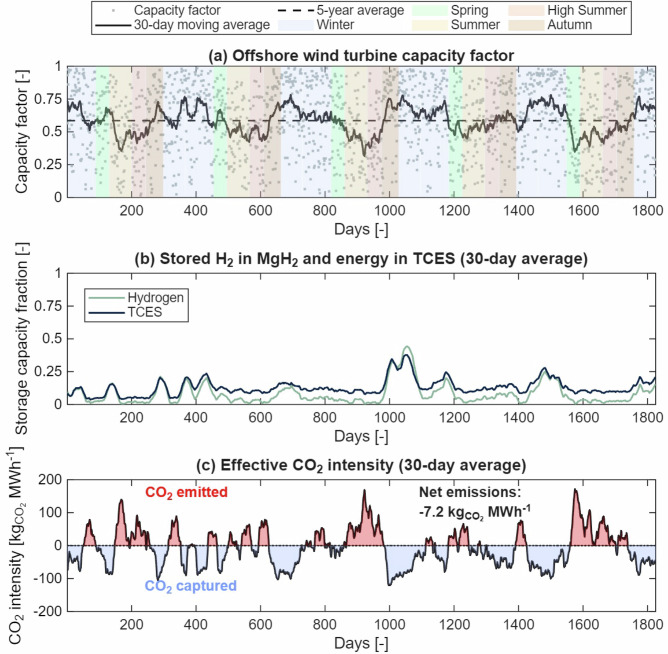
Modelling five years of intermittent wind power with hydrogen storage and carbon capture. Modelling results under base-case assumptions for 5 years of operation of an offshore windfarm located in the North Sea, with 25% net overcapacity relative to average demand (nameplate capacity: 42,700 kWh d^−1^), and H_2_ storage capacity equivalent to 6 days of energy demand (167 tonnes of Mg alloy; 478 tonnes of MgCO_3_): **a** Variation in windfarm capacity factor used to estimate power input, **b** Variation in stored H_2_ in MgH_2_, and stored heat in TCES material (MgCO_3_ ↔ MgO), as a fraction of total capacity, **c** Estimated carbon intensity of power generation and net carbon intensity over full modelling period. Corresponding results for onshore wind are presented in Supplementary Note 6 and Supplementary Fig. S7.

The storage system with carbonate TCES was then modelled using input wind power data for the period 01/01/2016-31/12/2020, for a simulated offshore windfarm in the North Sea (shown in Fig. 4a, with further system parameters given in Supplementary Note 6 and Supplementary Table S8). Nominal windfarm capacity was scaled using Eq. (2) (as described in the “Methods” section) according to the average wind capacity factor over the five-year period^5^, and an additional overcapacity factor (*f*_*OCP*_) included such that the windfarm provided a net surplus of energy with respect to consumption, calculated based on five-year time-averaged demand. The hydrogen storage capacity was set arbitrarily such that total storage was equivalent to 6 days of energy demand.

Over each year, daily power generation in autumn and early winter exceeded demand, allowing excess electricity to be stored as hydrogen (with storage levels shown in Fig. 4b). The stored hydrogen was then consumed to compensate for deficits in energy generation, albeit with considerable inter-annual variation in peak stored energy. The effective carbon intensity of the generated electricity (defined as the net tailpipe CO_2_ operating emissions as a result of combined-cycle gas turbine (CCGT) utilisation and CO_2_ capture from the TCES cycle) is shown in Fig. 4c, with net CO_2_ capture during H_2_ storage (corresponding to the shaded blue regions), and net CO_2_ emissions as a result of electricity generation from backup gas turbines during periods of prolonged electricity deficit (corresponding to the shaded red regions).

Across the five-year modelling period, the system achieved an overall CO_2_ intensity of −7.2 ${{{{\rm{kg}}}}}_{{CO}_{2}}$ per megawatt-hour of electricity supplied to users (i.e., near net-zero emissions), within the operating boundaries of the process in Fig. 1c. Similar trends were observed for onshore wind (shown in Supplementary Note S6 and Supplementary Fig. S7), albeit with lower average capacity factor and greater variability, resulting greater reliance on back-up gas and hence positive CO_2_ emissions (7.5 $k{g}_{{CO}_{2}}\,{{{{\rm{MWh}}}}}^{-1}$).

### Balancing cost and carbon emissions

**Fig. 5:**
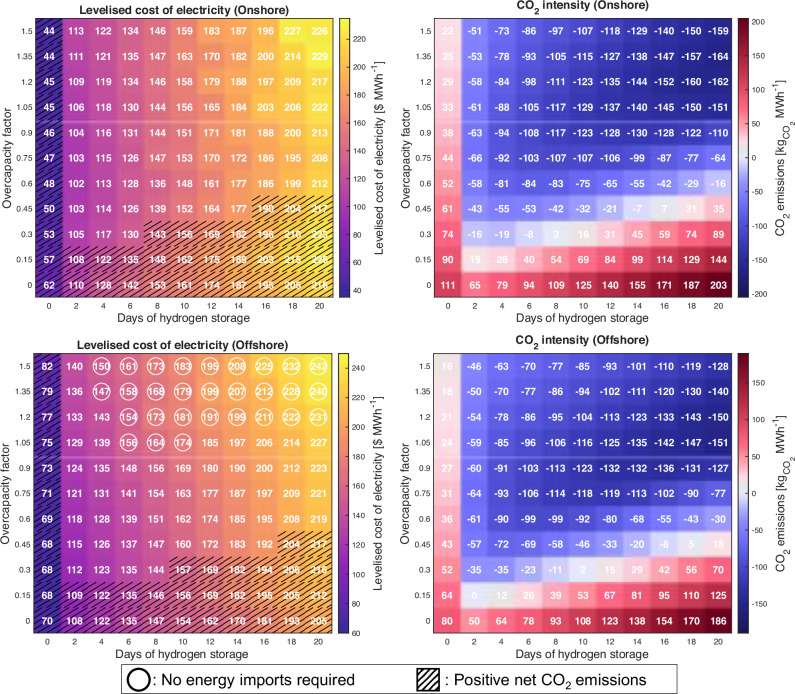
Optimising electricity cost and carbon drawdown. Estimated levelised cost of electricity ($MWh^−1^; yellow = higher cost, purple = lower cost), and carbon intensity of generated electricity ($${{{{\rm{kg}}}}}_{{CO}_{2}}\,{{{{\rm{MWh}}}}}^{-1}$$; red = net-positive CO_2_ emissions, blue = net-negative CO_2_ emissions), for different combinations of excess wind overcapacity, storage capacity of solid H_2_, and windfarm location (offshore or onshore). Estimated carbon intensities correspond to net emissions from backup gas turbine power generation minus CO_2_ drawdown from the MgL cycle. Circled cells correspond to system configurations not requiring any imported electricity from backup gas turbines; hatched cells correspond to configurations unable to achieve net-zero CO_2_ emissions over the modelled 5-year period.

To identify the effects of altering the system design parameters, we repeated the simulations for 121 combinations with 0–20 days worth of hydrogen storage capacity, and scaling the installed wind farm and process equipment according to overcapacity factors from 0 to 1.5 (where a factor of 0 means installing the wind farm capacity to exactly match the time-averaged electricity demand). The capital and operating costs of each system were estimated as described in Supplementary Note 5 and Supplementary Table S5, in order to generate a point estimate of overall levelised cost of electricity (LCOE) for each configuration under base-case modelling assumptions. A summary of the LCOE and average CO_2_ intensity for each configuration under base-case assumptions is given in Fig. 5. For each case, equipment sizing (e.g., electrolyser and fuel cell capacity) were influenced by both overcapacity factor and hydrogen storage capacity, as both parameters affected the maximum available surplus energy that could be used in the electrolyser to produce hydrogen for storage, and, the maximum deficit between windfarm power output and user demand, determining the maximum required conversion of hydrogen into electricity in the fuel cell. Offshore locations were able to achieve lower emissions and a broader region of configurations that did not require external energy imports, albeit with higher LCOE values, in agreement with previous studies of fuel production from wind power^5^. Configurations with very high H_2_ storage capacity and low windfarm capacity (i.e., in the bottom right-hand corners) showed increased LCOE, as a result of increased expenditure on fuel and carbon tax for gas turbine operation, and increased ambient heat losses from large reactor vessels decreasing efficiency.

We also investigated the sensitivity of the estimated values of LCOE and CO_2_ intensity to the simulated windfarm location (for 8 alternative locations in Northern Europe with significant wind energy penetration) and wind speed input data, with results given in Supplementary Note 7.

**Fig. 6:**
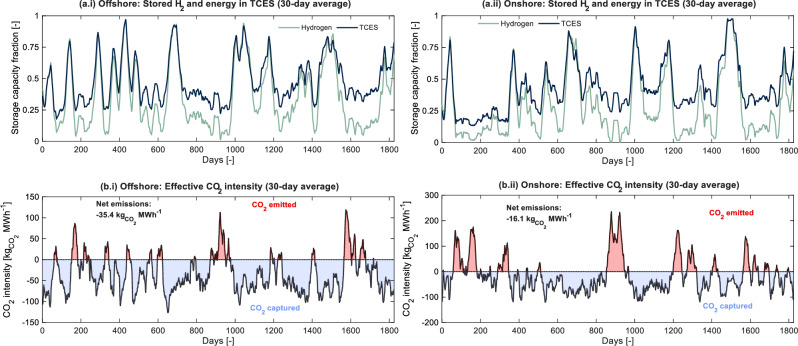
Stored energy and carbon drawdown for the sized system. **a** Modelled H_2_ storage and heat storage in TCES, and **b** effective CO_2_ intensity for (i) offshore and (ii) onshore systems, both with *f*_*OCP*_ = 0.3 and 2 days of H_2_ storage, giving the lowest cost systems with net-negative CO_2_ emissions under base-case assumptions. In both cases, the maximum fraction of H_2_ storage used exceeded the values for the system shown in Fig. 4 (with lower windfarm capacity and greater hydrogen capacity). The additional windfarm overcapacity decreased the system reliance on backup natural gas, and hence decreased net operating emissions of CO_2_.

Given the relatively high cost of the Mg-based alloy for hydrogen storage, LCOE increased approximately linearly with H_2_ storage capacity, and systems with zero days of storage achieved the lowest average LCOE. However, as systems without hydrogen storage relied solely on external energy imports to manage wind power intermittency and did not include any carbon capture capacity, net CO_2_ emissions were positive regardless of the overcapacity factor used. Similarly, for systems with an overcapacity factor of zero (i.e., exactly enough nameplate generation capacity to meet average demand over five years), emissions were positive for all storage capacities, as some excess power was required to counteract the energy losses during H_2_ storage and release, shown in Fig. 3. Therefore, to achieve net-zero CO_2_ emissions for offshore and onshore wind, at least 2 days of hydrogen storage capacity was required, with *f*_*OCP*_ ≥ 0.3, with hydrogen storage and net CO_2_ emissions shown in Fig. 6.

Higher storage capacities or overcapacity factors allowed for greater CO_2_ drawdown, at the penalty of increased LCOE. With *f*_*OCP*_ ≥ 1.05 and at least 6 days of stored hydrogen capacity, the offshore system was able to achieve five years of consistent electricity supply without any external energy imports from the grid, and therefore no CO_2_ emitted from use of backup gas turbines (with the relative reliance on external imports for configurations with lower *f*_*OCP*_ values shown in Supplementary Note S6 and Supplementary Fig. S8). For very large windfarms, carbon drawdown slightly decreased, as the excess capacity of the larger windfarm rapidly filled the H_2_ storage system and became curtailed, therefore decreasing the amount of CO_2_ captured by the MgL TCES subsystem during H_2_ absorption and release.

**Fig. 7:**
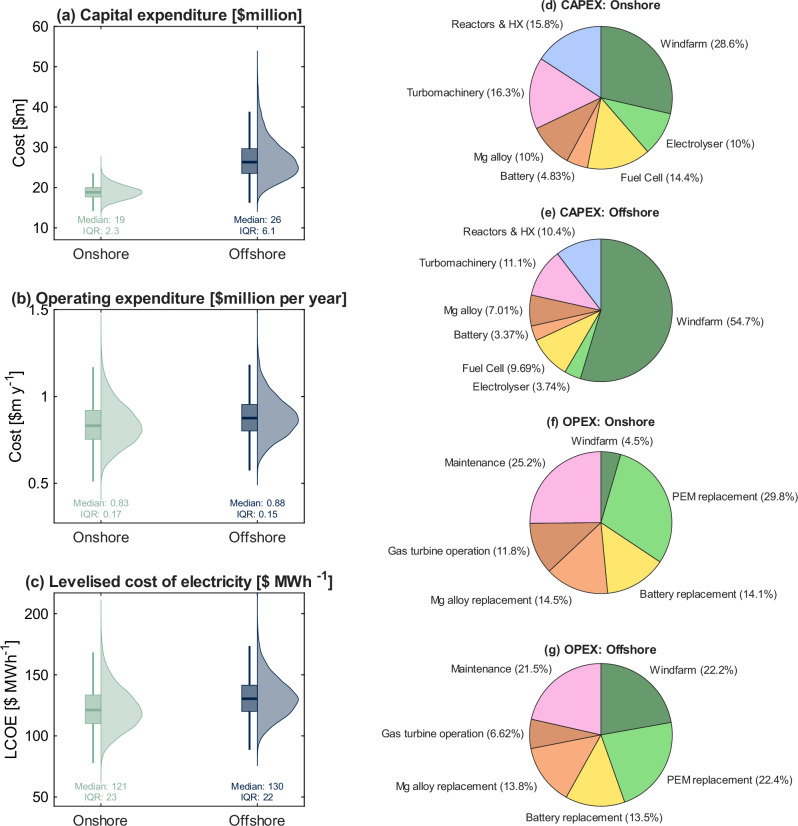
Breakdowns of capital and operating costs. Distributions of estimated (**a**) capital expenditure (CAPEX), **b** operational expenditure (OPEX) and **c** levelised cost of electricity (LCOE) for onshore and offshore systems, both with *f*_*OCP*_ = 0.3 and 2 days of H_2_ storage. Boxes show the interquartile range (IQR) with the median indicated by a horizontal line; whiskers extend to 1.5 × the IQR. Violin widths represent the kernel density estimate (10,000 model iterations). Pie charts (**d**–**g**) show averaged distributions of CAPEX and OPEX for system costs corresponding to at least 1% of CAPEX or OPEX.

We then applied Monte Carlo simulation to quantify the uncertainty in the expected capital and operating expenditure (CAPEX and OPEX) and LCOE, by varying the equipment input parameters (e.g., cost, lifetime, and efficiency) over the ranges reported in literature (Table 1 and Supplementary Table S5, with a full description given in Supplementary Note S5), with cost distributions shown in Fig. 7a, b for the case with *f*_*OCP*_ = 0.3 and 2 days of H_2_ storage, including the cost of a constructing a new-build windfarm of appropriate capacity. While the hydrogen storage system proposed here could also be readily retro-fitted to existing renewable energy infrastructure, thereby decreasing upfront CAPEX, overall LCOE would still depend on the depreciation and financing of the windfarm(s) used.

**Table 1:** Parameters used for estimation of capital and operating costs for each scenario, with corresponding literature source(s)

Item	Unit	Base	Pess.	Opt.	Ref(s)	Ref. years(s)
Fuel cell capital cost	$ $${{{{\rm{kW}}}}}_{installed}^{-1}$$	2687	4000	1620	^86–88^	2015–2025
Electrolyser capital cost	$ $${{{{\rm{kW}}}}}_{installed}^{-1}$$	931	2000	400	^89–94^	2022–2024
Fuel cell/electrolyser lifetimes	years	8.5	4.5	14	^95–100^	2015–2024
Battery capital cost	$ $${{{{\rm{kWh}}}}}_{capacity}^{-1}$$	281	405	134	^17,101–105^	2021–2023
Onshore wind capital cost	$ $${{{{\rm{kW}}}}}_{nameplate}^{-1}$$	1680	2800	1050	^106–111^	2021–2024
Offshore wind capital cost	$ $${{{{\rm{kW}}}}}_{nameplate}^{-1}$$	3785	6500	1839	^107,110–115^	2020–2025
Hydrogen storage alloy cost	$ kg^−1^	16	8	60	^37,116^	2024
Hydrogen storage alloy lifetime	y	16	8	25	^25^	2023

Reference years correspond to the range of publication dates of the cited literature sources.

As well as being dependent on the system design parameters *f*_*OCP*_ and storage capacity as shown in Fig. 5, CAPEX, OPEX, and LCOE were also influenced by assumed equipment input parameters, with overall system cost showing a non-linear relationship with respect to e.g., assumed PEM stack lifetime and Mg alloy lifetime. Therefore, median estimated LCOE from Monte Carlo simulation, as shown in Fig. 7c, exceeded the point-estimates generated from using base-case input values. Hence, a majority of simulated cases cost more than would be expected from a simple point estimate using base-case inputs only, and realistic cost estimates accounting for system uncertainty should err towards higher expected costs to account for uncertainty in equipment costs and lifetimes.

The distributions of capital and operating costs averaged over the simulated cases are shown in Figs. 7d–g. In most cases, the windfarm was the most expensive single item of capital expenditure, comprising 29–55% of total CAPEX, followed by the turbomachinery required for flue gas compression and expansion, the reactor vessels and heat exchange equipment, and PEM equipment for H_2_ conversion. The PEM stacks in the fuel cell and electrolyser, the battery subsystems used to smooth daily power variation, and the Mg-alloy used for H_2_ storage, each comprised a significant fraction of total OPEX, given their relatively high costs, and limited operational lifespans necessitating frequent replacement and maintenance. Furthermore, for the offshore system, the broad range of estimated capital costs (with approximately three times greater variation in CAPEX than the onshore system) was primarily as a result of high uncertainty in offshore windfarm construction and connection costs, as limited remaining availability of unused prime North Sea windfarm locations^54^ results in much higher connection costs for locations ≥50 km from the coast^5,55^.

### Comparison with other energy storage technologies

We then applied the process model to compare the system against the alternative energy storage methods as shown in Fig. 1a, b, namely solid MgH_2_ hydrogen storage without any heat storage, or with MgO-Mg(OH)_2_ hydroxide TCES, liquefied or compressed H_2_, and Li-ion batteries. For all cases, windfarm overcapacity was fixed at 30% (*f*_*OCP*_ = 0.3), and storage capacity was sized to equal 2 days of electricity demand. As a basis for comparison, the cost and carbon intensity of electricity generated from wind power and natural gas (NG) without any form of electricity storage were also calculated. A summary of the process models used for the alternative systems is given in Supplementary Note 8 and Supplementary Figs. S18 and S19.

**Fig. 8:**
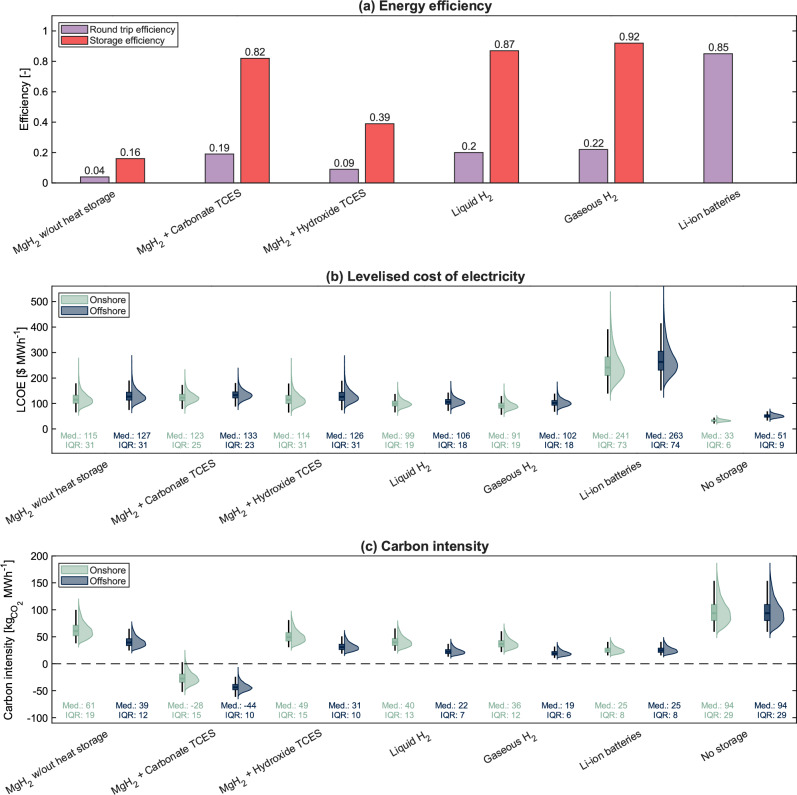
Comparison with other energy storage technologies. Comparison of (**a**) round-trip energy efficiency (with the value for Li-ion batteries estimated from literature^56^) and storage efficiency as defined in Eqs. (5) and (7). **b** levelised cost of electricity and **c** carbon intensity of generated electricity for alternative system configurations. Cost and carbon intensity were estimated by Monte Carlo simulation for systems with *f*_*OCP*_ = 0.3 and 2 days of hydrogen or battery storage capacity as described in Supplementary Note S5. Boxes in (**b**) and (**c**) show the interquartile range (IQR) with the median (Med.) indicated by a horizontal line; whiskers extend to 1.5 × the IQR. Violin widths in (**b**) and (**c**) represent the kernel density estimate (5000 model iterations). In order to maintain a fair comparison between cases, electrical heating was assumed to be used for all MgH_2_-based systems; alternative cases using different heating methods are discussed in Supplementary Notes 4 and 8.

In terms of round-trip energy efficiency and storage efficiency (shown in Fig. 8a), solid H_2_ storage with carbonate TCES outperformed hydroxide TCES (as the heat required to raise superheated steam during MgO hydration decreased overall efficiency to around 0.09), and showed comparable storage efficiency with liquid or gaseous H_2_ storage, overcoming a major shortcoming of hydride-based systems. However, all H_2_-based systems showed inferior round-trip efficiency to Li-ion batteries, reported at c. 85%^56^, primarily as a result of energy losses in the PEM electrolyser and fuel cell. However, when comparing LCOE values (shown in Fig. 8b), the cost of using battery storage only was more than double that of systems using H_2_ (all of which were within c. 10% of one another).

Given the high capital and operating costs of electricity storage, using wind power in combination with gas turbines with no storage was able to provide electricity at significantly lower LCOE (< 50%), albeit with considerably greater carbon emissions as a result of increased natural gas use to cover all deficits in windfarm power supply (shown in Fig. 8c). For all cases using wind power, carbon emissions from offshore wind were somewhat lower than from onshore, as offshore wind showed less variation in capacity factor (and hence, less reliance on external grid imports) for the dataset used.

Unsurprisingly, solid H_2_ storage with incorporated carbonate TCES was the only system able to achieve an average carbon intensity less than zero, by virtue of producing concentrated, high-pressure CO_2_ for sequestration as a by-product of the TCES reaction. Without carbonate TCES, the low round-trip efficiency of MgH_2_ hydrogen storage resulted in higher average carbon intensity than liquid or gaseous H_2_ storage, or Li-ion batteries, and showed only a small improvement in emissions as compared to electricity generation using wind and natural gas with no energy storage at all.

Estimated LCOE for the relatively simple, self-contained, system considered here (121 ± 12 $MWh^−1^ for onshore wind, 130 ± 11 $MWh^−1^ for offshore wind) exceeded other reported values for all-renewable or net-zero CO_2_ UK electricity (c. 60–100 $MWh^−1^)^13,57^, with considerable room for further improvement by incorporating other energy storage technologies in parallel with hydrogen and TCES, and interconnection between windfarms (albeit with challenges associated with electricity transmission capacity). Alternatively, given the system was scaled to maximise wind utilisation and minimise curtailment, capital expenditure could be decreased by operating with lower electrolyser capacity—in effect, sacrificing some theoretically available energy on days with the strongest wind in order to decrease overall system cost^6^ (with examples of the effect of deliberately curtailing output on the 50 days with highest capacity factor shown in Supplementary Note 7 and Supplementary Fig. S17).

Furthermore, we assumed that seasonal variation in electricity consumption will remain approximately in line with historic trends, without significant changes in user demand patterns, an assumption which might not be valid in conjunction with current UK plans to achieve net-zero emissions countrywide^58^. For example, future decarbonisation of domestic heating is likely to result in much greater seasonal variation in electricity demand^59,60^, as current demand for natural gas is displaced by e.g., electrically-powered heat pumps. Therefore, seasonal variation in heating energy demand for a location in the UK was estimated for the period 01/01/2016-31/12/2020^61^. Variation in total electricity demand was adjusted to account for electrified domestic heating, at a constant ratio of domestic energy consumption used for heating as compared to all other uses (c. 4:1)^62^ and no improvements in domestic energy efficiency (i.e., representing a plausible worst-case scenario for domestic heat consumption), shown in Supplementary Note 7, Supplementary Fig. S14, with a minimum demand of 3600 kWh d^−1^ in summer, and a maximum of 36,800 kWh d^−1^ in winter (while maintaining an annual average of 20,000 kWh d^−1^).

Increased seasonal variation in energy demand to account for domestic heating was considered for an onshore system (with *f*_*OCP*_ = 0.25 and 6 days of H_2_ storage, under base-case modelling assumptions), with the results shown in Supplementary Note 7 and Supplementary Fig. S15. The larger seasonal variation in demand outweighed any seasonal variation in supply, with the hydrogen and heat storage rapidly filling at the start of summer, remaining full through summer, high summer, and autumn, and then rapidly depleting at the start of winter, with extensive grid imports required in order to supply electricity for the remainder of winter. Therefore, base-case LCOE increased to 160 $MWh^−1^, and net carbon intensity became positive, at 75 $${{{{\rm{kg}}}}}_{{CO}_{2}}\,{{{{\rm{MWh}}}}}^{-1}$$, indicating a limit to the capability of the system to achieve net-zero operating emissions under conditions of extreme (tenfold or greater) seasonal variability in demand.

## Discussion

In order to function, the process described here requires a feed of relatively concentrated (> 5 vol%) CO_2_ to drive the carbonation reaction MgO + CO_2_ → MgCO_3_ and generate heat for H_2_ release. Therefore, the system cannot be considered to achieve true net-negative emissions in terms of removing CO_2_ already present in the atmosphere. Rather, the system proposed here is a technology for partial capture of hard-to-abate CO_2_ emissions as an alternative or complement to conventional flue gas post-combustion carbon capture^39,49^, with subsequent connection to geological sequestration (noting that the costs of sequestration were beyond the scope of the system boundary investigated here).

The system we describe in this work comprises a combined system for converting the intermittent power output from a wind farm into a continuous power supply to consumers, using hydrogen storage to manage fluctuations. Assuming relatively efficient electricity transmission via the power grid, the long-term energy storage would not need to be co-located with the windfarm, provided that storage capacity and backup gas turbine capacity were allocated in an appropriate ratio to windfarm output. However, in order to have access to sufficient flue gas, the hydrogen storage system does need to be co-located with an otherwise-unabated CO_2_ point source (e.g., a steel or cement plant). Such suitable sites in the UK could be at one of the industrial clusters identified by the UK government as suitable for carbon capture projects, which are responsible for c. 50% of non-power related UK industrial CO_2_ emissions (c. 36.1 Mt y^−1^)^63–66^. Alternatively, the hydrogen storage could be colocated with the gas-fired turbines used to meet energy demand during sustained periods of low wind, by scheduling hydrogen release and gas turbine operation to operate simultaneously, or, in order to achieve true carbon drawdown from the atmosphere, partially concentrated output from a direct air carbon capture system could be used as a CO_2_ source rather than industrial flue gas, albeit again incurring a penalty on overall energy efficiency.

Additionally, we assumed a relatively low per-pass CO_2_ capture efficiency of 50% for the MgL process, in line with previous experimental research^38^, meaning the remaining 50% of the CO_2_ contained within the flue gas passed through the system without being captured. Given the relatively low thermodynamic efficiency of MgL carbon capture at 350 °C^39^, increasing conversion decreased round-trip energy efficiency, as a result of requiring higher inlet reactor pressure during carbonation. Therefore, 50% capture efficiency represented a near-optimal value for balancing reactor pressure and throughput of flue gas, as shown in Supplementary Note 4 and Supplementary Fig. S4. In order to improve the overall CO_2_ capture efficiency of the system, further downstream carbon capture could be added using a more thermodynamically-efficient sorbent (e.g., CaO-CaCO_3_ looping, or amine-based CO_2_ scrubbing), at the cost of decreased energy efficiency.

For the most cost-effective system configuration investigated here (*f*_*OCP*_ = 0.3, 2 days of H_2_ storage), 6–10% of total electricity output was supplied from external grid imports (i.e., from natural gas combustion, shown in Supplementary Note 6 and Supplementary Table S9), thereby requiring maintenance of existing fossil fuel infrastructure. While some offshore system configurations were able to achieve stable power output from wind and stored H_2_ with no energy imports from gas turbines (shown in Supplementary Note 6 and Supplementary Fig. S8), overall cost of electricity increased by c. 30%, and very high windfarm capacities (≥100% overcapacity) were required, resulting in a increase in overall land use and fraction of curtailed (i.e., wasted) power.

As hydrogen was used to balance all variations in electricity supply longer than 1 day, the average residence time of stored hydrogen for offshore and onshore wind was 28–30 days. Therefore, the overall efficiency of the energy storage system would be improved by incorporating a secondary energy storage system with higher round-trip efficiency, such as compressed or liquefied air storage (CAES/LAES)^67^, or stand-alone thermo-chemical electricity storage^68^, to manage daily and weekly fluctuations in supply within each season, allowing hydrogen to be reserved for inter-seasonal and inter-annual balancing only^12,13^. The role of weather prediction in managing hydrogen production and storage was also not considered here, with a simple on-off control scheme used to manage energy storage and release. Future work should consider more sophisticated control models incorporating the inherent uncertainty in weather prediction^69^, especially for optimisation of a system with more than one energy storage system (e.g., CAES or LAES operating in parallel with hydride H_2_ storage). Additionally, the system couples two relatively slow chemical reactions (hydrogen sorption and MgO carbonation) with turbomachinery for gas compression, each with different characteristic start-up and shut-down times, and with lag from turbomachinery, introducing significant difficulties in process control, especially during the transition periods between carbon capture and CO_2_ release. While directly modelling the relative rates of each process was beyond the scope of this study, future work should consider the design of control systems able to allow the system to cope with varying load over time, incorporation of additional short-term electricity storage in the form of batteries, and short-term compressed gas storage using buffer tanks.

Moreover, given the length of the modelled energy supply period of five years used in the study was relatively short as compared to the assumed system lifetime, the minimum storage capacities estimated here represent a lower-bound estimate, with greater storage capacity required to tolerate once per decade or less frequent low wind weather events^13,70^ without having to implement load-shedding.

Given the relatively low gravimetric energy capacity of MgH_2_ relative to gaseous or liquid H_2_ storage, a major challenge for the system we propose here is the large quantity of magnesium metal required to produce the hydrogen storage alloy. Implementing 2 days of H_2_ storage capacity for existing UK onshore wind infrastructure (c. 377 GWh d^−1^) would require approximately 300,000 tonnes of refined Mg, or around 30% of current annual production^37^. Although in principle a near-unlimited supply of magnesium hydroxide could be obtained from seawater brine^71^, the energy costs and carbon emissions associated with refining magnesium ores to magnesium metal markedly increase the net lifetime emissions of the system^37^, as shown in Supplementary Note 6 and Supplementary Fig. S9, where only a narrow span of system configurations were able to achieve net-zero emissions. Therefore, unless the MgL subsystem we propose here were incorporated to achieve CO_2_ drawdown, the CO_2_ emissions associated with Mg metal refining would likely outweigh any reduction in carbon operating emissions achieved by using solid-state hydrogen storage.

Uncertainties in the cost and lifetime of the Mg-based alloys, and the PEM fuel cell, contribute to the wide spread of estimated CAPEX and OPEX costs shown in Fig. 7, exacerbated by historic time and cost overruns in green hydrogen and offshore wind projects^19,72,73^. Hence, incremental improvements in both the capacity and durability of Mg alloys for H_2_ storage, and in the lifetime of PEM stacks under intermittent operation, have a marked impact on the overall feasibility of the system (as shown in Supplementary Note 7 and Supplementary Fig. S12), with considerable opportunities to improve system cost with future research and development.

To conclude, in this work, we proposed a combined system to help overcome an inherent limitation of intermittent wind power by using thermally-efficient solid hydrogen storage to balance seasonal fluctuations in daily electricity output. A MgL carbon capture system acts as a thermo-chemical heat source or sink for the hydrogen storage reaction, and simultaneously offsets some of the emissions associated with the use of dispatchable gas turbines to supplement stored energy, thereby avoiding the need for unrealistically large wind farm or energy storage capacities. While not a complete solution for achieving fully decarbonised power generation, the proposed system could help act as a transitional step to allow for greater renewables integration while offsetting residual gas turbine usage, with limited risk of becoming a stranded asset, provided hard-to-abate CO_2_ industrial point sources remain available, or, with systems adapted in future to operate on a closed loop with CO_2_ storage.

We estimate an overall LCOE of 121 ± 12 and 130 ± 11 *$*MWh^−1^ for onshore and offshore windfarms, respectively, comparable with existing gas turbine infrastructure (c. 144 *$*MWh^−1^)^74^, and at around  half the cost of an equivalent capacity storage system for surplus wind energy using grid-scale Li-ion batteries only (c. 250 *$*MWh^−1^). The use of carbonate-based thermo-chemical energy storage with MgO-MgCO_3_ looping simultaneously improves the theoretical round-trip efficiency of the system relative to hydrogen storage alone, and decreases overall emissions associated with power generation by capturing CO_2_ from flue gas. Additionally, by using the MgL loop to offset carbon emissions from gas turbine power generation during periods of limited wind, the system is able to achieve net-zero CO_2_ emissions with considerably lower generation or storage overcapacity than would be required for an all-renewable system, with potential for converting the system towards zero-import operation over time with expanding renewables overcapacity.

Of the energy storage systems considered here, combined hydride-based H_2_ storage with carbonate-based heat recovery was the only system able to achieve net-zero operating emissions from wind power using real-world wind speed data, while maintaining stable electricity delivery to users and achieving cost-competitiveness with non-intermittent fossil fuel power sources.

## Methods

### Experimental methods

The hydrogen storage and release properties of a Mg-based alloy (76.5 wt% Mg, 8.5 wt% Al, 10 wt% Ni, 5 wt% C) were investigated using a Sieverts-type volumetric hydrogen absorption reactor^75^, with details given in Supplementary Note S3.1. Pressure-composition-temperature (PCT) isotherms were collected by introducing H_2_ gas at known pressure, produced from deionised water using a ThalesNano H-Genie hydrogen generator (producing gas with nominal purity 99.99 vol% H_2_). The hydrogen inlet pressure was then increased incrementally up to 20 bar until hydrogen uptake reached a plateau, indicating the equilibrium pressure of hydrogen for the set temperature. Cyclic hydrogen absorption-desorption measurements were collected by heating the reactor to a given setpoint temperature, then adjusting the partial pressure of hydrogen to 20 bara for 30 min during the absorption step, followed by purging with He (BOC, 99.999%) for 30 min during the desorption step.

The carbonation and calcination properties of a MgO-based material modified with an alkali metal nitrate catalyst, prepared using a modification of a literature procedure^76^, were investigated by thermogravimetric analysis, with full details given in Supplementary Note 3. Temperature programmed carbonation and decarbonation measurements over three cycles were collected using a Metter Toledo TGA/DSC 3+ thermogravimetric analyser (TGA). During carbonation, a sample (~ 20 mg) of MgO-based material was heated from 50 to 500 °C at 2 °C min^−1^ under flowing CO_2_ (50 mL min^−1^, BOC, 99.99+%), with purge and protective flow of N_2_ through the TGA chamber (each 50 mL min^−1^, BOC, 99.999+%), giving a nominal *pCO*_2_ of 0.33 bara. During calcination, the sample was cooled from 500 to 50 °C at −2 °C min^−1^ under N_2_ flow (50 mL min^−1^, nominal *pCO*_2_ < 10^−5^ bara).

### Modelling

The systems described were modelled using a collection of MATLAB scripts, with process flow diagrams, a description of the daily energy allocation algorithm, estimation of electricity generation and demand, and relevant modelling assumptions for each unit operation given in Supplementary Note 4.

#### Estimation of electricity demand

Average daily electricity demand was set at an arbitrary nominal overall load, $\overline{L}$, of 20,000 kWh d^−1^ averaged over a full year (corresponding to around 3000 domestic users, or a medium-scale industrial plant). Seasonal variation in demand was estimated using electricity generation data reported by the National Energy System Operator^77^ for the period 2016–2020. Each year was modelled as five seasons of unequal length (spring, summer, high summer, autumn, and winter)^78^, assuming constant relative demand for every day in each season equal to the five-year average demand for that season, as described in Supplementary Note 4 and Supplementary Table S3. Actual energy demand for each modelled day was estimated using ${L}_{i}=\overline{L}\cdot {s}_{seas}$, where *L*_*i*_ is the total demand (kWh) on day *i*, and *s*_*seas*_ is the seasonal demand variation factor for day *i*, given in Table S3. Alternative demand modelling cases are shown in Supplementary Note 7 and Supplementary Figs. S13–S15.

#### Estimation of electricity supply

Electricity was assumed to be generated from either an onshore or offshore windfarm. The daily capacity factor for each windfarm location, defined as the ratio of average power output on a given day to nominal windfarm nameplate capacity, was estimated using the Renewables Ninja web application^61,79^. Daily power generation was estimated for a Vesta V80 2000 turbine with hub height 100 m, using the NASA MERRA-2 global wind speed database^80^ over the period 01/01/2016-31/12/2020. Further modelling assumptions used to estimate wind power generation are described elsewhere^5^.

Nominal nameplate capacity, *w*_*nameplate*_ (kWh d^−1^), for the windfarm in each modelled scenario was set by scaling the windfarm power output according to the average daily capacity factor, $\overline{CF}$, for the selected location over the five-year modelling period, with the addition of an overcapacity factor, *f*_*OCP*_, corresponding to the fraction of net surplus power relative to estimated demand, shown in Eq. (2) (i.e., an overcapacity factor of zero would correspond to a windfarm where total energy generation over five years was exactly equal to total demand, whereas an overcapacity of 0.2 would result in a net surplus of 20% relative to total modelled demand).

2 $${w}_{nameplate}=\frac{\overline{L}}{\overline{CF}}\cdot \left(1+{f}_{OCP}\right)$$

In the event of a net shortfall in total energy generation from the wind farm and stored H_2_ relative to demand on any given day, supplementary energy was assumed to be available from the electricity grid, with marginal energy generation supplied by combustion of natural gas in a CCGT power plant without post-combustion carbon capture. The cost of operation for the gas turbines was calculated based on the estimated LCOE for UK power plants commissioned in 2021^74^, with an additional cost added to account for carbon taxation, using an estimated cost of carbon in the range 100–200 $$$\,{{{{\rm{t}}}}}_{{CO}_{2}}^{-1}$$^81,82^, adjusted according to modelling optimism, with a higher carbon tax (and so, higher operating costs) for the pessimistic case.

#### System efficiency and costing

A simplified process flow diagram showing the main unit operations for the modelled hydrogen storage system is shown in Supplementary Note 4 and Supplementary Fig. S3. Key modelling assumptions for estimating the sizing and operation of each unit are described in Supplementary Note 4, with all calculations performed using a collection of MATLAB scripts. All reactions were modelled as 0D processes, and assumed to be sufficiently rapid to be limited by thermodynamic equilibrium over each 24 h time step^83^, in order to estimate theoretical system performance for different configurations. To achieve a driving force for heat transfer and reaction, a temperature difference between the reactors is necessary, i.e., during hydrogen storage, the operating temperature of the exothermic reaction Mg + H_2_ → MgH_2_ slightly exceeds the operating temperature of the endothermic calcination reaction MgCO_3_ → MgO + CO_2_ (and vice versa during hydrogen release). An average temperature offset (*T*_*drive*_) between reactors of 5 °C was used for thermodynamic calculations and for sizing heat transfer equipment. Ambient heat losses (estimated in Supplementary Note 4) were compensated for by introducing additional heat to the system using an electrical heater.

The energy efficiency of hydrogen storage and release were calculated using Eqs. (3) and (4) (with full derivations given in Supplementary Note 4), where *ξ*_*in*_ is the net conversion efficiency of electricity into stored hydrogen (${{{{\rm{kg}}}}}_{{H}_{2}}\,{{{{\rm{kWh}}}}}^{-1}$), *ξ*_*out*_ is the net conversion efficiency of stored hydrogen into electricity (${{{\rm{kWh}}}}\,{{{{\rm{kg}}}}}_{{H}_{2}}^{-1}$), *ξ*_*elec*_ is the electrical efficiency of the electrolyser (${{{{\rm{kg}}}}}_{{H}_{2}}\,{{{{\rm{kWh}}}}}^{-1}$), *ξ*_*fc*_ is the electrical efficiency of the fuel cell (${{{\rm{kWh}}}}\,{{{{\rm{kg}}}}}_{{H}_{2}}^{-1}$). The terms *w*_*flue*_ and *w*_*pipeline*_ (both kWh kg^−1^) are the compression duties for bringing flue gas to reactor pressure, and compressing pure CO_2_ produced to 150 bara respectively, *w*_*expand*_ is the energy recovery (kWh kg^−1^) achieved during subsequent expansion of the reactor exhaust gas, and $\Delta {H}_{{H}_{2}}$ and $\Delta {H}_{C{O}_{2}}$ are the enthalpies of reaction for MgH_2_ → Mg + H_2_ and MgCO_3_ → MgO + CO_2_ (${{{\rm{kWh}}}}\,{{{{\rm{kg}}}}}_{{H}_{2}}^{-1}$ and ${{{\rm{kWh}}}}\,{{{{\rm{kg}}}}}_{{CO}_{2}}^{-1}$ respectively). The term ${X}_{{CO}_{2}}$ is the per-pass conversion of CO_2_ in the carbonation reactor (i.e., the CO_2_ capture efficiency from flue gas), such that the partial pressure of CO_2_ in the reactor outlet was at pCO_2,*eq*_ for the specified reactor temperature, and ${Y}_{C{O}_{2}}$ is the mass fraction of CO_2_ in the flue gas feed. The heat transfer term *q*_*PH*,*i*_ is the energy required to preheat gas *i* to reactor temperature (kWh kg^−1^), and *q*_*cool*_ (${{{\rm{kWh}}}}\,{{{{\rm{kg}}}}}_{{H}_{2}}^{-1}$) is the heat recovery from the released hydrogen.

3 $${\xi }_{in}=\frac{{\xi }_{elec}}{1+{\xi }_{elec}\left(\frac{\Delta {H}_{{H}_{2}}-{q}_{cool}}{\Delta {H}_{{CO}_{2}}-{q}_{PH,C{O}_{2}}}({w}_{pipeline}+\Delta {H}_{C{O}_{2}})-(\Delta {H}_{{H}_{2}}-{q}_{PH,{H}_{2}})\right)}$$

4 $${\xi }_{out}={\xi }_{fc}-\frac{\Delta {H}_{{H}_{2}}-{q}_{cool}}{\Delta {H}_{C{O}_{2}}-{q}_{PH,C{O}_{2}}}\left(\frac{{w}_{compress}-(1-{X}_{C{O}_{2}}{Y}_{C{O}_{2}}){w}_{expand}}{{X}_{C{O}_{2}}{Y}_{C{O}_{2}}}\right)$$

The round-trip efficiency (*η*_*RT*_) of the system was calculated using Eq. (5)

5 $${\eta }_{RT}\,=\,{\xi }_{in}\cdot {\xi }_{out}$$

For a simple pass-through system where electricity is converted into hydrogen in the electrolyser, then immediately converted back into electricity in the fuel cell, the maximum theoretical round-trip efficiency (i.e., neglecting all energy penalties associated with H_2_ storage) is given by Eq. (6)

6 $${\eta }_{passthrough}\,=\,{\xi }_{elec}\,\cdot \,{\xi }_{fc}\,=\,0.24$$

for the base-case assumptions used here (given in Supplementary Note 5 and Supplementary Table S5). We also defined the storage efficiency, *η*_*store*_, using Eq. (7), corresponding to the relative energy efficiency of the storage system as compared to the overall round-trip efficiency (i.e., the fraction of total energy losses in the electrolyser and fuel cell).

7 $${\eta }_{store}=\frac{{\eta }_{RT}}{{\eta }_{passthrough}}$$

Values of *pCO*_2_ at equilibrium for the MgO-MgCO_3_ system were calculated from the FactPS thermodynamic database using FactSage software^47^ for system operation in the temperature range 300–400 °C, previously shown to provide reasonably accurate alignment with experimental measurements^39^. Values of *pH*_2_ at equilibrium for Mg-MgH_2_ were measured experimentally for a Mg-based hydrogen storage material as shown in Fig. 2.

The LCOE ($MWh^−1^) was estimated for a 25 year period of system operation^74^, by calculating the net present value of capital and operating expenditure, and the total expected energy load over the system lifetime using Eq. (8), where CAPEX is the total initial capital expenditure, OPEX_*i*_ is operational expenditure in year *i*, and *f*_*discount*_ is the discount rate used to convert future expenditure to the present day, assumed to be approximately equal to the weighted average cost of capital used to finance construction. Costs of windfarm construction and maintenance were included in estimates of CAPEX and OPEX, excluding the cost of land. Decommissioning costs and scrap value of materials and process equipment were not considered for the techno-economic model.

8 $${{{\rm{LCOE}}}}=\frac{{{{\rm{CAPEX}}}}+{\sum }_{i=1}^{25}\frac{{{{{\rm{OPEX}}}}}_{i}}{{(1+{f}_{discount})}^{i}}}{{\sum }_{i=1}^{25}\frac{L}{{(1+{f}_{discount})}^{i}}}$$

Capital and operating costs were estimated using reported values from literature (shown in Table 1, with additional parameters given in Supplementary Note 5 and Supplementary Tables S5 and S16), using the mean of recent reported values as the base-case estimate, and maximum and minimum reported values for the optimistic and pessimistic cases as appropriate (i.e., maximum lifetime, minimum cost for the optimistic case and vice versa for the pessimistic case). For input parameters with limited available data, or common items of process equipment (e.g., turbomachinery), approximate cost estimation correlations from literature were used^84^ where available, adjusted for inflation using the Chemical Engineering Plant Cost Index^85^. To quantify the uncertainty in estimated system parameters, Monte Carlo simulation was performed by randomly sampling input parameters over the reported range, as described in Supplementary Note S5.

Additional modelled parameters and cases, not reported in the main manuscript, are compiled in Supplementary Note 6, Supplementary Figs. S7–S9, and Supplementary Tables S8 and S9.

The sensitivity of the model to estimated input parameters, and the validation of the modelling assumptions used, are discussed in Supplementary Note 7 (Supplementary Tables S10–S15 and Supplementary Figs. S10–S17), and descriptions of models used for alternative system configurations are given in Supplementary Note 8 (Supplementary Figs. S18 and S19 and Supplementary Table S16).

## Supplementary information

Supplementary Information

Transparent Peer Review file

## Source data

Source Data

## Data Availability

The MATLAB code and datasets used to generate the findings of this study have been deposited in the Code Ocean platform (10.24433/CO.5706678.v3). Raw input energy demand data and wind capacity factor data were downloaded from the National Energy System Operator web portal^77^ and the Renewable Ninja web application^61,79^, respectively. The data generated in this study have been deposited in the Source Data files, included as part of the Supporting Information. Any other relevant information can be provided by the corresponding authors upon request by email (a.r.harrison@imperial.ac.uk and binjian.nie@eng.ox.ac.uk). Source data are provided with this paper.

## Code availability

The MATLAB code and wind capacity factor datasets used to generate the findings of this study are available via the Code Ocean platform (10.24433/CO.5706678.v3).
