# Supplementary material for: Thermally coupled solid hydrogen storage and carbon capture for balancing intermittent renewable energy
Source: Nat Commun. 2026 Apr 21;17:5514. doi: 10.1038/s41467-026-72035-1 (PMC13287580; doi:10.1038/s41467-026-72035-1)
Supplement: Supplementary file 1 — Supplementary Information [file 41467_2026_72035_MOESM1_ESM.pdf]

Supplementary information for:

Thermally coupled solid hydrogen storage and carbon capture for balancing intermittent renewable energy

Alexander R.P. Harrison[1,2*], George J. Fulham[3], Haoliang Hong[1] and Binjian Nie[1*]

[1]Department of Engineering Science, University of Oxford, Parks Road, Oxford, OX1 3PJ, United Kingdom.
[2]*Present address:* Department of Chemical Engineering, Imperial College London, South Kensington, London, SW7 2AZ, United Kingdom.
[3]Department of Chemical Engineering and Biotechnology, University of Cambridge, Philippa Fawcett Drive, Cambridge, CB3 0AS, United Kingdom.

*Corresponding author(s). E-mail(s): a.r.harrison@imperial.ac.uk; binjian.nie@eng.ox.ac.uk;
Contributing authors: gf325@cam.ac.uk; haoliang.hong@reuben.ox.ac.uk;

1 **Supplementary Note 1: Variability in wind capacity factor** **S4**

2 **Supplementary Note 2: Overview of relevant prior studies** **S5**

S2.1 Combined solid-state hydrogen storage and heat recovery . . . S5

S2.2 Intermittency management for renewable energy S10

3 **Supplementary Note 3: Additional experimental information** **S18**

S3.1 Synthesis and characterisation of H_2 storage material S18

S3.2 Synthesis and characterisation of MgO-based TCES material . S19

4 **Supplementary Note 4: Model description and assumptions** **S20**

S4.1 System operation S22

S4.2 Polymer electrolyte membrane electrolyser and fuel cell S28

S4.3 Hydrogen storage and carbonation reactors S29

S4.4 Backup gas turbines S31

S4.5 Heat exchange equipment S31

S4.6 Compressors and turbo-expanders S32

S4.7 Battery subsystem S35

S4.8 Derivation of expressions for round-trip efficiency S37

S4.9 Configurations without heat storage S41

5 **Supplementary Note 5: Additional parameters for techno-economic analysis** **S42**

S5.1 Estimation of embedded emissions S43

6 **Supplementary Note 6: Additional modelled parameters and cases** **S45**

7 **Supplementary Note 7: Model sensitivity** **S51**

S7.1 Sensitivity to input parameters: Monte Carlo simulation S51

S7.2 Sensitivity to demand model S54

S7.3 Sensitivity to initial conditions S57

S7.4 Sensitivity to windfarm location S59

S7.5 Sensitivity to year order S62

S7.6 Sensitivity to days with lowest and highest wind speeds S64

8 Supplementary Note 8: Alternative system configurations S66

S8.1 Thermochemical energy storage using magnesium hydroxide . . S66

S8.2 Storage of hydrogen as a compressed gas or cryogenic liquid . . S69

1 Supplementary Note 1: Variability in wind capacity factor

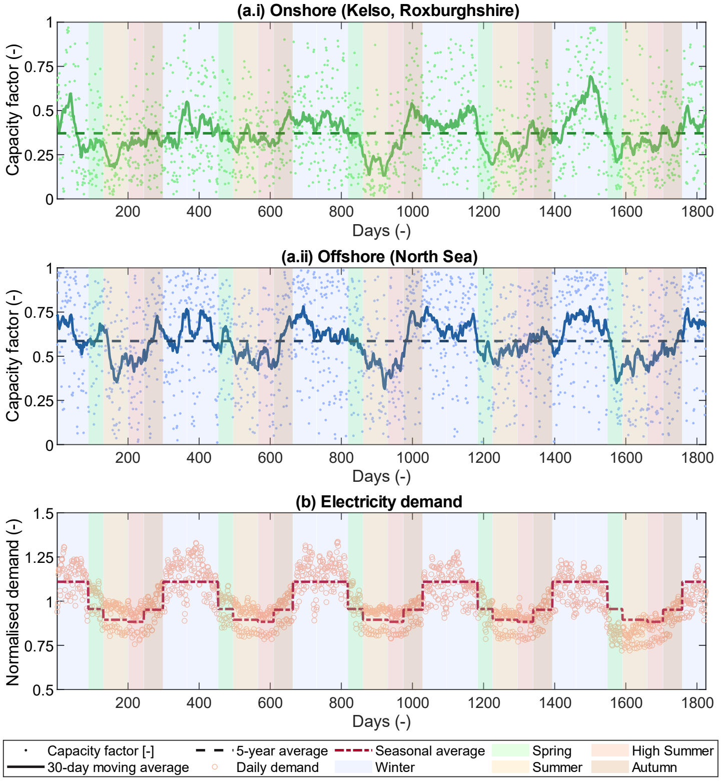

Supplementary Figure S1 **(a)** Variation in estimated daily capacity factor for two windfarm locations, over the period 2016-2020: **(i)** onshore location near Kelso, Roxburghshire, and **(ii)** offshore location in the North Sea. Points indicate daily capacity factor, solid line indicates 30-day moving average, and dashed line indicates overall average over the five year period. **(b)** Estimated seasonal variation in UK electricity demand over the same period, normalised against average annual demand [1, 2]. Shaded regions correspond to seasons. Note that unlike solar panels, capacity factors for windfarms for Northern Europe typically peak in winter, in line with seasonal electricity demand.

2 Supplementary Note 2: Overview of relevant prior studies

Here, we provide an overview of previous studies relevant to the work discussed in the main article, in particular, previous studies on solid hydride H_2 storage and combination with thermal or thermo-chemical energy storage (given in Table S1), and energy storage systems for balancing variable daily power output from renewables (given in Table S2, excluding systems based solely on batteries, or pumped hydroelectric storage). The list of works cited here is not intended to be exhaustive, but rather provides a selection of different approaches and perspectives from recent literature.

S2.1 Combined solid-state hydrogen storage and heat recovery

Supplementary Table S1: Recent research considering systems for combined solid-state hydrogen storage and heat recovery, and related research

Work(s) and topic	Summary	Key findings
**Practical design of hydride-based hydrogen storage reactors: Fruchart *et al.* (2023) [3], and works cited therein**	• Description of production methods for Mg-based hydrides for H_2 storage, with addition of expanded graphite to aid heat transfer.	• Externally heated vessel able to fully charge or discharge within ∼3-4 h at 300-350°C.
	• Modular reactor designs (scalable based on application, designed and constructed with McPhy Energy and JOMI LEMAN) for kg-scale H_2 storage (*c.* 100 kg hydride per vessel, TRL 7),	• Relatively poor thermal conductivity of MgH_2 can also impact performance as reaction becomes heat-transfer limited.

	• Storage composed of compacted discs of Mg-graphite composite, with external heat exchange using circulating heat-transfer oil, or, with concentric heat storage section for 'adiabatic' operation, using molten metal PCMs (Mg-Zn eutectic alloy).	• Authors suggest integrating externally heated reactor with heat demand/waste heat from processes using H_2, with chemical and metallurgic applications suggested.
	• Reactor outlet during H_2 desorption connected to PEM fuel cell, with power output able to follow hydrogen release.	• 'Adiabatic' system with PCM heat recovery proposed for on-demand H_2 storage delivery, with proposed applications in intermittent green H_2 production.
**Demonstration scale hydrogen storage reactor: Ren *et al.* (2023)** [4]	• Mobile pilot-scale H_2 storage system (designed and constructed with HyFun Energy Technology Co.) using pellets of Mg-Ni based alloy (*c.* total 1 t stored H_2, 14.4 t alloy, TRL 7) without heat recovery.	• Reactor compared with compressed H_2 for mobile applications, with *c.* 3× greater volumetric energy density.
	• Heat transfer using integrated tubular heat exchanger with heat transfer oil, heated using combustion of fuel oil.	• System able to charge or discharge within c.12 h, with estimated output of 1.6-3.2 MWh of electricity using a PEM fuel cell.

	• Use of heat recovery using molten salt PCMs, or MgO-Mg$(OH)_2$ TCES suggested for stationary applications, but no measured or calculated efficiencies reported.	• Stable hydrogen absorption and desorption for up to ~3,000 cycles.
**Experimental demonstration of coupled hydride storage with hydroxide TCES: Lutz *et al.* (2020) [5] and works cited therein**	• Bench-scale packed bed reactor experiments combining MgH_2 hydrogen storage (using 234 g of a Mg-based alloy with 10wt% Ni) with a concentric bed of MgO-Mg$(OH)_2$ TCES material (TRL 3-4).	• Steam pressure of 10 bar required at reactor operating temperature of 300°C (*i.e.* superheated).
	• Hydrogenation and dehydrogenation performed in separate steps, with radial temperature distribution through hydride and TCES beds monitored.	• Use of TCES material during hydrogen absorption resulted in *c.* 10°C lower temperature overshoot in external concentric bed, as a result of endothermic reaction Mg$(OH)_2$ $\rightarrow$ MgO + H_2O.
	• Waste heat from PEM used to boil water to 180°C, with energy for superheating supplied from stored heat in MgO and external electrical heater.	• TCES reactions became kinetically limited during 1-2 h hydration-dehydration steps, limiting heat (and hence, H_2) available for reaction.
	• Vapour pressure of steam feed or condensate output used to control temperature of reactions.	• Hysteresis observed between MgO hydration and dehydration at 9 bar, resulting in lower operating temperature than expected during hydration.

		• Prototype reactor showed volumetric H_2 density of 0.2 gH_2 L^{-1}, *c.* 100 times lower than the theoretical maximum for MgH_2 and MgO-$Mg(OH)_2$.
**Geometrical optimisation of PCM heat recovery from hydride storage: Maggini *et al.* (2024, 2025) [6, 7]**	• Computational studies of concentric metal hydride and PCM beds.<br>• System described using 11 non-dimensional parameters.<br>• Reactor design adjusted depending on physical properties of hydride and PCM, with different optimal solutions for e.g. $LaNi_5$ vs Mg_2Ni.	• Non-dimensional analysis indicated that a high reactor aspect ratio, and high thermal conductivity of the PCM, are necessary to achieve theoretical H_2 storage capacity without heat transfer limitation.<br>• Model assumes natural convection within molten PCM; not applicable for TCES without modification.
**Geometrical optimisation of PCM heat recovery from hydride storage: Mehrpooya and Zhalehrajbi (2024) [8]**	• Computational study of radially segmented packed bed of Mg_2NiH_4 and $NaNO_3$ PCM, with central H_2 channel.<br>• Additional concentric PCM layer added, surrounding segmented bed.	• Simulated 'cold-start' autothermal operation: hydride bed heated to operating temperature within 10 s.<br>• Hydrogenation reaction reached approximately 12% conversion, but became heat transfer limited as energy storage capacity of PCM was exhausted within ∼200 s.<br>• Similarly, during dehydrogenation, molten PCM in segmented channels became fully solidified within 100 s, limiting hydride conversion to ∼40%.

		• Additional PCM in a concentric layer was required to fully dehydrogenate hydride, giving an overall hydride:PCM volume ratio of around 1:9.
**Modelling hydroxide TCES heat recovery from hydride storage: Shi *et al.* (2022) [9, 10]**	• Computational study on hydrogen storage in MgH_2, with concentric bed of MgO-$Mg(OH)_2$ TCES.<br>• Reactors directly coupled with no heat transfer fluid or external heating.	• Reactor able to charge with H_2 within 2 h, and discharge within ∼9 h.<br>• Gas permeability within hydride, and thermal conductivity of MgO, limited overall performance.<br>• Temperature in reactor controlled by adjusting water vapour pressure in TCES section.<br>• Temperature in hydride bed rises or falls rapidly during hydrogenation and dehydrogenation respectively once energy storage capacity of TCES bed is exhausted.
**Experimental demonstration of heat recovery from low-temperature hydrides: Nyamsi *et al.* (2023, 2024) [11, 12]**	• Computational and experimental study using low temperature hydrides ($Ti_{0.55}Zr_{0.45}(FeCrMnNiV)_2$, operating at ∼25°C), with reaction vessel immersed in coconut oil PCM.	• Higher temperature PCMs (*i.e.* nitrates, eutectic metals) give faster H_2 charge and discharge times than organic PCMs, by virtue of having higher energy density.
**Modelling magnesium looping TCES for process heat recovery: Tregambi et al. (2023) [13]**	• Process model of magnesium looping carbon capture, coupled to CO_2 hydrogenation to methane.	• Higher carbonation temperature (in the range 275-325°C) decreases carbon capture efficiency from 97.7% to ∼80%.

	• Carbonate-based TCES (MgO-$MgCO_3$) used as a a CO_2 sorbent and chemical heat pump for autothermal CO_2 capture from flue gas and subsequent methanation.<br>• Heat released from CO_2 methanation used to calcine $MgCO_3$ in a closed-loop system.	• System efficiency decreased by lower CO_2 concentration in feed, but was able to reach a pseudo-autothermal state for all conditions, with excess heat generated in methanation reaction at 350-450°C.<br>• External H_2 input stream required for process to function.

S2.2 Intermittency management for renewable energy

Supplementary Table S2: Recent research considering renewable energy storage using H_2 and other emerging technologies

Work(s) and topic	Summary	Key findings
**Grid-scale modelling of UK energy storage: Royal Society (2023) [14], and works cited therein**	• Report concerning large-scale electricity storage strategies for Great Britain, considering a wide range of different storage technologies.<br>• Energy supply and consumption modelled over a 37 year period; combinations of multiple energy storage systems optimised to minimise cost.<br>• Metal hydrides not discussed or considered among H_2 storage methods.	• Inverse correlation between storage duration and round-trip efficiency: indicating a mix of storage methods required for different timescales.<br>• Recommends compressed H_2 storage in salt caverns as most cost-effective long term storage method based on UK geology, followed by chemical H_2 storage as NH_3.<br>• Batteries used for balancing short term (<1 day) energy fluctuations, and to provide grid services (e.g. frequency regulation).

		• Inter-annual balancing to account for decadal variation in renewable power necessary: some H_2 maintained in storage for >20 years. Storage estimates based on a single year of data can underestimate necessary capacity by up to 100%.
		• Compressed air storage proposed for 'medium-term' grid balancing (*i.e.* storing energy for 2–3 weeks) to bridge the gap between batteries and H_2.
		• Overall electricity cost estimated in range 50–85 £/MWh depending on modelling assumptions.
**Modelling interactions between long- and short-term storage: Xing *et al.* (2024)** [15]	• Agent-based model for allocating UK energy generation with variable renewable energy and storage.	• Thermal generation, interconnectors, and energy storage compete with one another.
	• Windspeed data used to estimate maximum potential wind power capacity for offshore locations.	• System flexibility removed by deactivating thermal generation is not compensated by additional renewables capacity without storage, resulting in 1–5 blackouts *per* year.
	• Storage systems defined as either 'pumped hydro-like' (operational life independent of cycles) or 'battery-like' (storage degrades with each power cycle).	• Thermal generation cost-competitive with stored hydrogen for providing seasonal flexibility, but transition from baseload to flexible operation increases costs due to frequent start up.

		• Lowest system cost achieved with *c.* 25 GW of energy storage, with a roughly equal parts mixture of pumped hydro, compressed air, batteries, and green hydrogen, for use over different timescales.
**Modelling combined wind and wave power with compressed air storage: Kluger *et al.* (2023)** [16]	• Modelling the integration of floating offshore wind colocated with hydropower from wave energy converters (WEC).	• Supplemental use of WEC provides electricity with steady diurnal variation to support wind power, but results in 3-fold increase to capital cost.
	• Compressed air energy storage (CAES) utilised for power balancing.	• The overall reliance on CAES is not alleviated by increasing the fraction of WEC relative to wind power.
**Hydride hydrogen storage from solar power: Wang *et al.* (2024)** [17]	• Multi-scale modelling of hydrogen production from solid-oxide electrolysis (SOEC), with subsequent storage using a Mg–Ni hydride.	• Heat integration between hydrogen absorption and SOEC electrolyser feed improves overall efficiency by up to 10%.
	• Solar photovoltaic electricity used as energy input, with levelised cost of hydrogen calculated for different locations.	• Endothermic hydrogen desorption step relies on high-grade waste industrial heat.

		• Hydrogen productivity for each location estimated based on total available solar irradiation, without considering daily variation in supply.
**Model of hydrogen production from wind power: Kim *et al.* (2025)** [18]	• Seeks to develop a rule-based operating system to minimise the cost of hydrogen from wind power.	• The most cost-effective solution is to operate at a high battery level of ∼95%, with a small installed battery capacity that is easily charged and sporadically discharges.
	• Modelling considers changing PEM electrolyser efficiency as a function of electrical load, with supplemental batteries for electrical balancing.	• The true levelised cost of hydrogen is ∼6–20% higher than modelling which assumes perfect foresight for PEM following wind generation.
	• The rule-based system considers that PEM cannot react immediately to changing wind. A battery threshold charge level is used, above which supplemental electricity supports H_2 production.	
**Use of intermittent wind power for power-to-methanol *via* hydrogen: Fulham *et al.* (2024)** [19]	• Focuses on handling intermittency of wind power for methanol synthesis from direct air capture.	• The wind farm is the dominant capital expenditure for the process (∼50–80%).
	• The study considers isolated wind power, without imports of supplemental grid power.	• The dynamic operation framework ensures storage costs remain below 20% of the total capital expenditure.

	• Proposes dynamic adjustment of methanol production according to available wind power using multiple reactor modules in parallel, scaling the number of active reactors to power availability.	• Using reserve storage to achieve a wholly stable electricity supply to the process is prohibitively expensive.
		• Relying on grid power for stable electricity supply results in substantial net emissions of CO_2 from methanol production.
**Comparison of hydrogen and batteries for energy storage: Zhang *et al.* (2019)** [20]	• Compares power-to-hydrogen-to-power and lithium-ion batteries for balancing of wind power intermittency.	• If the market price of hydrogen exceeds €4.0/kg, a more profitable solution is to sell H_2 from electrolysis without fuel cells. Hydrogen demand is not considered.
		• Batteries are used to balance electrolyser load, avoiding shut-down costs, and to minimise 'lost wind'.
**Grid scale model of liquid air energy storage: Liang *et al.* (2025)** [21]	• Advocates the role of liquid air energy storage (LAES) for balancing renewable power in the UK electricity grid.	• In wind-dominated grid systems, LAES systems with charge/discharge times of ∼10–12 h are more suitable than the ∼4 h typical of batteries.
	• Modelling estimates the LCOE for various renewable penetrations and LAES capacity.	• LAES capacity should be ∼15–20% of installed renewables to provide balancing and minimise LCOE.
**Grid scale model of battery storage to cover short-term variation: Rayit *et al.* (2021)** [22]	• Grid-scale battery storage of curtailed wind energy, using one year of measured UK electricity supply and demand (June 2018–June 2019).	• Optimised system used 1.25 GWh of battery storage to supply the entire grid.

	• System relies on open and closed cycle gas turbines (OCGT and CCGT) and nuclear power for baseload; wind and batteries cover variable demand.	• Battery storage offset ∼34% of OCGT output, but only 0.5% of CCGT.
		• Around 20% of curtailed wind utilised.
		• Payback period of ∼14 years assuming static price of £75/MWh delivered electricity at current battery costs.
**Model of hydride storage in wind-based microgrids: Kumar *et al.* (2022)** [23]	• Optimisation study of a hybrid microgrid of wind and solar power in the UK or India, with $LaNi_5$ hydride storage of excess power.	• Using wind power alone increases required hydride storage by at least a factor of 2.
	• During unmet load periods, hydrogen is discharged from storage via fuel cells. Waste heat from PEM fuel cells assumed to drive endothermic hydride decomposition.	• Solely wind-powered microgrids yield more hydrogen for sale and marginally more exportable thermal energy.
		• Wind and solar hybrid microgrids give more stable output, fewer unmet loads, and lower storage needs.
		• Model assumes same daily load profile year-round, which limits realism.
**Use of hydride storage as an alternative method of hydrogen transport: Kotowicz *et al.* (2023)** [24]	• Focuses on metal hydride tanks for green hydrogen storage and transport vs. compressed/liquefied H_2.	• Metal hydride tanks offer 10× higher H_2 density and save ∼1.3 kWh/kgH_2 compared to compression.
	• Experimental work on lanthanum-cerium-nickel hydride plus techno-economic analysis.	• Levelised cost of hydrogen delivery estimated at \$6–7/kgH_2.

	• Thermal energy assumed stored alongside H_2 to enable endothermic release.	• 60–70% of hydrogen cost arises from electricity (assumed to be purchased externally).
**Model of hydrogen storage in wind and solar based power systems: Colbertaldo *et al.* (2019) [25]**	• Role of hydrogen as energy storage in 100% renewable systems with wind and solar.	• Switching from solar-dominated to wind-dominated RES increases required H_2 storage by 38%.
	• Model includes H_2 storage needs, electrolyser/fuel cell utilisation, and preliminary cost analysis.	• Power-to-H_2-to-power system costs ∼60% of combined wind/solar capacity installation for full RES.
**Model of hydride storage with wind-driven alkaline electrolysis: Pedrazzi *et al.* (2012) [26]**	• Wind-powered alkaline electrolysis + metal hydride storage + PEM fuel cells.	• Overall heat and power efficiency: 12.5%.
	• Balancing system analysed for domestic heat and power demands.	• Surplus H_2 remains available for sale at year-end.
		• Heat source for hydride decomposition not clearly specified.
**Gaseous hydrogen storage for balancing wind output for domestic heating: Samsatli and Samsatli (2019) [27]**	• System-wide cost analysis of using H_2 to balance wind intermittency for decarbonised UK heating.	• Around 80% of heating best provided directly via electricity rather than H_2 boilers.
	• Includes wind farm, H_2 generation/storage, boilers, pipelines.	• Remaining 20% H_2 contribution (with storage) essential for full heating decarbonisation.
	• H_2 stored in underground caverns.	• 40–60% of levelised heating cost from wind; ∼10% from H_2 storage/distribution.

Further findings from other studies, primarily on the use of renewable power for production of 'power-to-X' liquid fuels (*e.g* methanol, ammonia, synthetic aviation fuel) are described in the supplementary information of Ref. [19]. Other recent literature reviews relevant to this work discuss heat recovery and thermal management in solid-state H_2 storage [28–32], carbon capture using magnesium looping [33, 34], and thermo-chemical energy storage using carbonates [35].

3 Supplementary Note 3: Additional experimental information

S3.1 Synthesis and characterisation of H_2 storage material

The hydrogen storage alloy used in experiments was prepared by preliminary ball-milling of 90wt% Mg powder (Fisher Scientific, 99.8%, 325 mesh) and 10wt% Al powder (Fisher Scientific, 99.8wt%, 300 mesh) at 100 rpm for 2 h in a planetary ball-mill (Pulverisette 6, Fritsch) using zirconia balls with a 5:1 ball to sample mass ratio. The resulting powder was compressed into 1 cm diameter pellets under 2 tonnes pressure for 5 min, then sintered in a tube furnace at 550°C under Ar flow (BOC, 99.999%). The sintered pellet was crushed, and the resulting coarse powder mixed with 10wt% Ni (Fisher Scientific, 99.8%, 300 mesh) to catalyse hydrogen absorption [36], and 5wt% graphene nanoplatelets (Sigma Aldrich, 99%, <2 μm particle size) to aid heat transfer [3]. The resulting mixture was then ball-milled at 400 rpm for 5 h with a 20:1 ball to sample mass ratio, with milling paused for 15 min after every 15 min of milling. The milled material was then compressed into 0.6 g pellets, for use in hydrogen absorption experiments, with a final overall composition of 76.5wt% Mg, 8.5wt% Al, 10wt% Ni, 5wt% graphene.

In a typical measurement, a pellet of the alloy sample of known mass (0.82 g) was introduced into a stainless steel reactor vessel of known volume (25 cm^3) inside an Ar-filled glovebox, with a K-type thermocouple placed in contact with the pellet. The reactor was then sealed, and connected to a pipework system for delivery of pressurised hydrogen. The pressure inside the reactor was monitored using an electronic pressure transducer (Sinomeasure SIN-P300), to estimate the amount of H_2 absorbed or released over time. The system was purged of any residual air and moisture using He (BOC, 99.999vol%), then, the reactor was

heated to a set-point temperature using a tube furnace. Pressure-composition-temperature (PCT) isotherms, and cyclic hydrogen absorption/desorption measurements, were then collected as discussed in the main manuscript.

S3.2 Synthesis and characterisation of MgO-based TCES material

Microspheres of MgO were prepared using the method reported by Kondratowicz *et al.* [37]. Aqueous solutions (250 mL each) of 2 M $MgCl_2$ (Sigma-Aldrich, ≥98) and Na_2CO_3 were prepared, then preheated in an oven to 80°C. The Na_2CO_3 solution was then added to the $MgCl_2$ solution at 80°C. The resulting milky solution was aged for 2 h in the oven at 80°C, without stirring. The hot precipitate was then filtered, washed with deionised water (pH = 7), and dried under vacuum at 30°C overnight. The dry solid was then calcined for 3 h with heating rate of 2 °C min^{-1} in a muffle furnace in static air, to form the MgO product.

Nitrate catalyst was added to the surface of the MgO material by incipient wetness impregnation. Sodium nitrate (0.4 g, Thermo Scientific, 99+%) was dissolved in 1.3 mL deionised water (0.3 g_{salt} $mL_{H_2O}^{-1}$), and added dropwise with stirring to 1.6 g of MgO, until particles coalesced. The resulting material was then dried overnight at 120°C, and calcined at 450°C for 3 h in static air in a muffle furnace with a heating rate of 3°C min^{-1}, in order to form a final product of 20wt% $NaNO_3$ supported on MgO [38].

4 Supplementary Note 4: Model description and assumptions

A flowchart summarising the main processing steps for each simulated day is given in Fig. S2, with variation in energy demand for each season given in Table S3. In the event of a net deficit in energy supplied from the windfarm and from stored H_2, we calculated the required energy imports and CO_2 emissions from natural gas turbine usage. Similarly, in the event of a net surplus in energy generated from the windfarm that exceeded available energy storage capacity, the excess energy generation was curtailed.

Supplementary Table S3 Definitions of seasons as used by the UK National Grid [1], and variation in average seasonal demand relative to 5-year daily average [2] for the period 2016-2020. For the purposes of subsequent analyses, leap years were ignored, with data corresponding to electricity consumption or generation on the 29th February removed from all calculations.

Season	Date range (inclusive)	Date indices	Demand variation factor
Winter	1st Jan to 29th Mar, 26th Oct to 31st Dec	1-88, 299-365	1.11
Spring	30th Mar to 10th May	89-130	0.96
Summer	11th May to 19th July	131-200	0.90
High Summer	20th July to 1st Sept	201-244	0.88
Autumn	2nd Sept to 25th Oct	245-298	0.95

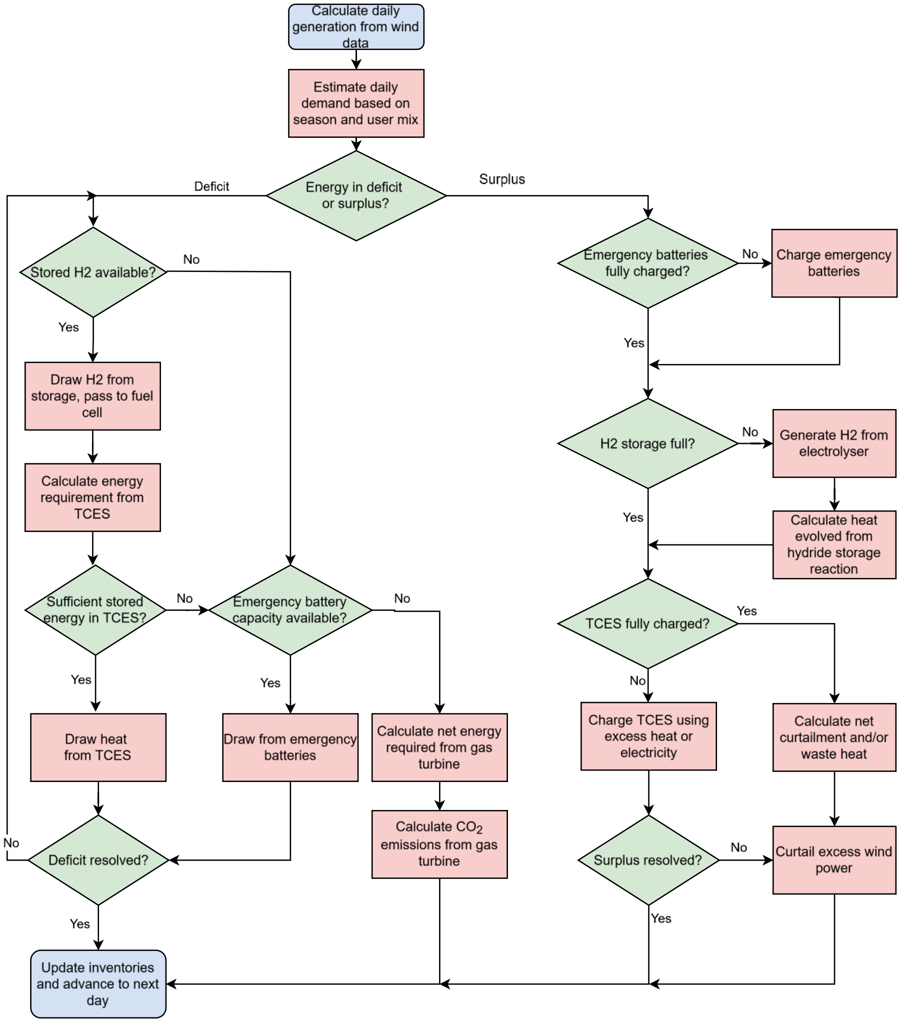

Supplementary Figure S2 Flowchart showing the processing loop for each simulated day. Energy consumption of compressors and electrical heating demand (including compensation for ambient heat loss) are included when estimating the amount of electricity available for H_2 production *via* electrolysis, or, the amount of hydrogen drawn from storage to fulfil demand.

S4.1 System operation

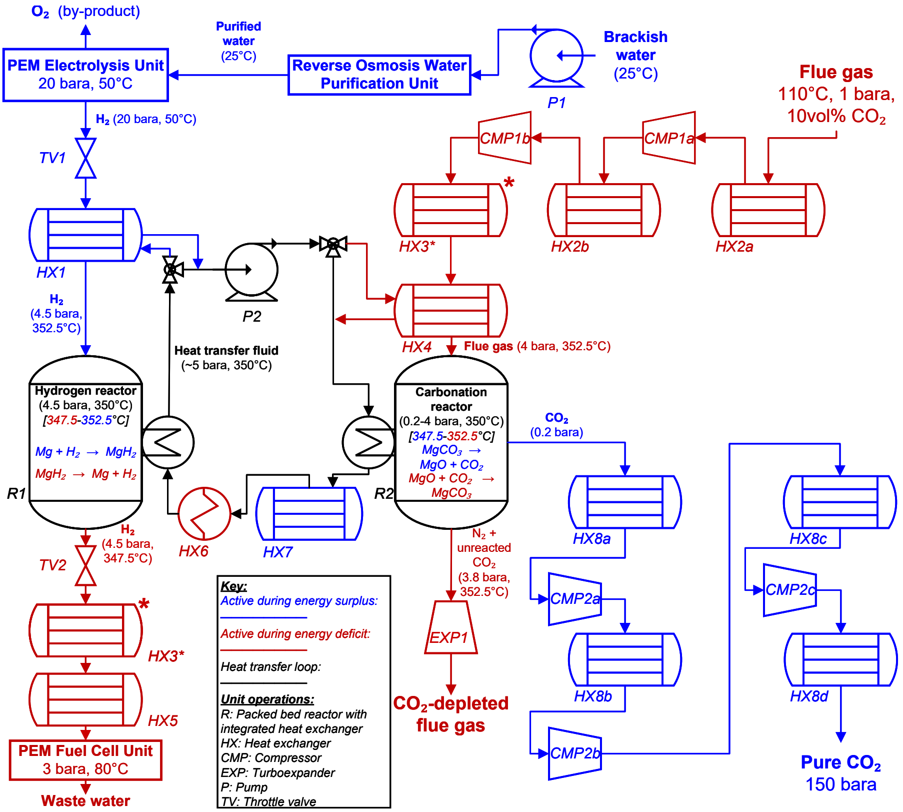

Supplementary Figure S3 Process flow diagram showing main unit operations and approximate stream conditions for the coupled hydrogen storage and carbonation reactors. Streams, reactions, and unit operations are highlighted in blue if operational during periods of net energy surplus, in red if operational during periods of net energy deficit, and in black if operational at all times, with example process conditions given for operation at a nominal reactor temperature of 350°C (* = indicates a single unit for heat integration between streams, shown twice for ease of interpretation). For certain reaction conditions (*i.e.* lower operating temperature, and hence lower outlet pCO_2), additional compressors CMP2d-e with intercooling were added to achieve 150 bara pipeline pressure.

For each modelled day, we estimated total electricity demand, and electricity supply from the windfarm. On days with a net energy surplus, brackish water was supplied to the reverse osmosis water purification unit. The purified water

was then transferred to the PEM electrolysis unit, generating H_2, which was then throttled to reactor pressure if required (neglecting any temperature change as a result of Joule-Thomson heating). Process conditions were selected to ensure that hydrogen storage reactor pressure did not exceed the electrolyser output, to avoid the need for pre-compression. In the hydrogen storage reactor, hydrogen reacted with the magnesium alloy to form magnesium hydride, with the reactor maintained at constant temperature by circulating heat-transfer fluid [39, 40] in a closed-loop system. The hydrogen feed to the reactor was pre-heated using some of the excess heat from the hydrogenation reaction. The remainder of the excess heat was transferred *via* the heat transfer fluid loop to the carbonation reactor, where magnesium carbonate was calcined to form magnesium oxide and CO_2. Any additional heat required was supplied by electrical heating in HX6, and any excess residual heat from the reaction was removed from the heat transfer fluid loop by external cooling in HX7.

The CO_2 liberated from the calcination reaction was then cooled and compressed in three stages to 150 bara for subsequent pipeline transportation and sequestration [41, 42]. In the optimistic scenario, the oxygen by-product generated from the electrolyser was assumed to be sold for use in a separate chemical process [19]; in the base and pessimistic scenarios, the oxygen was vented to the surroundings as a waste product. Any remaining excess power that could not be stored as H_2 as a result of insufficient storage capacity was curtailed.

On days with a net energy deficit, flue gas was supplied from an industrial point source, modelled as dry gas with 10vol% CO_2, balance N_2, at 110°C, 1 bara [43], compressed to reactor pressure with intercooling. The inlet pressure of the flue gas to the reactor, P_{inlet} (bar) was determined using Eq. S1 where y_{CO_2} is the molar fraction of CO_2 in the flue gas.

$$P_{inlet} = \frac{p_{CO_2,eq}}{y_{CO_2}(1 - X_{CO_2})} \tag{S1}$$

For calculations reported here, a CO_2 capture efficiency of 50 mol% was assumed, comparable with reported values in packed-bed experiments [44], with a flue gas feed of 10 vol% CO_2 at a reactor temperature of 350°C (with $p_{CO_2,eq} = 0.2$ bara), giving $P_{inlet} = 4$ bara.

The flue gas was pre-heated to 350°C, and fed to the carbonation reactor, where the CO_2 reacted with the calcined magnesium oxide to form magnesium carbonate, releasing heat. The remaining N_2 and CO_2 from the flue gas leaving the reactor was expanded to 1 bara to recover some of the compression work. The excess heat generated in the carbonation reactor was removed by the heat transfer fluid loop, which was then used to pre-heat the flue gas feed, and to provide heat to the hydrogen storage reactor, where the magnesium hydride decomposed to form magnesium metal and hydrogen gas. Any supplementary heat required to decompose the magnesium hydride was supplied to the heat transfer fluid loop by electrical heating. The liberated hydrogen was throttled to 3 bara and cooled to 80°C, then supplied to the PEM fuel cell stack, generating electricity and waste water.

For the purposes of modelling, both reactors were assumed to be well-mixed and to operate isothermally, with the operating temperature set by the temperature of fluid in the heat transfer loop.

The pseudo-isothermal reactor behaviour considered here differs from TCES reactor systems designed for stand-alone electricity storage [45, 46], where the difference in gas temperature between the inlet and exhaust of a packed bed of reactive solids is used to store or release energy. Rather, by continuously removing heat from the bed by means of internal heat transfer coils, the system approximates to a well-mixed reactor with an isothermal reaction front [47]. Furthermore, balanced heat transfer between reactors as modelled here would only be achievable by controlling both reactions such that the ratio of rates of reaction was exactly equal to the ratio of reaction enthalpies, in order to avoid either reaction becoming limited by heat transfer. However, by using a fluid loop to transfer heat, rather than directly coupling the reactions in a single

vessel [30, 48], the heat capacity of the heat transfer fluid could be employed as a thermal buffer, with an additional buffer tank incorporated if required to increase the overall thermal mass of the fluid loop.

For the TCES system to operate, there must be a temperature driving force for heat transfer from the TCES reactor to or from the hydrogen storage reactor, and hence, the exothermic step (hydrogen storage or TCES carbonation) must operate at a higher temperature than the endothermic step (hydrogen discharging or TCES calcination). Over the thermodynamic overlap range shown in Fig 1 (335-415°C) where both reactions are feasible, selection of temperature, and temperature difference between the reactors, is arbitrary, and for any selected temperature, will not be optimal for either reaction. However, the efficiency of hydrogen storage and release, and the resulting round-trip efficiency, depend on both the energy required to pre-heat the H_2 and CO_2 feeds and reactor temperature, and the compression duties as shown in Fig. S5. Therefore, altering the assumed reactor operating temperature, temperature difference between reactors, CO_2 feed fraction, and *per*-pass CO_2 conversion, affect the overall round trip efficiency, shown in Fig. S4, with optimal overall efficiency at a nominal operating temperature of 350°C.

Given the relatively low energy efficiency of the electrolyser, the value of ξ_{in} is insensitive to the reactor conditions. However, ξ_{out} shows a maximum with increasing reactor temperature or CO_2 conversion, at around 350°C and 50 vol% CO_2 conversion. As temperature or conversion increases, reactor operating pressure increases, however the volume of gas passing through the compressor decreases, resulting in a local maximum. The value of ξ_{out} was however relatively insensitive to the temperature driving force for heat transfer between reactors. With a greater temperature difference during dehydrogenation (at a nominal average operating temperature of 350°C), the temperature and pressure of the carbonation reaction increased, but the increase in compressor work to achieve the reactor pressure was compensated by energy recovery in the turbo-expander. More complex behaviour was

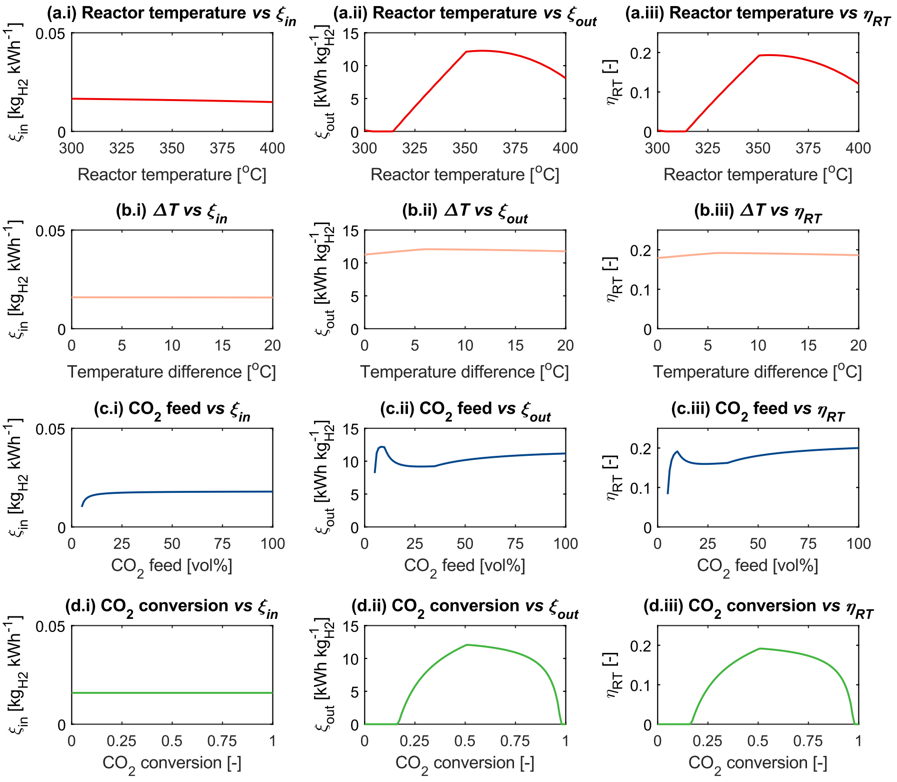

Supplementary Figure S4 Efficiencies of hydrogen storage and release, and round-trip efficiency as a function of **(a)** average reactor temperature (T), **(b)** temperature difference between reactors (ΔT), **(c)** flue gas composition (Y), and **(d)** CO_2 capture efficiency (X). For each case, the other parameters were fixed, at baseline values of $T = 350°\text{C}$, $\Delta T = 5°\text{C}$, $Y = 10\text{vol\%}$, $X = 0.5$.

observed with increasing CO_2 feed concentration, as a result of the interplay between flue gas heat capacity and compression duties (with unphysical values at very low CO_2 feed concentrations omitted). However, assuming thermodynamic limitation within the reactor, the overall effect on ξ_{out} and η_{RT} is limited, with the representative value of 10 vol% feed close to a local maximum.

Estimation of ambient heat losses

In the base and pessimistic modelling scenarios, both reactor vessels were assumed to lose heat to the surroundings, with the rate of heat loss estimated using correlations for natural convection from a horizontally-oriented cylinder surrounded by stagnant air [49]. In the base scenario, the vessels were assumed to be wrapped with insulating material, with an overall heat transfer coefficient of 0.7 W $m^{-2}K^{-1}$ between the surface of the vessel and the surface of the insulation [50], and in the pessimistic scenario, the vessels were assumed to have no insulation, with the surface temperature equal to the average bulk temperature of the reactor contents (*i.e.* 350°C). In the optimistic scenario, the reactor vessels were assumed to be perfectly insulated, with no heat losses to the surroundings. In order to maintain a constant reactor temperature, additional heat to compensate for ambient losses was supplied *via* the heat transfer fluid loop, using the electrical heater HX6 to generate the additional heat required. While lower thermal losses could potentially be achieved by allowing the reactors to cool to ambient temperature during long periods of no hydrogen release or storage, the decision to shut-down or re-activate the system would require daily prediction of future weather conditions, and so was deemed beyond the scope of this study. Allowing the system to cool to ambient conditions in between charging and discharging steps could potentially improve efficiency by decreasing the amount of additional heating required to compensate for ambient losses, at the cost of requiring a larger capacity heater to start up the reactors from cold in response to a surplus or deficit in windfarm output. Moreover, for the simulated systems, the longest periods of reactor inactivity in the 5-year modelling period were 27 and 16 days for on-shore or off-shore windfarms respectively (as shown in Tables S8 and S9), and therefore shutting down the system was not considered worthwhile.

Material lifetime and replacement

The Mg-based alloy was assumed to have an approximate lifetime of 3,000 H_2 absorption-desorption cycles in the base-case scenario, corresponding to around 16 years of operation [4]. The cost of material makeup was therefore estimated at 1/16 of capital cost *per* year, not including any scrap value or recycling costs, corresponding to around 14% of operating expenditure as shown in Fig. 7.

Given the relatively low cost of the MgO-based CO_2 sorbents used for TCES (<1% total capital expenditure for all modelled cases), degradation over time was not modelled explicitly. However, some MgO-based sorbents are vulnerable to sintering, resulting in a loss of CO_2 capacity over repeated carbonation and calcination cycles [38, 51]. Therefore, for practical operation, the MgO-based sorbent would likely need to be replaced or regenerated periodically, in order to maintain system performance, but this was not expected to have a significant effect on total operating expenditure.

S4.2 Polymer electrolyte membrane electrolyser and fuel cell

A polymer electrolyte membrane (PEM) electrolyser and fuel cell were selected for converting electricity to H_2 and *vice-versa*, given the rapid start-up of PEM stacks relative to *e.g.* alkaline or solid-oxide electrolysers [52], allowing for scaled capacity relative to the magnitude of the energy surplus or deficit on any given day. Nevertheless, rapid on-off cycling of PEM electrolysers or fuel cells is detrimental to operational lifetime [53–55], and therefore, rather than directly coupling the electrolyser or fuel cell to wind turbine output, a lithium-ion battery system was employed as a buffer [20, 54, 56], allowing net H_2 production or consumption to be spread out over each 24 h period.

The electrolyser was assumed to convert H_2O to H_2 and O_2 at 20 bara and 50°C [57] and the PEM fuel cell was assumed to operate at 80°C and 3 bara [58]. As the operating temperatures of the PEM units were well below the

temperatures of the reactors, the electrolyser and fuel cell were not heat-integrated with other units, in contrast with other studies modelling solid-oxide electrolysis [17], where waste heat from hydrogen storage was used to pre-heat the electrolyser feed.

As the heat produced in the fuel cell stack was of relatively low grade, heat integration of the fuel cell with other unit operations was not considered worthwhile.

The electrical efficiencies of each PEM unit, ξ_{elec} (kg_{H_2} kWh^{-1}) and ξ_{fc} (kWh $kg_{H_2}^{-1}$), were assumed to remain constant over the equipment lifetime, with the assumption that given the modular construction of PEM stacks [59], degraded sections could be replaced *ad hoc* to maintain near-constant output. Hence, the cost of stack replacement was amortised over the average stack lifetime, estimated at approximately 6 years in the base-case scenario.

S4.3 Hydrogen storage and carbonation reactors

The capacity of solid H_2 storage, and hence the mass of Mg alloy required, was determined using Eq. S2, where m_{alloy} is the mass of hydrogen storage alloy (kg), n is the equivalent number of days of energy demand stored as H_2 (-), L is the daily electricity demand (kWh d^{-1}), ξ_{fc} is the electrical efficiency of the fuel cell (kWh $kg_{H_2}^{-1}$), and w_{H_2} is the gravimetric H_2 capacity of the hydrogen storage material (kg_{H_2} kg_{alloy}^{-1}).

$$m_{alloy} = n\frac{L(1 - w_{H_2})}{\xi_{fc} w_{H_2}} \tag{S2}$$

The mass of TCES material was then determined using Eq. S3, where m_{TCES} is the mass of fully discharged TCES material (*i.e.* here, $MgCO_3$), ξ_{HT} is the efficiency of heat transfer between the two reactors (-), ΔH_{H_2} and ΔH_{CO_2} are the enthalpy changes for the reactions $MgH_2 \rightarrow Mg + H_2$ and $MgCO_3 \rightarrow MgO + CO_2$ respectively (kWh $kg_{H_2}^{-1}$ and kWh $kg_{CO_2}^{-1}$), $M_r(i)$ is the molar mass of species i (kg $kmol^{-1}$) and X_{TCES} is the assumed conversion of the MgO material during carbonation (-).

$$m_{TCES} = \frac{1}{\xi_{HT} X_{TCES}} \frac{\Delta H_{H_2} m_{alloy} \left(\frac{w_{H_2}}{1 - w_{H_2}} \right)}{\Delta H_{CO_2} \left(\frac{M_r(CO_2)}{M_r(MgCO_3)} \right)} \tag{S3}$$

The residence time of stored hydrogen was estimated using Eq. S4, where τ is the average residence time (d), n_{tot} is the total number of days in the modelling period (here, 1825 for 5 years of input data), $m_{H_2,i}$ is the mass of hydrogen withdrawn from storage on day i (kg_{H_2}), and $m_{alloy} w_{H_2}$ is the total hydrogen storage capacity of the system (kg_{H_2}).

$$\tau = n_{tot} \frac{m_{alloy} w_{H_2}}{\sum_{i=1}^{n_{tot}} m_{H_2,i}} \tag{S4}$$

The length, l_{vessel}, of reactors R1 and R2 was estimated by calculating the volume of the least dense material contained within the reactor (*i.e.* MgH_2 or $MgCO_3$) using Eq. S5, where m and ρ correspond to the mass (kg) and density (kg m^{-3}) of the material respectively, assuming a bed voidage, ε, of 0.4, and a cylindrical reactor profile with a diameter, D, of 3 m.

$$l_{vessel} = \frac{4m}{\pi D^2 \rho (1 - \varepsilon)} \tag{S5}$$

The vessel for reactor R1 was assumed to be constructed from austenitic stainless steel in order to mitigate hydrogen embrittlement [60]; the vessel for reactor R2 was assumed to be constructed from carbon steel [61].

Hydrogen absorption was assumed to be limited by the equilibrium partial pressure of the hydride formation reaction and the experimentally-derived hydrogen capacity of the Mg-based materials, assuming that over the 24 hour modelling period, the system was able to approach thermodynamic equilibrium [62], with some gaseous hydrogen remaining in the reactor headspace in order to maintain equilibrium. Hence, the round-trip efficiency parameters estimated here represent an upper-bound estimate for the case where thermodynamic equilibrium is achieved; if excess hydrogen generation were required to compensate for incomplete absorption, the effective round-trip efficiency would be decreased. In practice, to achieve an additional driving force for absorption, the hydrogenation reaction could be operated above equilibrium pressure by

using the electrolyser output directly (*i.e.* removing pressure regulation valve TV1).

S4.4 Backup gas turbines

Taking the lower heating value (LHV) of natural gas to be 12.9 kWh kg^{-1} [63], the approximate carbon intensity, CI_{GT} (kg$_{CO_2}$ kWh^{-1}), of electricity generated from backup gas turbines was estimated using Eq. S6, where η_{GT} is the electrical efficiency of the power plant (kWh$_{electricity}$ kWh$^{-1}_{fuel}$), and $M_r(i)$ is the molar mass of species i (g mol^{-1}), taking the average molar mass of natural gas to be approximately 19 g mol^{-1}.

$$\mathrm{CI}_{GT} = \frac{M_r(CO_2)}{M_r(NG) \cdot \eta_{GT} \cdot \mathrm{LHV}_{NG}} \tag{S6}$$

S4.5 Heat exchange equipment

All heat exchangers were of counter-current shell and tube design, assuming a constant heat transfer coefficient, U (kW m^{-2}K^{-1}), between the two fluids exchanging heat, tabulated in Table S4, with the exception of the resistive electric heater HX6, which was assumed to operate with 100% thermal efficiency. The heat transfer area, A_{HX} (m^2) for each unit was estimated using Eq. S7, where $\dot{Q}_{max}$ is the maximum rate of heat transfer (kWh d^{-1}) through the unit over the five-year modelling period, and ΔT_{LMTD} is the log mean temperature difference (°C) between the streams entering and exiting the exchanger.

$$A_{HX} = \frac{\dot{Q}_{max}}{U \Delta T_{LMTD}} \tag{S7}$$

The fluid properties of Dowtherm A were used for the heat transfer oil [64] (with a maximum stable operating temperature of 400°C). Heat exchangers HX1, HX4, HX6, and HX7 were connected in a heat transfer loop to transfer heat between unit operations using the heat transfer oil.

During hydrogen storage, the hydrogen leaving the fuel cell at *c.* 50°C is heated to 352.5°C in HX1, with heat supplied from the heat transfer loop, before being fed to reactor R1. In reactor R1, the heat evolved from the exothermic hydrogenation reaction is transferred to the heat transfer loop using HXR1. Heat is then transferred from the heat transfer loop to drive the endothermic calcination reaction in reactor R2, *via* the heat transfer coil HXR2. In the event of a net surplus of heat evolved from the hydrogenation reaction relative to the heat used to pre-heat the hydrogen feed and drive calcination, any excess heat is removed in cooler HX7. Then, the pure CO_2 released from R2 in the calcination reaction is intercooled to 25°C in HX8a-8d in between compression stages prior to pipeline injection at 150 bara. During hydrogen release, heat is supplied to drive the endothermic dehydrogenation reaction from the heat transfer loop in HXR1. The flue gas feed to R2 is intercooled to 25°C between compression stages in HX2a and HX2b. Then, the feed is pre-heated in HX3 and HX4, with HX3 transferring heat from the hot hydrogen outlet at 347.5°C leaving R1, and HX4 supplying heat from the heat transfer loop. The reaction heat from the exothermic carbonation reaction in R2 is transferred to the heat transfer loop in HXR2. The hydrogen leaving HX3 is further cooled to 80°C in HX5 before being transferred to the fuel cell, with no heat recovered from the outlet waste water. In the event of a net deficit in heat generated from the carbonation reaction relative to the heat required to pre-heat the reactor feed and drive the dehydrogenation reaction, additional supplementary heat is supplied to the heat transfer loop using electric heater HX6.

S4.6 Compressors and turbo-expanders

The specific work, $w_{compress}$ (kWh kg^{-1}) required to compress the flue gas feed to reactor R2 in compressors CMP1a-b, and to compress the CO_2 product for sequestration in compressors CMP2a-c, was estimated using Eq. S8, where P_1 and P_2 correspond to the inlet and exit pressure (bara) respectively, C_p and γ correspond to the specific heat capacity (kWh kg^{-1}K^{-1}) and heat capacity

Supplementary Table S4 Summary of heat exchange equipment and fluids exchanging heat, with a brief description of the purpose of each exchanger in the process, and estimated overall heat transfer coefficients from Ref. [61]. Equipment codes correspond to items of process equipment shown in Fig. S3. *=Hot and cold fluids swap over depending on whether daily energy supply is in net surplus or deficit, with the energy deficit case given here.

Code	Purpose	Hot Fluid	Cold Fluid	Heat transfer coefficient ($kW\ m^{-2}K^{-1}$)
HX1	Pre-heater for feed to hydrogen storage reactor	Heat transfer oil	Hydrogen	0.1
HX2a-b	Intercooling between R2 feed compression stages	Flue gas	Cooling water	0.2
HX3	Heat integration between hydrogen outlet and carbonation reactor feed	Hydrogen	Flue gas	0.05
HX4	Additional pre-heating for carbonation reactor feed	Heat transfer oil	Flue gas	0.1
HX5	Cooling hydrogen feed to fuel cell	Hydrogen	Cooling water	0.1
HX6	Supplementary electrical heating to resolve net reaction heat deficits	N/a (Electric heater)	Heat transfer oil	N/a
HX7	Supplementary cooling water to resolve net reaction heat surpluses	Heat transfer oil	Cooling water	0.15
HX8a-d	Intercooling between CO_2 compression stages	CO_2	Cooling water	0.2
HXR1	Heat transfer coil in R1 to supply/remove heat from (de)hydrogenation reaction	Heat transfer oil*	Hydrogen*	0.1
HXR2	Heat transfer coil in R2 to supply/remove heat from (de)carbonation reaction	CO_2*	Heat transfer oil*	0.1

ratio (-) respectively of the gas being compressed (estimated at inlet conditions using values from the NIST Webbook [65]), T_1 corresponds to the inlet temperature (K), and $\eta_{isentropic}$ corresponds to a lumped isentropic efficiency parameter for the compressor (estimated at 0.85 for the base scenario) [66]. The maximum compression ratio ($\frac{P_2}{P_1}$) was determined using Eq. S9 by assuming a constant maximum compressor exit temperature, T_2 (K), in the range 473-503 K (200-230°C) for compression of CO_2 and N_2, or 423 K (150°C) for compression of H_2 [67]. Under base case assumptions ($\eta_{isentropic} = 0.85$, $T_2 = 473$ K), for flue gas compression from ambient pressure to 4 bara in CMP1a and CMP1b, pressure ratios of up to 4 were used in each compressor in series. For CO_2 compression from the reactor R2 outlet to pipeline injection at 150 bara in CMP2a, CMP2b, and CMP2c, overall pressure ratios of 5.8 were used for each of the three compressors in series, corresponding to centrifugal compressors with multiple impellers and sequential compression stages *per* unit [68].

$$w_{compress} = \frac{1}{\eta_{isentropic}} C_p T_1 \left(\left(\frac{P_2}{P_1} \right)^{1-\frac{1}{\gamma}} - 1 \right) \quad \text{(S8)}$$

$$\frac{P_2}{P_1} = \left(\left(\frac{T_2}{T_1} - 1 \right) \cdot \eta_{isentropic} + 1 \right)^{\frac{\gamma}{\gamma-1}} \quad \text{(S9)}$$

The specific work recovered in turbo-expander EXP1, w_{expand} (kWh kg^{-1}), was estimated using Eq. S10, assuming an exit pressure of 1 bara. Energy losses during conversion from mechanical work to electricity were assumed to be negligible.

$$w_{expand} = \eta_{isentropic} C_p T_1 \left(\left(\frac{P_2}{P_1} \right)^{1-\frac{1}{\gamma}} - 1 \right) \quad \text{(S10)}$$

The total work required to compress the flue gas feed to the carbonation reactor R2 was affected by the reactor operating temperature, as shown in Fig. S5a, with the total operating pressure determined by the equilibrium pCO_2 of the carbonation reaction, the concentration of CO_2 in the flue gas feed, and the *per*-pass conversion of CO_2 during carbon capture.

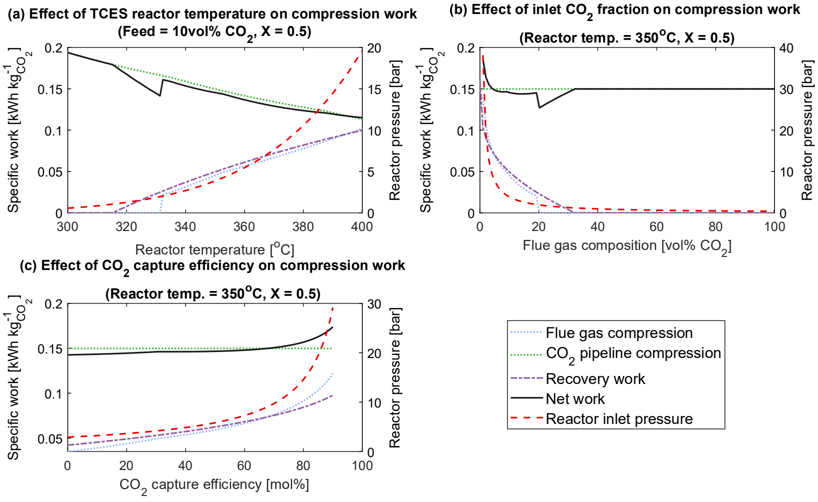

Supplementary Figure S5 Compression and expansion work as a function of **(a)** carbonation reactor (R2) temperature at fixed flue gas composition (10vol% CO_2) and CO_2 capture efficiency ($X = 0.5$), **(b)** flue gas composition at fixed reactor temperature (350°C) and CO_2 capture efficiency ($X = 0.5$), and **(c)** CO_2 capture efficiency at fixed reactor temperature (350°C) and flue gas composition (10vol% CO_2).

At higher reactor temperatures (up to 400°C, the maximum working temperature of Dowtherm A heat transfer fluid), net work required *per* unit

mass of CO_2 passing through the system decreased, primarily as a result of higher equilibrium pCO_2 during calcination decreasing the work required to compress the outlet CO_2 to 150 bar for pipeline transportation. The work recovered in EXP1 was also a function of reactor temperature, as the flue gas leaving CMP1b at 230°C was then heated in HX3, HX4 and R2 to 350°C, and subsequently expanded to 1 bar in EXP1, thereby converting heat to work *via* the Brayton cycle.

The total duty of compressors and expanders in the system was also determined by the composition of the flue gas feed, y_{CO_2}, as shown in Fig. S5b, and the capture efficiency of the carbonation reaction $MgO + CO_2 \rightarrow MgCO_3$, shown in Fig. S5c. At very low flue gas concentrations or very high capture efficiency, theoretical reactor pressure required from Eq. S1 diverged to infeasibly high values, in order to maintain the condition of the exhaust gas from the reactor leaving in equilibrium with the MgO-$MgCO_3$ solids. For a modified process with a highly concentrated CO_2 feed or higher capture efficiency (*e.g.* operating on a 'closed loop' with compressed CO_2 storage, or using the output from a direct-air CO_2 capture system as a feed [69]), compression duties could be decreased by operating below equilibrium pressure, at the cost of some thermodynamic efficiency.

S4.7 Battery subsystem

A lithium-ion battery system was included as part of the process, in order to balance short-term fluctuations in power supply and energy demand (shorter than the modelling time step of 24 h), allowing the power input and output of the PEM electrolyser and fuel cell respectively to be averaged over each full day [20, 70]. The energy capacity of the batteries was scaled to equal 1/6 of average daily energy demand *i.e.* up to 4 h of continuous discharging, the maximum discharge time of a typical commercial grid-scale Li-ion battery unit [71].

As battery storage levels were not modelled explicitly, a simplified assessment was performed to determine whether 4 h of short-term storage capacity (*i.e.* the maximum discharge time of a commercial grid-scale Li-ion battery unit) would be sufficient to smooth hourly variation in wind turbine output over 24 h, such that daily wind turbine output could be considered as a single parameter. For each day in the dataset for the onshore and offshore locations, the maximum cumulative deviation in wind output from the daily average was estimated (shown for an example day in Fig. S6a). Then, normalising the cumulative deviation with respect to average windfarm output, and scaling according to f_{OCP} as defined in the main manuscript, the minimum number of hours of battery capacity to smooth windfarm output was estimated for each day, shown in Fig. S6b), giving an average necessary capacity of 2.3 $\pm$ 2.0 h and 2.7 $\pm$ 1.9 h for offshore and onshore windfarms respectively.

However, as shown by the tail of longer capacities , some days with unusual weather patterns might require greater short-term storage capacity for 'true' smooth operation, requiring access to either external grid battery storage or additional energy imports from gas turbines. However, on days with a net energy deficit from the windfarm, energy would also be entering the system throughout the day from use of stored hydrogen in the fuel cell, partially counterbalancing short-term deficits in available wind power (and *vice versa* on days with a net energy surplus, with excess power used to generate hydrogen in the electrolyser), thereby decreasing the minimum necessary short-term storage capacity. Hence, for the purposes of this study, the interplay and scheduling requirements between short- and long-term storage (batteries and hydrogen respectively) was not modelled. Therefore, 4 h battery capacity was deemed sufficient for rough cost estimation, while noting that greater battery capacities might be required for sites with rapid hourly fluctuations in capacity factor.

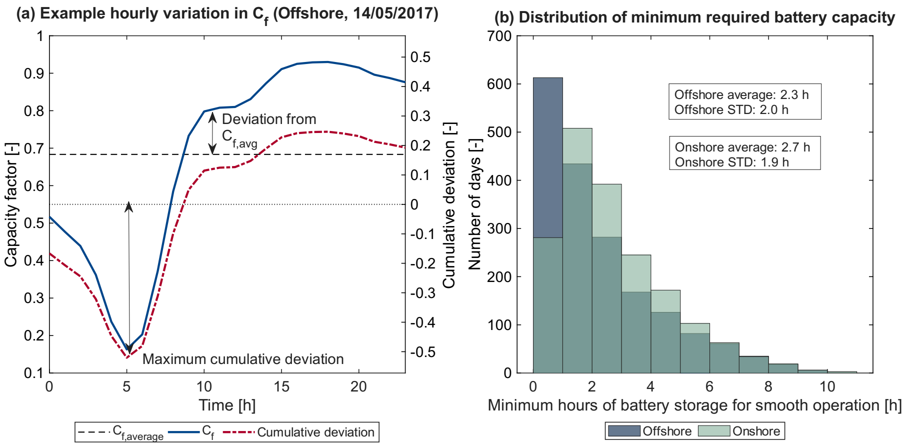

Supplementary Figure S6 (a) Hourly variation in wind turbine capacity factor, and cumulative deviation from daily average capacity factor for a representative day (offshore North Sea wind farm, 14/05/2017), (b) histogram of estimated minimum battery capacity for each day in the dataset for onshore and offshore windfarms.

S4.8 Derivation of expressions for round-trip efficiency

The round-trip efficiency (η_{RT}) of the H_2 storage system is given by the product of the net electrical energy required *per* unit mass of hydrogen produced by electrolysis and introduced to storage (ξ_{in}, kg_{H_2} kWh^{-1}), and the net electrical energy generated *per* unit mass of hydrogen removed from storage and consumed in the fuel cell (ξ_{out}, kWh $kg_{H_2}^{-1}$).

During hydrogen release, a certain mass of hydrogen, m_{H_2} (kg) is formed by decomposition of the metal hydride. In order to generate sufficient heat to release the H_2, a certain mass of CO_2 from flue gas, m_{CO_2} (kg), reacts with MgO to form $MgCO_3$. The total useful electricity generation (E_{tot}, kWh) is given by Eq. S11, where E_{FC} is the electricity generated in the fuel cell, $E_{compress}$ is the energy expended compressing the flue gas to reactor pressure, and E_{heat} is any supplementary electrical heating required to bring the compressed flue gas to the reactor temperature.

$$E_{tot} = E_{FC} - E_{compress} + E_{expand} - E_{heat} \tag{S11}$$

The terms E_{FC}, $E_{compress}$ and E_{expand} are given by Eqs. S12, S13, S14 respectively, where ξ_{fc} is the electrical efficiency of the fuel cell (kWh $kg_{H_2}^{-1}$), w_i corresponds to the compression or expansion work *per* kg of gas, X_{CO_2} is the *per*-pass conversion of CO_2 in the carbonation reactor, and Y_{CO_2} is the mass fraction of CO_2 in the flue gas feed.

$$E_{FC} = m_{H_2}\xi_{fc} \tag{S12}$$

$$E_{compress} = \frac{m_{CO_2} w_{compress}}{X_{CO_2} Y_{CO_2}} \tag{S13}$$

$$E_{expand} = \frac{m_{CO_2}(1 - X_{CO_2} Y_{CO_2}) w_{expand}}{X_{CO_2} Y_{CO_2}} \tag{S14}$$

From an overall energy balance over the system, including the heat required to heat the flue gas to reactor temperature, q_{PH,CO_2} (kWh $kg_{CO_2}^{-1}$, given by Eq. S15 where $C_{p,flue}$ is the specific heat capacity of the flue gas including the inert N_2 ballast), and the heat recovery from the released hydrogen, q_{cool} (kWh $kg_{H_2}^{-1}$), the value of E_{heat} is given by Eq. S16.

$$q_{PH,CO_2} = \frac{m_{CO_2} C_{p,flue}(T_{reactor} - T_{compressorexit})}{X_{CO_2}} \tag{S15}$$

$$0 = -m_{H_2}\Delta H_{H_2} + m_{CO_2}\Delta H_{CO_2} + m_{H_2} q_{cool} - m_{CO_2} q_{PH,CO_2} + E_{heat} \tag{S16}$$

For a given mass of hydrogen discharged, the system as defined so far is under-constrained, as the heat requirement for the endothermic hydride decomposition reaction can be satisfied either by expending electricity to generate heat, or by feeding a greater mass of CO_2 to generate heat *via* the MgO carbonation reaction. However, given hydrogen discharge occurs during periods of insufficient electricity generation, the overall efficiency is maximised by setting $E_{heat} = 0$ during hydrogen discharge. Therefore, the mass of CO_2 feed required is given by Eq. S17.

$$m_{CO_2} = m_{H_2} \frac{\Delta H_{H_2} - q_{cool}}{\Delta H_{CO_2} - q_{PH,CO_2}} \tag{S17}$$

Substituting into Eq. S11 and rearranging, the value of ξ_{out} is given by Eq. S18, as in the main text.

$$\xi_{out} = \frac{E_{tot}}{m_{H_2}} = \xi_{fc} - \frac{\Delta H_{H_2} - q_{cool}}{\Delta H_{CO_2} - q_{PH,CO_2}} \left(\frac{w_{compress} - (1 - X_{CO_2} Y_{CO_2}) w_{expand}}{X_{CO_2} Y_{CO_2}} \right) \tag{S18}$$

During H_2 charging, surplus electricity is used to produce hydrogen by electrolysis, with the heat of reaction used to calcine $MgCO_3$ to form CO_2, which is subsequently compressed to 150 bar for pipeline transportation. The total electricity required to generate and store H_2 is given by Eq. S19, where E_{elec} corresponds to electricity consumed in the electrolyser (given in Eq. S20) $E_{compress}$ is the compression work required to bring the CO_2 to pipeline pressure (given in Eq. S21), and E_{heat} is any supplementary electrical heating applied to calcine $MgCO_3$ to form CO_2.

$$E_{tot} = E_{elec} + E_{compress} + E_{heat} \tag{S19}$$

$$E_{elec} = \frac{m_{H_2}}{\xi_{elec}} \tag{S20}$$

$$E_{compress} = m_{CO_2} w_{pipeline} \tag{S21}$$

As previously, from an overall heat balance, E_{heat} is given by Eq. S22, where in this case q_{PH,H_2} corresponds to the heat required to heat the hydrogen leaving the electrolyser to reactor temperature.

$$E_{heat} = m_{CO_2} \Delta H_{CO_2} - m_{H_2} (\Delta H_{H_2} - q_{PH,H_2}) \tag{S22}$$

However, in order to achieve an overall mass balance between carbonation and decarbonation steps (*i.e.* maintaining a fixed ratio of uptake and release of H_2 and CO_2), the mass of CO_2 leaving the reactor is set by Eq. S17, yielding a non-zero value of E_{heat} during hydrogen storage, given by Eq. S23.

$$E_{heat} = m_{H_2}\left(\left(\frac{\Delta H_{H_2} - q_{cool}}{\Delta H_{CO_2} - q_{PH,CO_2}}\right)\Delta H_{CO_2} - (\Delta H_{H_2} - q_{PH,H_2})\right) \quad \text{(S23)}$$

Therefore, by substituting into Eq. S19 and rearranging, ξ_{in} is given by Eq. S24, as in the main text.

$$\xi_{in} = \frac{m_{H_2}}{E_{tot}} = \frac{\xi_{elec}}{1 + \xi_{elec}\left(\frac{\Delta H_{H_2} - q_{cool}}{\Delta H_{CO_2} - q_{PH,CO_2}}(w_{pipeline} + \Delta H_{CO_2}) - (\Delta H_{H_2} - q_{PH,H_2})\right)} \quad \text{(S24)}$$

Under base case modelling assumptions, the overall values of ξ_{in} and ξ_{out} are 0.0159 kg_{H_2} kWh^{-1} and 11.3 kWh $\text{kg}_{H_2}^{-1}$ respectively, giving $\eta_{RT} = 0.18$. As noted in the main manuscript, the decision to set $E_{heat} = 0$ during H_2 discharging is arbitrary, but justifiable from an energy efficiency perspective. For example, if the system were adjusted such that during hydrogen release, the energy required to preheat the flue gas feed were supplied solely from electrical heating (*i.e.* setting $m_{CO_2}\Delta H_{CO_2} = m_{H_2}\Delta H_{H_2}$ in order to balance the heats of reaction, where m_i is the mass stored of component i and ΔH_i is the enthalphy of reaction for dehydrogenation or carbonation), in effect 'shifting' some of the net heating duty from the hydrogen charging to the discharging step, round-trip efficiency decreases to around 0.15. Alternatively, in the case with no carbonate TCES, $m_{CO_2} = 0$, and all heating duty is provided by electrical heating, giving $\eta_{RT} = 0.038$.

S4.9 Configurations without heat storage

As discussed in the main manuscript, the round-trip efficiency for a system with no heat storage was estimated by setting the mass of TCES material to zero, and leaving the remainder of the process model unchanged, in order to ensure a fair comparison between systems. Therefore, during H_2 release, heat was supplied to reactor R1 using electrical heater HX6, giving $\eta_{RT,noheatstorage} = \xi_{elec} \cdot (\xi_{fc} - \Delta H_{H_2}) = 0.038$: in effect, converting stored hydrogen to electricity in the fuel cell, then using the electricity to generate heat in order to release more H_2. However, given the poor exergy efficiency of converting hydrogen to electricity, then electricity to heat, the overall efficiency of a purpose-built system without heat storage could be improved by combusting a fraction of the hydrogen released to generate heat equal to the reaction enthalpy ΔH_{H_2} for the reaction $MgH_2 \rightarrow Mg + H_2$. The overall energy efficiency of hydrogen conversion to useful electricity, ξ_{out} (kWh $kg_{H_2}^{-1}$) is then given by Eq. S25, where LHV_{H_2} is the lower heating value of H_2 (33.3 kWh $kg_{H_2}^{-1}$), and η_{burner} (-) is thermal efficiency of heat transfer from the H_2 combuster to the packed bed of MgH_2 (*e.g.* by heating the heat-transfer oil in a boiler, or using a fired reactor).

$$\xi_{out} = \xi_{fc} \left(1 - \frac{\Delta H_{H_2}}{\eta_{burner} \text{LHV}_{H_2}}\right) \tag{S25}$$

Therefore, if $\eta_{burner} > \frac{\xi_{fc}}{\text{LHV}_{H_2}}$ (*i.e.* greater energy efficiency than the fuel cell), the round-trip efficiency of the system can be improved by using hydrogen combustion for heating during hydrogen release, as opposed to electrical heating, up to a maximum of $\eta_{burner} \rightarrow 1$, $\xi_{out} \rightarrow 8.5$ kWh $kg_{H_2}^{-1}$, hence giving $\eta_{RT,noheatstorage} \approx 0.16$ under base-case assumptions, somewhat closer in efficiency to systems incorporating TCES. However, safety considerations regarding hydrogen combustion, and difficulties in startup [72], might render the modified system impractical.

5 Supplementary Note 5: Additional parameters for techno-economic analysis

For each of the base, optimistic, and pessimistic modelling scenarios, the capital and operating costs were estimated using reported values and correlations from literature, summarised in Table S5. To account for inflation, historic cost data (older than 2019) reported in the table were converted from the reported value to $\$_{2019}$ (*i.e.* using prices in 2019 as a basis) using the Chemical Engineering Plant Cost Index (CEPCI) [73], noting that different relative price trends between different items of expenditure introduces additional uncertainty into the overall estimated cost.

Supplementary Table S5 Additional parameters used for estimation of capital and operating costs for each scenario, with corresponding literature source(s). C = Turbine capacity (kW or MW), A = heat exchanger area (m^2). Reference years correspond to the publication date of the cited literature source, with historic cost correlations converted into $\$_{2019}$ using CEPCI to account for inflation within the techno-economic model.

Parameter	Unit	Base	Optimistic	Pessimistic	Ref(s)	Ref. year(s)
Electrolyser efficiency	kg_{H_2} kWh^{-1}	0.019	0.023	0.016	[52, 74]	2019, 2020
Electrolyser stack replacement cost	$\%_{install}$	40	20	80	[75, 76]	2019, 2021
Fuel cell efficiency	kWh $kg_{H_2}^{-1}$	12.3	13.3	11.7	[77]	2015
Fuel cell stack replacement cost	$\%_{install}$	40	20	80	[75, 76]	2019, 2021
Hydrogen storage alloy capacity	kg_{H_2} kg_{alloy}^{-1}	0.055	0.059	0.03	[36, 39], This work	2023, 2025
Magnesium carbonate cost	\$ kg^{-1}	0.5	0.46	0.6	[78, 79]	2007, 2021
Nitrate cost	\$ kg^{-1}	0.46	0.31	0.61	[80–82]	2002, 2010
Off-shore cabling cost	\$k	3000	325	33000	[19, 83, 84]	2017, 2020, 2024
On-shore windfarm operation cost	\$ MWh^{-1}	3.5	1.5	7	[19, 85]	2019, 2024
Off-shore windfarm operation cost	\$ MWh^{-1}	18	12	32	[86, 87]	2016, 2019
Levelised cost of electricity from gas turbine	\$ MWh^{-1}	69	50	94	[88]	2023
Gas turbine electrical efficiency	-	0.35	0.6	0.2	[89]	2005
Carbon tax	\$ $t_{CO_2}^{-1}$	150	100	200	[90–92]	2019, 2022, 2023
Desalination capital cost	\$ $kg_{H_2O}^{-1}d^{-1}$	1.9	0	2.2	[93]	2017
Battery lifetime	y	8	12	4	[70]	2023
Electrical heater capital cost	\$ kW^{-1}	300	100	500	[94, 95]	2020, 2023
Turbo-machinery capital cost	\$1000 kW^{-1}	$260 + 2.7(C)^{0.75}$	Base - 10%	Base + 10%	[96]	2020
Turbo-machinery isentropic efficiency	-	0.85	0.9	0.7	[97]	2011
Compressor outlet temperature	°C	200	230	200	[67]	2022
Heat exchanger capital cost	\$ kW^{-1}	$31920 + 59.3(A)^{1.2}$	Base - 15%	Base + 15%	[96]	2020
Reactor vessel capital cost	\$ m_{length}^{-1}	11000	Base - 25%	Base + 25%	[61]	2005
Reactor heat losses	kWh $m^{-1}d^{-1}$	31.8	0	38.6	[49, 50]	2007, 2011
Equipment maintenance	$\%_{CAPEX}$ y^{-1}	2	1	2.5	[96]	2020
Oxygen sale price	\$ kg^{-1}	0	0.05	0	[19, 98]	2015, 2024
Discount rate for NPV calculations	%	5	4	7	[19]	2024

S5.1 Estimation of embedded emissions

The embedded life-cycle emissions of major items of capital expenditure were roughly estimated using values from cradle-to-gate life cycle assessments in literature, using the mid-point indicator of global warming potential (kg_{CO_2eq} *per* unit), with values used given in Table S6. Embedded emissions associated with plant construction or items of process equipment not listed were not considered. Emissions associated with windfarm operation include estimated construction emissions (*e.g.* production of concrete used to support offshore turbines) and emissions associated with maintenance (transportation of components, production of lubricating oils *etc.*).

For most system configurations (shown in Fig. S9), the majority of embedded emissions derived from the production of Mg for the hydrogen storage alloy. At present, Mg metal is produced predominantly from dolomite or magnesite ore *via* various processes, including the electrified Bolzano process [99], and the carbothermal Pidgeon process [100]. For the purposes of this study, the emissions associated with the production of Mg using the Bolzano process estimated by Cherubini *et al.* [99] were used, assuming the relatively low carbon intensity Brazilian electricity grid mix as an electricity input, and with potential for further reduction if all-renewable electricity were used instead. However, a majority of current global magnesium output is produced in China *via* the Pidgeon process, using coal as a fuel source [17, 100] (*c.* 4.2 tonnes of coal equivalent *per* tonne of refined Mg), with approximately four times greater CO_2 emissions *per* unit mass of Mg metal produced. Therefore, the uncertainty in the embedded emissions of the system is large, and hence the values reported here should be treated with some caution.

Supplementary Table S6 Parameters used for estimation of life-cycle emissions for each scenario, with corresponding literature source(s). *=Estimated assuming production of Mg-metal from dolomite ore using the semi-electrified Bolzano process.

Item	Estimated embedded carbon	Ref(s).
Offshore windfarm	25 $kg_{CO_2 eq}$ MWh^{-1}	[19, 101–103]
Onshore windfarm	11 $kg_{CO_2 eq}$ MWh^{-1}	[19, 103–106]
PEM electrolyser	33 $g_{CO_2 eq}$ $kg_{H_2}^{-1}$	[107]
PEM fuel cell	24 $kg_{CO_2 eq}$ kW^{-1}	[108]
Batteries	73 $kg_{CO_2 eq}$ kWh^{-1}	[109]
Magnesium-based H_2 storage alloy*	10 $kg_{CO_2 eq}$ kg^{-1}	[99, 100, 110]
MgO-based TCES material	2 $kg_{CO_2 eq}$ kg^{-1}	[111, 112]

6 Supplementary Note 6: Additional modelled parameters and cases

Supplementary Table S7 Stream conditions for conversion of 1 MWh of electricity into stored hydrogen, and subsequent converstion back to electricity, as shown in the Sankey diagram in the main manuscript (Fig. 3). Estimated flow rates assume that energy conversion is spread over a 24 h modelling period, giving units of mass flow *per* hour *per* megawatt-hour of electricity input (kg $h^{-1} MWh_{in}^{-1}$).

Stream	Temperature (°C)	Pressure (bara)	Total mass flow (kg MWh_{in}^{-1})	Day-averaged mass flowrate (kg h^{-1} MWh_{in}^{-1})
		Energy storage		
Electrolyser water feed	25	1	144	6.0
Electrolyser hydrogen outlet	50	20	16	0.7
Electrolyser oxygen outlet	50	20	128	5.3
Hydrogenation reactor feed	352.5	4.5	16	0.7
CO_2 reactor outlet	347.5	0.2	353	14.7
High-pressure CO_2 to pipeline	25	150	353	14.7
		Energy release		
Flue gas input	110	1	4708	196.2
Compressed flue gas to carbonation reactor	352.5	4	4708	196.2
Unreacted reactor outlet (CO_2 + N_2)	352.5	3.8	4355	181.5
CO_2 depleted flue gas exhaust	190	1	4355	181.5
Hydrogen reactor outlet	347.5	4.5	16	0.7
Fuel cell waste water outlet	80	1	144	6.0

Supplementary Table S8 Input parameters and calculated properties for systems shown in Figs. 4 and S7.

**System input parameters**		
**Parameter**	**Units**	**Value**
Nominal load	[kWh d^{-1}]	20000
Overcapacity factor	[-]	0.25
Days of H_2 storage	[d]	6
Reactor temperature	[°C]	350
CO_2 feed fraction	[wt%]	15
CO_2 capture efficiency	[mol%]	0.5
Mg alloy mass	[t]	167
TCES material mass	[t]	478
Battery capacity	[kWh]	3333

**Calculated parameters**			
**Parameter**	**Units**	**Value (Onshore)**	**Value (Offshore)**
Average capacity factor	[-]	0.37	0.59
Windfarm nameplate capacity	[kWh d^{-1}]	67332.0	42674.1
Electrolyser capacity	[kW]	1517.0	765.8
Fuel cell capacity	[kW]	954.7	819.6
Total H_2 stored and released	[t]	198.5	170.6
Total natural gas used	[t]	1198.8	811.1
Total electricity delivered from windfarm	[GWh]	32.5	34.5
Total electricity delivered from fuel cell	[GWh]	2.4	2.1
Total electricity delivered from batteries	[GWh]	0.7	0.6
Total electricity delivered from gas turbines	[GWh]	5.4	3.7
Total curtailed electricity	[GWh]	0.3	0
Total flue gas processed	[t]	33358.3	28552.2
Total CO_2 emitted	[t]	2776.7	1878.6
Total CO_2 captured	[t]	-2501.9	-2141.4
Net CO_2 emissions	[t]	274.9	-262.8
Net carbon intensity	[kg_{CO_2} MWh^{-1}]	7.5	-7.2
Days of emergency battery use	[-]	686	491
Days of gas turbine use	[-]	612	427
Days of net curtailment	[-]	12	0
Number of electrolyser shutdowns	[-]	270	278
Number of fuel cell shutdowns	[-]	271	279
Average electrolyser shutdown period	[d]	3.5	2.6
Average fuel cell shutdown period	[d]	5.2	5.1
Longest period with no H_2 storage or release	[d]	27	16
Average H_2 residence time	[d]	89.4	59.8

Supplementary Table S9 Input parameters and calculated properties for onshore and offshore systems with $f_{OCP} = 0.3$ and 2 days of hydrogen storage.

System input parameters			
**Parameter**	**Units**	**Value**	
Nominal load	[kWh∼d^{-1}]	20000	
Overcapacity factor	[-]	0.3	
Days of H_2 storage	[d]	2	
Reactor temperature	[°C]	350	
CO_2 feed fraction	[wt%]	15	
CO_2 capture efficiency	[mol%]	0.5	
Mg alloy mass	[t]	56	
TCES material mass	[t]	159	
Battery capacity	[kWh]	3333	
**Calculated parameters**			
**Parameter**	**Units**	**Value (Onshore)**	**Value (Offshore)**
Average capacity factor	[-]	0.37	0.59
Windfarm nameplate capacity	[kWh d^{-1}]	70025	44381
Electrolyser capacity	[kW]	1654.4	872.9
Fuel cell capacity	[kW]	891.5	880.9
Total H_2 stored and released	[t]	207	198
Total natural gas used	[t]	854	508
Total electricity delivered from windfarm	[GWh]	31.9	33.5
Total electricity delivered from fuel cell	[GWh]	2.6	2.4
Total electricity delivered from batteries	[GWh]	0.6	0.4
Total electricity delivered from gas turbines	[GWh]	3.9	2.3
Total curtailed electricity	[GWh]	2.2	1.3
Total flue gas processed	[t]	34173	32951
Total CO_2 emitted	[t]	1978	1178
Total CO_2 captured	[t]	-2566	-2471
Net CO_2 emissions	[t]	-588	-1294
Net carbon intensity	[kg$_{CO_2}$ MWh^{-1}]	-16.1	-35.4
Days of emergency battery use	[-]	547	353
Days of gas turbine use	[-]	478	295
Days of net curtailment	[-]	118	107
Number of electrolyser shutdowns	[-]	284	261
Number of fuel cell shutdowns	[-]	283	261
Average electrolyser shutdown period	[d]	3.3	2.7
Average fuel cell shutdown period	[d]	4.9	5.5
Longest period with no H_2 storage or release	[d]	25	16
Average H_2 residence time	[d]	28.6	29.9

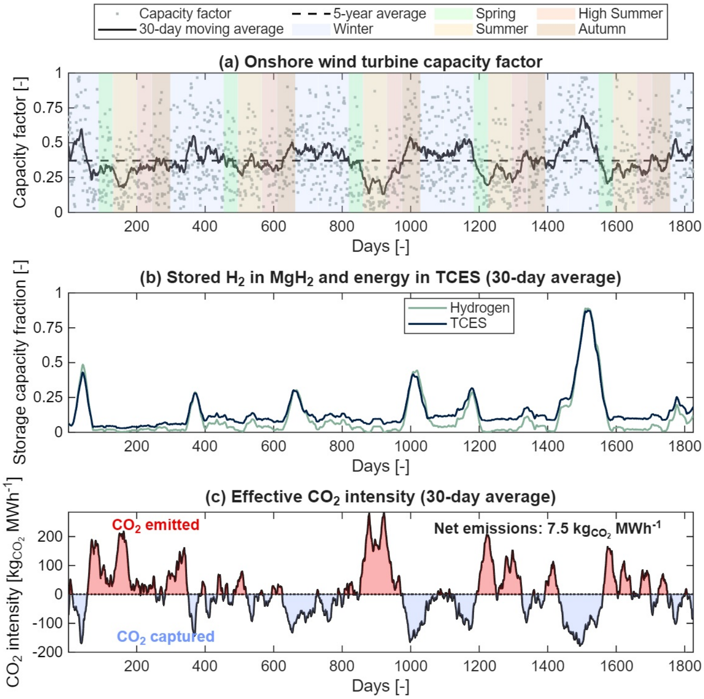

Supplementary Figure S7 Modelling results under base-case assumptions for 5 years of operation of an onshore windfarm with 25% net overcapacity (nameplate capacity: 67300 kWh d^{-1}), and H_2 storage capacity equivalent to 6 days of energy demand (167 tonnes of Mg alloy; 478 tonnes of $MgCO_3$): **(a)** variation in windfarm capacity factor used to estimate power input, **(b)** variation in stored H_2 in MgH_2, and stored heat in TCES material ($MgCO_3$ $\leftrightarrow$ MgO), as a fraction of total capacity, **(c)** estimated carbon intensity of power generation and net carbon intensity over full modelling period.

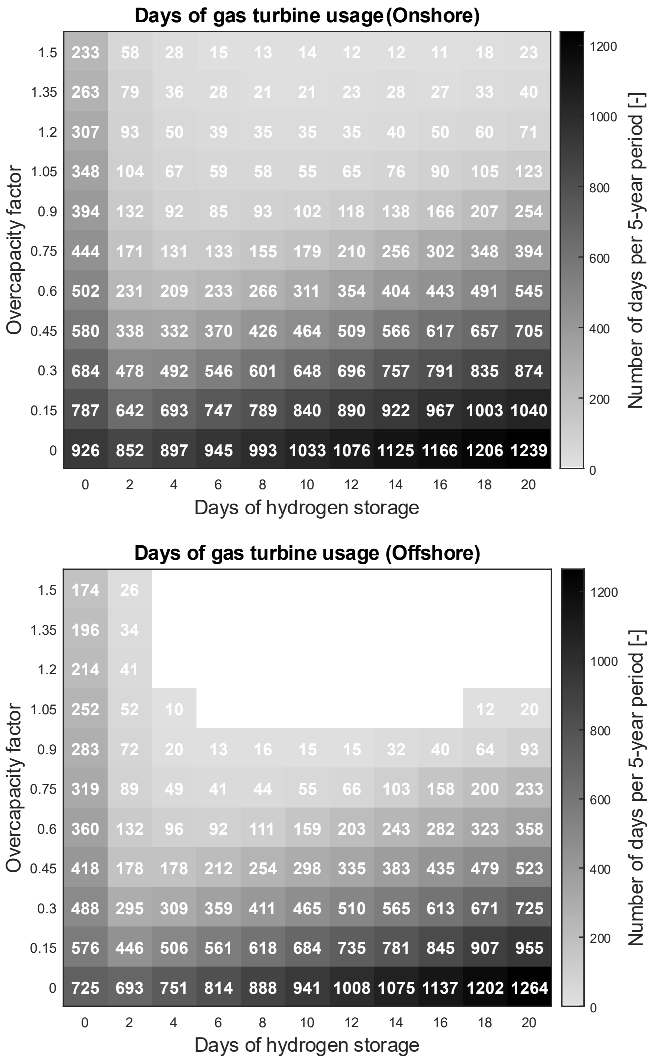

Supplementary Figure S8 Estimated number of days in each 5 year (1825 day) modelling period requiring external energy imports from backup gas turbines for offshore and onshore systems under base-case modelling assumptions. Empty cells correspond to system configurations able to operate with less than five days of external energy imports in the 5-year period.

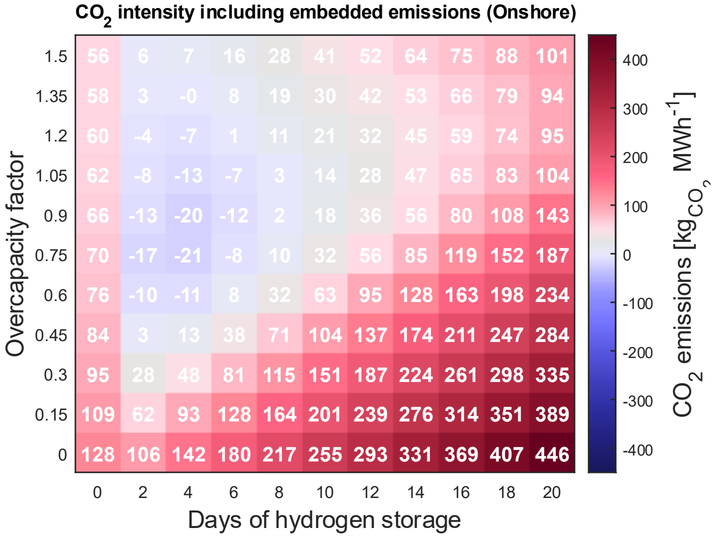

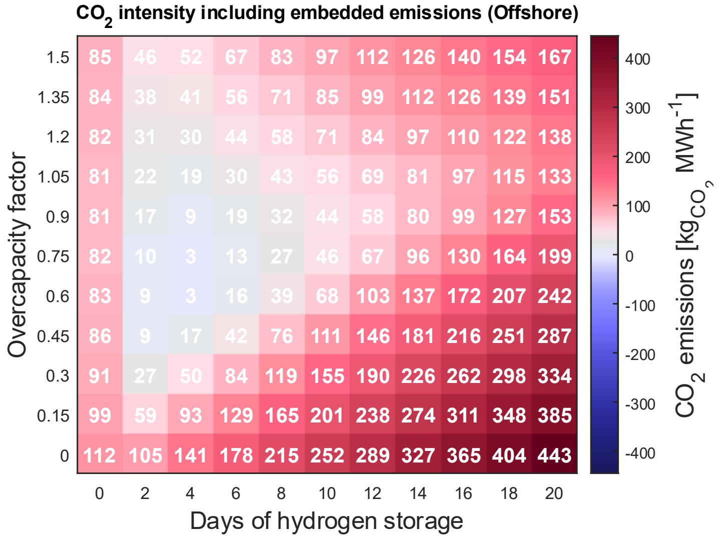

Supplementary Figure S9 Estimated carbon intensity for different offshore and onshore system configurations under base-case operating assumptions, including estimated embedded emissions in construction of the windfarm, PEM electrolyser and fuel cell, and materials used for hydrogen and TCES storage.

7 Supplementary Note 7: Model sensitivity

S7.1 Sensitivity to input parameters: Monte Carlo simulation

To quantify the uncertainty in estimated CAPEX, OPEX, LCOE, and carbon intensity of electricity, Monte Carlo simulations were performed by sampling input values from the ranges reported in Tables 1 and S5. For each parameter, input values were sampled from a triangular distribution, with end points at the maximum and minimum values, and peak at the base value. For each simulation iteration, input values were sampled pseudo-randomly using the in-built `rng` function in MATLAB, using the 'twister' random number generation algorithm with an arbitrary 6-digit seed. The sensitivity of the Monte Carlo simulation to number of iterations and seed used is shown in Fig. S10, indicating convergence at ≥5,000 iterations, and little sensitivity to input seed. For the simulations reported in Figs. 7 and 8, 10,000 iterations were used with seed '771923'.

The relative sensitivity of the model to each input parameter was roughly estimated by calculating the Spearman rank correlation coefficient [113] for each input variable, shown in Fig. S11. For both onshore and offshore system, CAPEX, OPEX, and LCOE showed the greatest sensitivity to assumed windfarm installation and operating costs, hydrogen storage alloy cost and lifetime, and PEM equipment installation cost and lifetime, in line with the CAPEX and OPEX compositions shown in Fig. 7c-f. Carbon intensity showed a strong correlation with assumed gas turbine efficiency and electrolyser efficiency, but no significant correlation with any other parameters.

To further quantify the sensitivity of LCOE to assumed hydrogen storage alloy cost, alloy lifetime, and PEM stack lifetimes, the parameters were varied independently over the range between the optimistic and pessimistic values, shown in Fig. S12. The overall system cost varied linearly with the cost of Mg, with a change of between -5% to +20% in overall LCOE for the optimistic

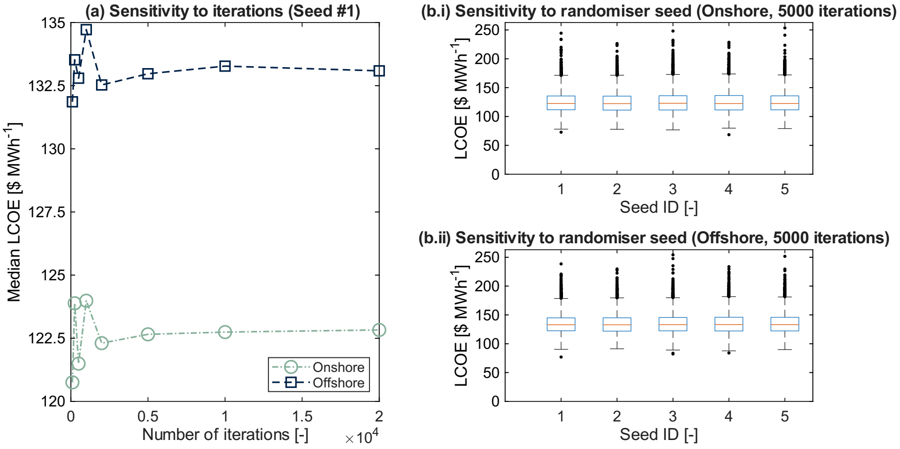

Supplementary Figure S10 (a) Median estimated LCOE from Monte Carlo model as a function of number of model iterations (100-20,000 for onshore and offshore systems, showing convergence after *c.*5,000 iterations. (b) Distribution of estimated LCOE values over 5,000 model iterations for five different randomiser seeds, showing minimal difference in output for different seed inputs.

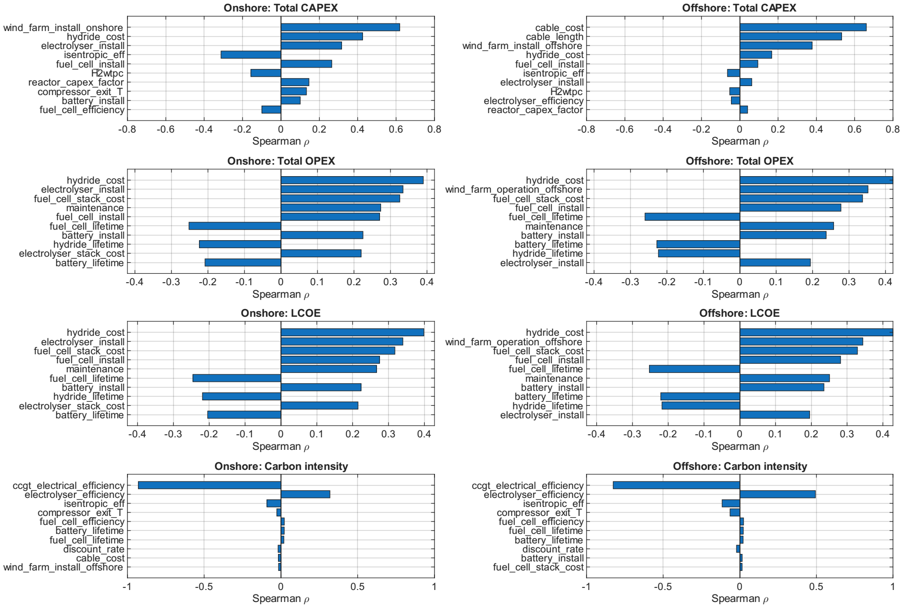

Supplementary Figure S11 Tornado plots showing Spearman rank coefficients (ρ) for different model input parameters varied in the Monte Carlo model on CAPEX, OPEX, LCOE, and carbon intensity of generated electricity. A greater magnitude of ρ indicates a greater relative effect of each input parameter on the simulated output variable, with the sign indicating a positive or negative correlation.

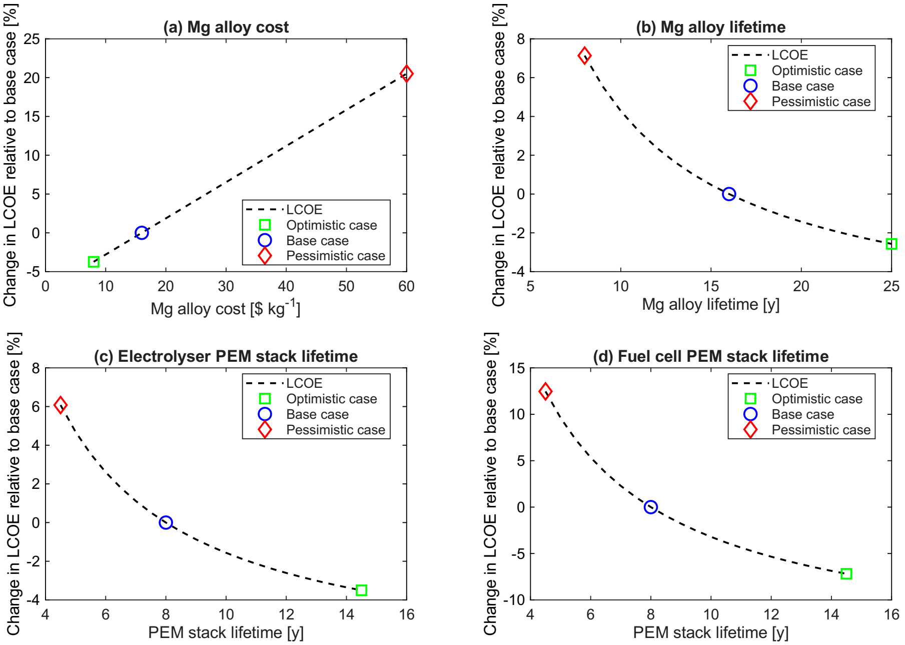

Supplementary Figure S12 Percentage change in levelised cost of electricity (LCOE) relative to base-case assumptions as a function of **(a)** Mg alloy cost *per* unit mass, **(b)** Mg alloy operational lifetime, **(c)** PEM electrolyser stack lifetime, and **(d)** PEM fuel cell stack lifetime, with all other parameters held constant for each case. Costs under base (blue circles), optimistic (green squares) and pessimistic (red diamonds) cases are shown, based on reported parameter ranges in literature as described in Table S5. Costs were estimated for an onshore windfarm with $f_{OCP} = 0.3$ and 2 days of H_2 storage.

and pessimistic cases respectively. Altering the lifetime of the Mg-alloy or PEM stacks resulted in a non-linear change in overall cost, with a decrease in lifetime relative to the base case resulting in a rapid increase in LCOE, and hence causing the distributions of LCOE from Monte Carlo simulations to be asymmetric, with a skew to towards higher overall costs. Therefore, the overall accuracy of the estimates reported here could be improved by decreasing the range of plausible values for Mg alloy cost, and equipment lifetime under realistic process conditions. Moreover, median estimated costs (CAPEX, OPEX, LCOE) from the Monte Carlo simulation exceeded the values estimated from base case parameters (*i.e.* the median of the input parameter

range), as a result of the non-linearity of costs with respect to several input parameters.

S7.2 Sensitivity to demand model

As discussed in the Methods section of the main manuscript, seasonal variation in electricity demand was estimated using data from the National Energy System Operator (NESO) database, averaged for each season over the period 01/01/2016-31/12/2020, with average demand in winter estimated to be around 22% higher than demand in high summer. In order to determine the sensitivity of the system model to seasonal variation, an older dataset of consumer demand over the period 01/01/1997-31/12/1997 [1], provided by the National Grid to electricity suppliers to estimate customer bills, was used as an alternative demand model, with a comparison of the two models shown in Fig. S13. The older dataset showed a greater variation between maximum and minimum estimated demand, with a difference between winter and high summer of *c.* 64%. Applying the alternative demand model with greater seasonal variation for an onshore system with $f_{OCP} = 0.25$ and 6 days of H_2 storage with base-case input parameters, estimated values of LCOE slightly increased relative to the model derived from NESO data, from 124 to 131 \$ MWh^{-1}.

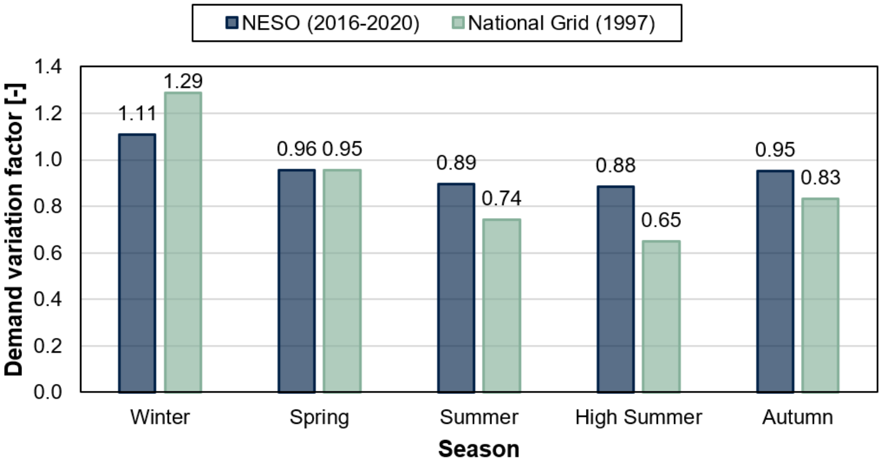

Supplementary Figure S13 Comparison of seasonal variation between demand models based on NESO electricity generation data [2] and National Grid user demand data [1], assuming a 2:1 ratio of domestic and commercial to industrial users. A demand variation factor of 1 corresponds to the daily demand averaged over a full year.

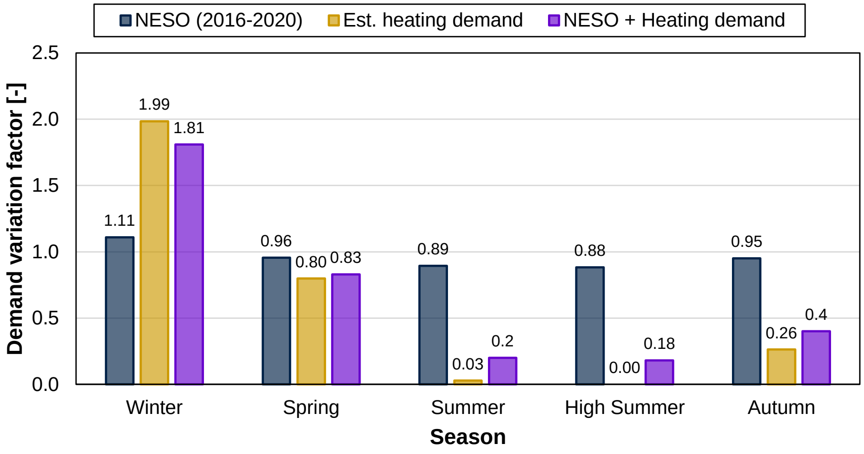

Supplementary Figure S14 Comparison of seasonal variation based on NESO electricity generation data
[2], and estimated energy demand for domestic heating [114] for a dwelling in the South-East of England, assuming that heating demand comprises 80% of total domestic energy usage. A demand variation factor of 1 corresponds to the daily demand averaged over a full year.

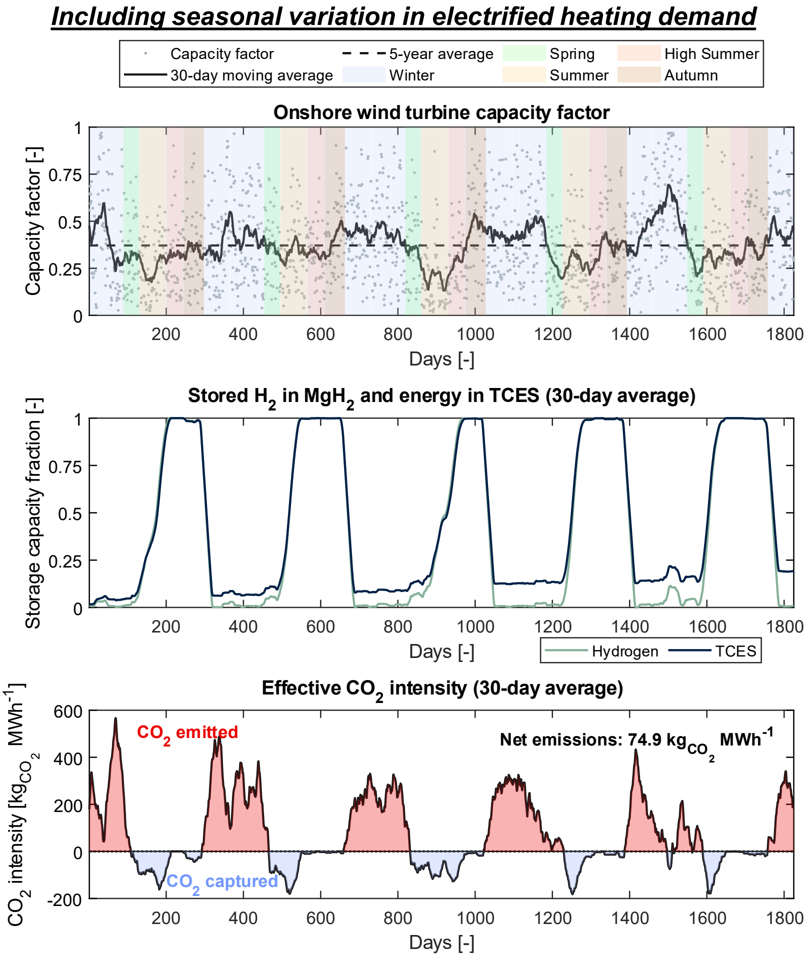

Supplementary Figure S15 Modelling results including energy demand for domestic heating for a dwelling at an arbitrary location in the South-East of England (Lat. 51.3138, Long. -0.6613), with a heating threshold temperature of 14°C. System modelled for 5 years of operation of an onshore windfarm with 25% net overcapacity (nameplate capacity: 67300 kWh d^{-1}), and H_2 storage capacity equivalent to 6 days of energy demand (152 tonnes of Mg alloy; 72 tonnes of $MgCO_3$): **(a)** Variation in windfarm capacity factor used to estimate power input, **(b)** Variation in stored H_2 in MgH_2, and stored heat in TCES material ($MgCO_3 \leftrightarrow MgO$), as a fraction of total capacity, **(c)** Estimated carbon intensity of power generation and net carbon intensity over full modelling period.

S7.3 Sensitivity to initial conditions

For all system configurations reported in the main manuscript, the model was run from 01/01/2016 until 31/12/2020, with the broad seasonal pattern showing a net excess of electricity stored as H_2 in autumn and early winter, and subsequently withdrawn in late winter. Therefore, given real systems would be initiated throughout the year rather than on the 1st of January in all gases, the sensitivity of the model to starting season was investigated. As shown in Table S10, altering the start date had limited effect on LCOE, with an overall range of *c.* 8 \$ MWh^{-1}. Altering the starting season had a greater effect on carbon intensity, with model runs started in summer or high summer showing higher average emissions, as a result of little available wind power during summer and no stored H_2 in the first modelled year. Removing all seasonal variation, while maintaining daily variation, by randomising the order of the 1825 values of daily capacity factor in the data set used, resulted in little change in estimated LCOE, with lower carbon intensity as a result of reduced reliance on gas turbines to make up deficits in energy output during summer when little stored H_2 was available.

Supplementary Table S10 Variation in estimated levelised cost of electricity and carbon intensity as a function of system starting date (*i.e.* time-shifting generation and demand data), with season definitions given in Table S3. 'No seasonal variation' corresponds to a modelling case where daily capacity factor over the 5 year period was randomised to eliminate any seasonal trends, and seasonal variation in demand was removed. The system was modelled under base-case input parameters, with $f_{OCP} = 0.3$ and 2 days of hydrogen storage for all cases.

**Starting date**	**LCOE (\$ MWh^{-1})**		**CO_2 Intensity (kg$_{CO_2}$ MWh^{-1})**	
	Offshore	Onshore	Offshore	Onshore
Midwinter (01/01, Day 1)	112.5	104.4	-39.1	-20.0
Spring (30/03, Day 89)	115.6	106.9	-21.6	-6.3
Summer (11/05, Day 131)	116.7	108.2	-13.0	2.4
High Summer (20/07, Day 201)	112.1	102.8	-13.8	1.2
Autumn (02/09, Day 245)	110.8	101.6	-22.0	-7.5
Winter (26/10, Day 299)	108.9	100.3	-33.5	-15.7
No seasonal variation	109.5	103.5	-50.0	-39.8

S7.4 Sensitivity to windfarm location

In this study, two locations on or near Great Britain were considered for onshore (Kelso) and offshore (North Sea) wind. However, different locations for onshore or offshore wind would be expected to have different wind speed characteristics, in terms of absolute capacity factor and daily variability, affecting the overall system costs and carbon intensity. Therefore, the sensitivity of the system to the windfarm location selected was investigated, in terms of overall levelised cost and carbon intensity of electricity generated, under base, optimisic and pessimistic modelling assumptions. Four additional offshore (reported in Tables S11 and S12) and onshore (reported in Tables S13 and S14) windfarm locations were considered, assuming that electricity demand characteristics would be broadly similar across Northern Europe; further expansion of the model to consider locations with different climates should account for local differences in seasonal demand variation.

Some variation in LCOE was observed between locations was observed, however, for base-case conditions, the estimated values were within 10% of the North Sea or Kelso locations. The North Sea location showed the highest average capacity factor ($\overline{CF}$), and so, as expected [19], was the most favourable offshore location in terms of achieving the lowest LCOE and the highest CO_2 drawdown. Among onshore locations, Viborg (Denmark) and Kuopio (Finland) showed slightly lower overall LCOE than Kelso (Great Britain) (albeit still within ±10%), despite having lower $\overline{CF}$ values, as a result of providing wind power with less daily variability.

Greater differences between locations were observed in estimated CO_2 drawdown as compared to LCOE; however, all windfarms were able to achieve net-negative emissions for the system configuration considered (f_{OCP} = 0.3 and 2 days of hydrogen storage) under base and optimistic modelling assumptions, with some locations showing net positive emissions under pessimistic assumptions. In summary, the locations selected in the main

manuscript were assumed to be broadly representative of wind availability in Northern Europe, allowing general conclusions to be drawn.

Supplementary Table S11 Levelised cost of electricity for alternative offshore windfarm locations. Values in italics represent percentage difference with respect to the North Sea location for the corresponding base, optimistic, or pessimistic modelling case; the system was modelled with $f_{OCP} = 0.3$ and 2 days of hydrogen storage for all cases. Country designations refer to the nearest landmass to which electricity generated offshore could be transmitted, without implying any territorial or jurisdictional claims.

Location	$\overline{CF}$	Latitude	Longitude	Levelised cost of electricity (\$ MWh^{-1})					
				Base		Optimistic		Pessimistic	
North Sea, Great Britain	0.59	56.2911	2.7891	112	*0.0*	68	*0.0*	617	*0.0*
Irish Sea, Great Britain	0.55	53.5392	-4.8616	116	*+3.1%*	78	*+15.0%*	680	*+10.3%*
Skagerrak, Denmark	0.48	58.1083	9.6345	122	*+8.5%*	85	*+25.3%*	713	*+15.6%*
North Atlantic, Ireland	0.58	54.6549	-9.3244	114	*+1.3%*	71	*+4.4%*	620	*+0.5%*
Norwegian Sea, Norway	0.55	71.7493	13.4289	117	*+4.1%*	76	*+11.6%*	600	*-2.8%*

Supplementary Table S12 Carbon intensity of electricity for alternative offshore windfarm locations. Values in italics represent percentage difference with respect to the North Sea location for the corresponding base, optimistic, or pessimistic modelling case; the system was modelled with $f_{OCP} = 0.3$ and 2 days of hydrogen storage for all cases. Country designations refer to the nearest landmass to which electricity generated offshore could be transmitted, without implying any territorial or jurisdictional claims.

Location	$\overline{CF}$	Latitude	Longitude	Carbon Intensity (kg$_{CO_2}$ MWh^{-1})					
				Base		Optimistic		Pessimistic	
North Sea, Great Britain	0.59	56.2911	2.7891	-39.1	*0.0*	-61.7	*0.0*	-9.3	*0.0*
Irish Sea, Great Britain	0.55	53.5392	-4.8616	-28.2	*+28%*	-57.1	*+8%*	25.1	*+369%*
Skagerrak, Denmark	0.48	58.1083	9.6345	-24.4	*+38%*	-54.7	*+11%*	38.9	*+517%*
North Atlantic, Ireland	0.58	54.6549	-9.3244	-36.1	*+8%*	-60.8	*+2%*	12.4	*+233%*
Norwegian Sea, Norway	0.55	71.7493	13.4289	-34.9	*+11%*	-56.7	*+8%*	18.2	*+295%*

Supplementary Table S13 Levelised cost of electricity for alternative onshore windfarm locations. Values in italics represent percentage difference with respect to the Kelso location for the corresponding base, optimistic, or pessimistic modelling case. The system was modelled with f_{OCP} = 0.3 and 2 days of hydrogen storage for all cases.

Location	CF_{avg} (-)	Latitude	Longitude	Levelised cost of electricity (\$ MWh^{-1})					
				Base		Optimistic		Pessimistic	
Kelso, Great Britain	0.37	55.5727	-2.4776	104	*0.0*	75	*0.0*	660	*0.0*
Viborg, Denmark	0.33	56.4528	9.3545	102	*-2.3%*	70	*-6.0%*	678	*+2.7%*
Kuopio, Finland	0.26	62.8925	27.6782	107	*+2.5%*	70	*-6.3%*	642	*-2.7%*
Amiens, France	0.29	49.8606	2.2975	110	*+5.7%*	84	*+11.7%*	732	*+10.8%*
Dresden, Germany	0.22	51.07045	13.8134	111	*+6.3%*	87	*+16.1%*	731	*+10.7%*

Supplementary Table S14 Carbon intensity for alternative onshore windfarm locations. Values in italics represent percentage difference with respect to the Kelso location for the corresponding base, optimistic, or pessimistic modelling case. The system was modelled with f_{OCP} = 0.3 and 2 days of hydrogen storage for all cases.

Location	CF_{avg} (-)	Latitude	Longitude	Carbon Intensity (kg_{CO_2} MWh^{-1})					
				Base		Optimistic		Pessimistic	
Kelso, Great Britain	0.37	55.5727	-2.4776	-20.0	*0*	-53.9	*0*	44.9	*0*
Viborg, Denmark	0.33	56.4528	9.3545	-19.8	*+1%*	-47.9	*+11%*	35.8	*-20%*
Kuopio, Finland	0.26	62.8925	27.6782	-24.1	*-21%*	-55.4	*-3%*	36.2	*-19%*
Amiens, France	0.29	49.8606	2.2975	-6.3	*+69%*	-41.9	*+22%*	68.2	*+52%*
Dresden, Germany	0.22	51.07045	13.8134	-2.6	*+87%*	-34.5	*+36%*	71.6	*+59%*

S7.5 Sensitivity to year order

For each model run, wind speed data over the period 2016-2020 was considered, with annual variation in capacity factor (given in Table S15) assumed to average out over the five year period. However, the performance of the system depended on changes in H_2 inventory over time, and hence the sequence of CF values, in addition to absolute power generation. Therefore, a period starting with a year with much higher or lower wind than the 5-year average could have a disproportionate effect on overall performance. To determine the sensitivity of the model to the year order, simulated 5-year datasets were generated by considering the 120 permutations (5!) of the sequence 2016, 2017, 2018, 2019, 2020, with the resulting frequency distributions of LCOE and CO_2 intensity shown in Fig. S16. Estimated values of LCOE showed an overall range of <0.5 \$ MWh^{-1} for offshore and onshore wind, and carbon intensity showed variation of around 4 kg$_{CO_2}$ MWh^{-1}. Therefore, given the relatively narrow ranges of estimated values relative to other sources of uncertainty, the sensitivity of the model to the sequence of input wind speeds for a given location was assumed to be negligible. However, for datasets encompassing a longer period of wind speeds, years of anomalously low wind well below the long-run average might become significant, necessitating greater total storage capacity [14].

Supplementary Table S15 Average capacity for offshore and onshore wind locations for the 2016-2020 windspeed dataset considered in this work.

**Year**	**Offshore $\overline{CF}$ (North Sea)**	**Onshore $\overline{CF}$ (Kelso)**
2016	0.578	0.344
2017	0.600	0.386
2018	0.577	0.363
2019	0.591	0.363
2020	0.583	0.401
5-year average	0.586	0.371

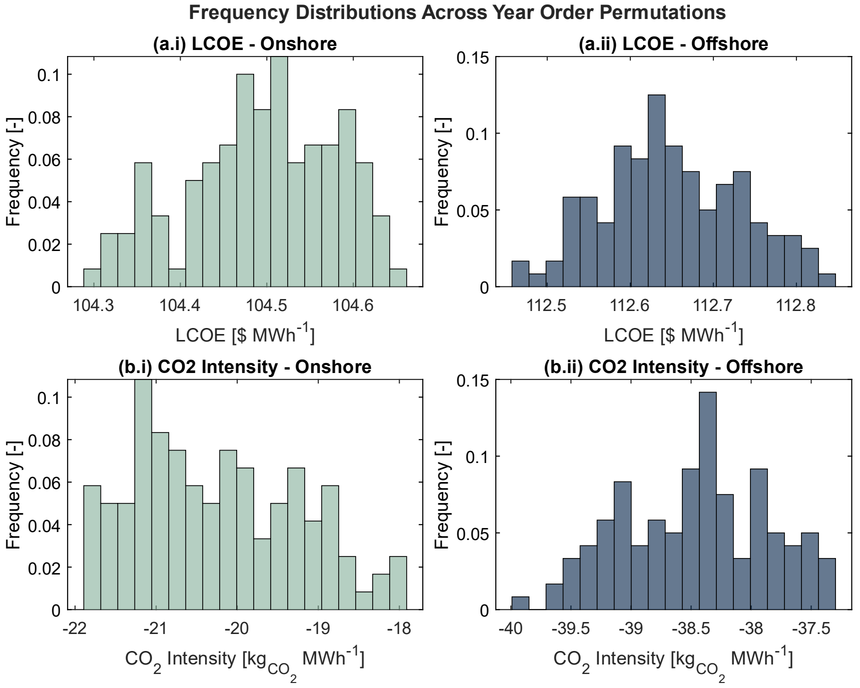

Supplementary Figure S16 Frequency distributions showing estimated **(a)** levelised cost of electricity and **(b)** carbon intensity for onshore and offshore wind for different permutations of input wind data (*i.e.* rearranging the sequence of annual sequence over the period 2016-2020). System was modelled with $f_{OCP} = 0.3$ and 2 days of hydrogen storage for all cases, with base case input parameters.

S7.6 Sensitivity to days with lowest and highest wind speeds

The sensitivity of the model to days with anomalously high or low windspeeds was investigated by setting the n days with highest or lowest CF values in the 1825-day dataset to the 5-year average value (n = 1-50), with results shown in Fig. S17.

For both offshore and and onshore wind, excluding the days with the lowest CF values decreased LCOE, with a sharp decline at $n = 25$ for offshore wind and $n = 6$ for onshore wind. Removing days with the highest CF values had a minimal effect on LCOE, with a very slight decline as a result of decreasing electrolyser capacity (and hence, capital expenditure) required, as all process equipment was scaled in order to minimise curtailment over the five-year period. Therefore, by introducing 'deliberate curtailment' during days with anomalously high wind [115], the capital cost of the system could be reduced by using a smaller electrolyser.

However, all differences were small relative to other sources of uncertainty in the model, and therefore the overall estimates of LCOE and carbon intensity reported in the main manuscript were not influenced excessively by a small number of daily outliers in windspeed.

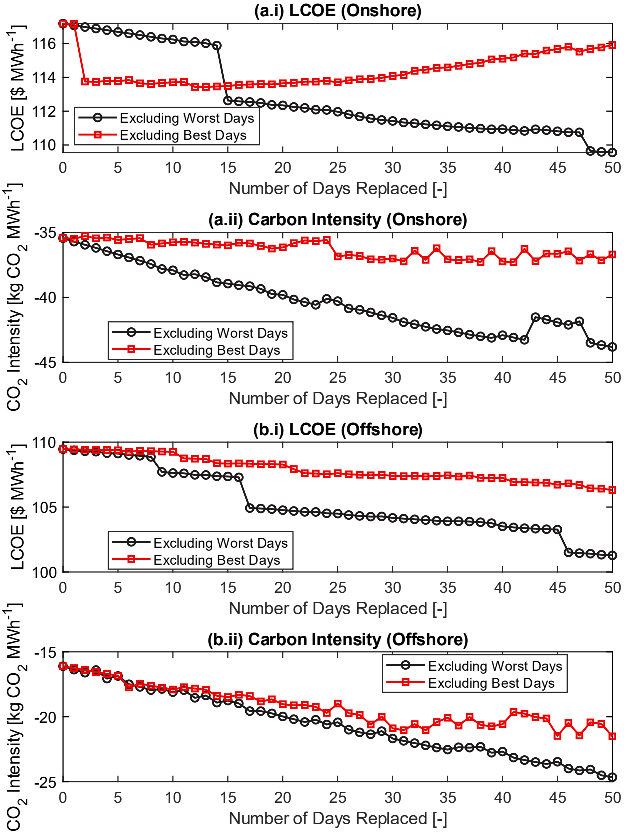

Supplementary Figure S17 Effect of excluding 1-50 days with highest ('best') or lowest ('worst') CF values on levelised cost of electricity and carbon intensity for **(a)** onshore and **(b)** offshore wind. Excluded values were replaced with the 5-year $\overline{CF}$ value. System was modelled with $f_{OCP} = 0.3$ and 2 days of hydrogen storage for all cases, with base case input parameters.

8 Supplementary Note 8: Alternative system configurations

In order to compare the efficiency of the carbonate-based system proposed here to alternative system configurations (hydroxide-based TCES, and storage of hydrogen as a compressed gas or cryogenic liquid), the process model was adapted, with descriptions of altered unit operations given below. Base-case modelling assumptions were used for all alternative modelled systems, as given in Tables S5 and S16.

Supplementary Table S16 Parameters used for estimation of capital and operating costs for alternative H_2 storage systems, with corresponding literature source(s). OPC = ortho- to para- conversion of H_2 spin-isomers, C_{liq} = liquefaction capacity (kg_{H_2} d^{-1}).

Parameter	Unit	Value	Ref(s)
Magnesium hydroxide cost	$ kg^{-1}	0.91	[116]
H_2 compressor exit temperature	°C	150	[67]
H_2 compressor isentropic efficiency	-	0.6	[117]
Compressed H_2 tank cost	$ $kg_{H_2}^{-1}$	550	[118]
Liquid hydrogen tank cost	$ $kg_{H_2}^{-1}$	300	[119]
Liquefaction capital costs	$ $[kg_{H_2}\ d^{-1}]^{-0.8}$	$5.6\times10^6 \cdot (C_{liq}/1000)^{0.8}$	[120, 121]
Liquefaction and OPC energy demand	kWh $kg_{H_2}^{-1}$	7.7	[122]
Liquid H_2 boil-off losses	wt% d^{-1}	1.2	[122–124]

Note that for the relatively low capacity system investigated here, correlations from literature [120, 121] for H_2-liquefaction capital costs were extrapolated below the reported range of values, and so CAPEX estimates for liquefaction have high associated uncertainty.

S8.1 Thermochemical energy storage using magnesium hydroxide

Previous computational and experimental studies [5, 10, 125] on combining hydride-based solid hydrogen storage with thermochemical energy storage have

applied the reversible dehydration of magnesium hydroxide to magnesium oxide ($Mg(OH)_2 \rightarrow MgO + H_2O$) as discussed in the main manuscript. A simplified process flow diagram for hydroxide-based TCES is shown in Fig. S18. The unit operations and stream conditons associated with hydrogen generation, storage, and utilisation (the PEM electrolyser and fuel cell, pumps P1-W and P2-W, heat exchangers HX1-W, HX-2W, HX-4W, and HX5-W, reactor R1, and throttle valves TV1-W and TV2-W) are identical to those shown in the Fig. S3. However, for the TCES system, the compression and intercooling system for compressing flue gas to reactor pressure is removed, and replaced with an electric heater (HX3-W) for producing superheated steam at 19 bara (*i.e.* the equilibrium pH_2O of $MgO \rightleftharpoons Mg(OH)_2$ at 350°C), with water fed from the reverse osmosis purification unit. During periods of energy surplus, excess heat from the hydrogenation reaction in R1 is transferred to R2 using the heat transfer fluid loop, in order to dehydrate $Mg(OH)_2$ to MgO, producing steam at 19 bara, 350°C, which is then passed through a steam turbine in order to recover some energy. Then, during periods of energy deficit, steam raised in the electric heater reacts with MgO in reactor R2, to form $Mg(OH)_2$.

However, given the large energy requirement to raise superheated steam during periods of net electricity deficit (*c.* 0.85 kWh $kg^{-1}_{H_2O}$), the net energy efficiency of the hydroxide based system was considerably lower than the carbonate-based TCES system, with the efficiencies of hydrogen storage and release given in Eqs. S26 and S27, where ΔH_{H_2O} is the enthalpy of the reaction $MgO + H_2O \rightarrow Mg(OH)_2$ (kWh $kg^{-1}_{H_2O}$), $w_{recovery}$ is the work recovered in steam turbine EXP1-W (kWh $kg^{-1}_{H_2O}$), and q_{warmup} is the energy required to heat water from room temperature to superheated steam at reactor conditions, here 350°C, 19 bara (kWh $kg^{-1}_{H_2O}$).

$$\xi_{in} = \frac{\xi_{elec}}{1 - w_{recovery}\xi_{elec}\frac{\Delta H_{H_2}}{\Delta H_{H_2O}}} \tag{S26}$$

$$\xi_{out} = \xi_{fc} - \left(q_{warmup} \frac{\Delta H_{H_2}}{\Delta H_{H_2O}} - q_{cool} \right) \tag{S27}$$

The overall round-trip energy efficiency (η_{RT} = of the H_2 storage system incorporating hydroxide TCES was around 0.10, as compared to 0.22 for carbonate TCES (as shown in Fig. 8). Nevertheless, the hydroxide-based TCES system did show greater round-trip efficiency than MgH_2-based hydrogen storage with no heat storage (with a round-trip efficiency of *c.* 0.04), with potential room for improvement by decreasing the operating temperature of reactors R1 and R2, thereby decreasing the amount of heat required to raise steam.

As in the case of solid-state hydrogen storage without any heat storage, discussed in Section 4, the energy efficiency of systems incorporating hydroxide TCES can be improved by using hydrogen combustion to raise superheated steam instead of electrical heating. Here, the energy efficiency during discharging, using some the released hydrogen to generate heat, is given by Eq. S28. The maximum energy efficiency for a hydrogen-fired boiler with $\eta_{burner} = 1$ tends towards $\xi_{out} = 9.60$ kWh kg_{H_2}, and hence $\eta_{RT,max} = 0.19$.

$$\xi_{out} = \xi_{fc} \left(1 - \frac{q_{warmup}}{\eta_{burner} \mathrm{LHV}_{H_2}} \frac{\Delta H_{H_2}}{\Delta H_{H_2O}} \right) \tag{S28}$$

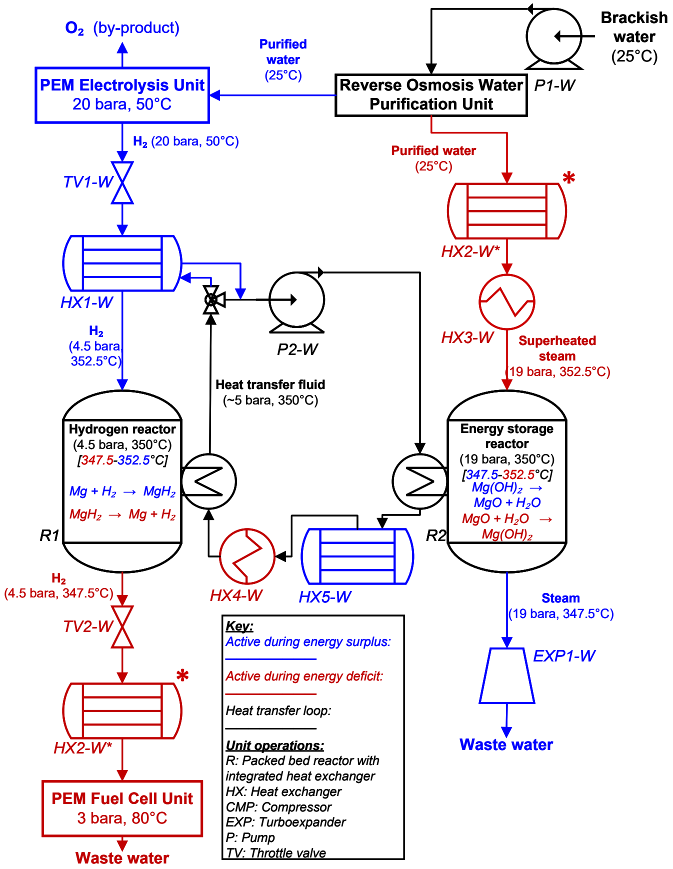

Supplementary Figure S18 Process flow diagram showing main unit operations and approximate stream conditions for coupled hydrogen storage and energy storage using magnesium hydroxide. Streams, reactions, and unit operations are highlighted in blue if operational during periods of net energy surplus, in red if operational during periods of net energy deficit, and in black if operational at all times (* = indicates a single unit for heat integration between streams, shown twice for ease of interpretation).

S8.2 Storage of hydrogen as a compressed gas or cryogenic liquid

In Fig. S19, simplified process flow diagrams for storage of gaseous and liquid hydrogen are shown. In order to estimate the compression work in CMP1a,b-G, the enthalpy departure functions ($H_{dep} = H_{real} - H_{ideal}$) of compressed H_2 were estimated using the Peng-Robinson equation [126, 127].

Assuming an average para-H_2 content in liquid hydrogen of 83%, the liquid storage tank was assumed to lose approximately 1wt% of stored H_2 *per* day to boil-off as a result of exothermic ortho-para conversion [122], and a further 0.2wt% as a result of imperfect tank insulation [123, 124]. The overall energy requirements for liquefaction and ortho-para conversion of hydrogen at 20°C were taken from literature [122]; energy requirements for expansion and heating of the liquid hydrogen to fuel cell conditions were assumed to be negligible.

Gaseous H_2 storage showed the highest round-trip efficiency of the different systems considered ($\eta_{RT,gas} = 0.23$, *i.e.* approximately equal to the pass-through efficiency), exceeding solid MgH_2 storage with carbonate TCES; liquid H_2 showed slightly lower round-trip efficiency, at around $\eta_{RT,liq} = 0.20$.

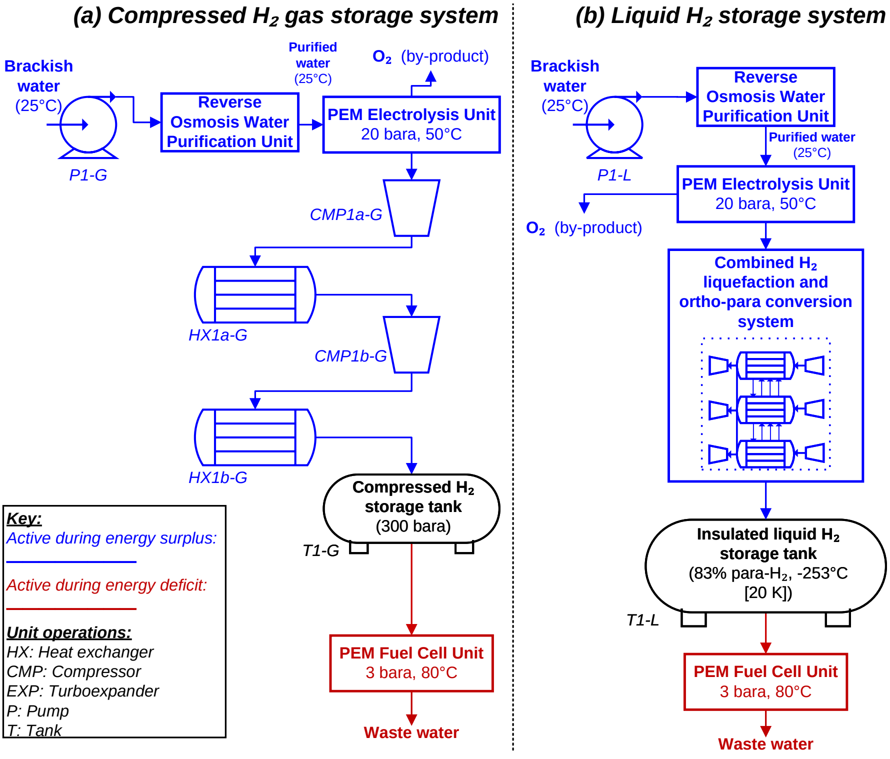

Supplementary Figure S19 Simplified process flow diagrams showing main unit operations and approximate stream conditions for **(a)** storage of hydrogen as a compressed gas and **(b)** storage of hydrogen as a cryogenic liquid. Further process flow diagrams showing the unit operations in the liquefaction process reported elsewhere [122]. Streams, reactions, and unit operations are highlighted in blue if operational during periods of net energy surplus and in red if operational during periods of net energy deficit

Supporting References

[1] UKERC Energy Data Centre. Electricity user load profiles by profile class (2024). URL https://ukerc.rl.ac.uk/DC/cgi-bin/edc_search.pl?GoButton=Detail&WantComp=42&&RELATED=1.

[2] National Energy System Operator. Historic GB Generation Mix (2025). URL https://www.neso.energy/data-portal/historic-generation-mix/historic_gb_generation_mix.

[3] Fruchart, D., Jehan, M., Skryabina, N. & de Rango, P. Hydrogen Solid State Storage on MgH2 Compacts for Mass Applications. *Metals* **13** (5), 992 (2023). https://doi.org/10.3390/met13050992 .

[4] Ren, L., Li, Y., Lin, X., Ding, W. & Zou, J. Promoting hydrogen industry with high-capacity Mg-based solid-state hydrogen storage materials and systems. *Frontiers in Energy* **17** (3), 320–323 (2023). https://doi.org/10.1007/S11708-023-0889-1 .

[5] Lutz, M., Linder, M. & Bürger, I. High capacity, low pressure hydrogen storage based on magnesium hydride and thermochemical heat storage: Experimental proof of concept. *Applied Energy* **271**, 115226 (2020). https://doi.org/10.1016/j.apenergy.2020.115226 .

[6] Maggini, M., Falcucci, G., Rosati, A., Ubertini, S. & Facci, A. L. Non-dimensional numerical analysis of coupled Metal Hydride-Phase Change Material hydrogen storage system. *Journal of Energy Storage* **93**, 112230 (2024). https://doi.org/10.1016/j.est.2024.112230 .

[7] Maggini, M., Facci, A. L., Falcucci, G. & Ubertini, S. Numerical Modeling of Metal Hydride-Phase Change Material Hydrogen Storage Systems with Increased Heat Exchange surface area. *Applied Energy* **378**, 124725 (2025). https://doi.org/10.1016/j.apenergy.2024.124725 .

[8] Mehrpooya, M. & Zhalehrajabi, E. Thermal Management in Hydrogen Storage Tanks Using Metal Hydrides and Phase Change Materials. *Industrial & Engineering Chemistry Research* **63** (50), 21860–21874 (2024). https://doi.org/10.1021/acs.iecr.4c03638 .

[9] Shi, T. & Xu, H. Integration of hydrogen storage and heat storage in thermochemical reactors enhanced with optimized topological structures: Charging process. *Applied Energy* **327**, 120138 (2022). https://doi.org/10.1016/j.apenergy.2022.120138 .

[10] Shi, T., Xu, H., Ke, H. & Zhao, C. Thermal transport of charging/discharging for hydrogen storage in a metal hydride reactor coupled with thermochemical heat storage materials. *Energy Conversion and Management* **273**, 116421 (2022). https://doi.org/10.1016/j.enconman.2022.116421 .

[11] Nyamsi, S., Davids, W. M. & Tolj, I. Experimental investigation and mathematical modeling of a hydrogen storage metal hydride reactor-phase change material system. *International Journal of Hydrogen Energy* **90**, 274–287 (2024). https://doi.org/10.1016/j.ijhydene.2024.10.004 .

[12] Nyamsi, S. N., Davids, W., Tolj, I., Pasupathi, S. & Lototskyy, M. Heat discharge performance of metal hydride thermal battery under different heat transfer conditions: Experimental findings. *Journal of Energy Storage* **72**, 108353 (2023). https://doi.org/10.1016/J.EST.2023.108353 .

[13] Tregambi, C., Mancusi, E., Solimene, R. & Pepe, F. Modeling of an Autothermal Process for Integrated Carbon Dioxide Capture and Methanation by Magnesium Looping (MgO/MgCO3) and Renewable Hydrogen. *Industrial and Engineering Chemistry Research* **62** (51), 22016–22027 (2023). https://doi.org/10.1021/ACS.IECR.3C03139 .

[14] Royal Society. Large-scale electricity storage. Tech. Rep. ISBN: 978-1-78252-666-7, Royal Society, London (2023). URL https://royalsociety.org/electricity-storage.

[15] Xing, H., Scott, S. & Miles, J. Designing cost-efficient, flexible, energy solutions for a decarbonized GB power system. Cambridge Working Papers in Economics CWPE2474, University of Cambridge Energy Policy Research Group, Cambridge (2024). URL https://www.jbs.cam.ac.uk/wp-content/uploads/2024/12/eprg-wp2418.pdf.

[16] Kluger, J. M., Haji, M. N. & Slocum, A. H. The power balancing benefits of wave energy converters in offshore wind-wave farms with energy storage. *Applied Energy* **331**, 120389 (2023). https://doi.org/10.1016/j.apenergy.2022.120389 .

[17] Wang, X. *et al.* A techno-economic study of photovoltaic-solid oxide electrolysis cell coupled magnesium hydride-based hydrogen storage and transportation toward large-scale applications of green hydrogen. *Energy & Environmental Science* **17**, 8429–8456 (2024). https://doi.org/10.1039/D4EE04224G .

[18] Kim, S., Park, J. & Lee, J. H. Design and analysis of wind-based hydrogen production using rule-based operation. *Renewable and Sustainable Energy Reviews* **212**, 115459 (2025). https://doi.org/10.1016/j.rser.2025.115459 .

[19] Fulham, G. J., Mendoza-Moreno, P. V. & Marek, E. J. Managing intermittency of renewable power in sustainable production of methanol, coupled with direct air capture. *Energy & Environmental Science* **17**, 4594–4621 (2024). https://doi.org/10.1039/D4EE00933A .

[20] Zhang, Y. *et al.* Balancing wind-power fluctuation via onsite storage under uncertainty: Power-to-hydrogen-to-power versus lithium battery. *Renewable and Sustainable Energy Reviews* **116**, 109465 (2019). https:

//doi.org/10.1016/j.rser.2019.109465 .

[21] Liang, T., Li, Y., Nie, B., Ahmad, A. & Ding, Y. Achieving a net-zero-carbon energy system in the UK by 2050 with liquid air energy storage. *Energy Conversion and Management* **327**, 119524 (2025). https://doi.org/10.1016/j.enconman.2025.119524 .

[22] Rayit, N. S., Chowdhury, J. I. & Balta-Ozkan, N. Techno-economic optimisation of battery storage for grid-level energy services using curtailed energy from wind. *Journal of Energy Storage* **39**, 102641 (2021). https://doi.org/10.1016/j.est.2021.102641 .

[23] Kumar, S. *et al.* Thermal analysis and optimization of stand-alone microgrids with metal hydride based hydrogen storage. *Sustainable Energy Technologies and Assessments* **52**, 102043 (2022). https://doi.org/10.1016/j.seta.2022.102043 .

[24] Kotowicz, J., Uchman, W., Jurczyk, M. & Sekret, R. Evaluation of the potential for distributed generation of green hydrogen using metal-hydride storage methods. *Applied Energy* **344**, 121269 (2023). https://doi.org/10.1016/j.apenergy.2023.121269 .

[25] Colbertaldo, P., Agustin, S. B., Campanari, S. & Brouwer, J. Impact of hydrogen energy storage on California electric power system: Towards 100% renewable electricity. *International Journal of Hydrogen Energy* **44** (19), 9558–9576 (2019). https://doi.org/10.1016/j.ijhydene.2018.11.062 .

[26] Pedrazzi, S., Zini, G. & Tartarini, P. Modelling and simulation of a wind-hydrogen CHP system with metal hydride storage. *Renewable Energy* **46**, 14–22 (2012). https://doi.org/10.1016/j.renene.2012.03.004 .

[27] Samsatli, S. & Samsatli, N. J. The role of renewable hydrogen and inter-seasonal storage in decarbonising heat – Comprehensive optimisation of future renewable energy value chains. *Applied Energy* **233-234**, 854–893

(2019). https://doi.org/10.1016/j.apenergy.2018.09.159 .

[28] Davis Cortina, M. *et al.* The Integration of Thermal Energy Storage Within Metal Hydride Systems: A Comprehensive Review. *Inorganics* **12** (12), 313 (2024). https://doi.org/10.3390/inorganics12120313 .

[29] Faraj, K., Khaled, M., Faraj, J., Faraj, A. & Castelain, C. Thermal management techniques for enhancing hydrogen adsorption and desorption in metal hydride reactors: a short recent review. *Journal of Physics: Conference Series* **2754** (1), 012030 (2024). https://doi.org/10.1088/1742-6596/2754/1/012030 .

[30] Faraj, K., Faraj, J., Castelain, C. & Khaled, M. Use of PCMs in metal hydride reactors for enhancing hydrogen absorption and desorption: A short recent review. *International Journal of Hydrogen Energy* **109**, 1209–1229 (2025). https://doi.org/10.1016/j.ijhydene.2025.02.183 .

[31] Kukkapalli, V. K., Kim, S. & Thomas, S. A. Thermal Management Techniques in Metal Hydrides for Hydrogen Storage Applications: A Review. *Energies* **16** (8), 3444 (2023). https://doi.org/10.3390/en16083444 .

[32] Cetinkaya, S. A., Disli, T., Soyturk, G., Kizilkan, O. & Colpan, C. O. A Review on Thermal Coupling of Metal Hydride Storage Tanks with Fuel Cells and Electrolyzers. *Energies* **16** (1), 341 (2023). https://doi.org/10.3390/en16010341 .

[33] Donat, F. & Müller, C. R. Prospects of MgO-based sorbents for CO2 capture applications at high temperatures. *Current Opinion in Green and Sustainable Chemistry* **36**, 100645 (2022). https://doi.org/10.1016/j.cogsc.2022.100645 .

[34] Dunstan, M. T., Donat, F., Bork, A. H., Grey, C. P. & Müller, C. R. CO2 Capture at Medium to High Temperature Using Solid Oxide-Based Sorbents: Fundamental Aspects, Mechanistic Insights, and Recent Advances. *Chemical Reviews* **121** (20), 12681–12745 (2021). https://doi.org/10.1021/ACS.CHEMREV.1C00100/ .

[35] Desage, L. *et al.* Thermochemical batteries using metal carbonates: A review of heat storage and extraction. *Journal of Energy Storage* **71** (May), 107901 (2023). https://doi.org/10.1016/j.est.2023.107901 .

[36] Hong, H., Harrison, A. R. P. & Nie, B. Linking the Microstructure of Ball-Milled Mg–Ni Hydrogen Storage Materials to Reactive Properties and Techno-Economic Feasibility. *Energy & Fuels* **39** (28), 1378913800 (2025). https://doi.org/10.1021/acs.energyfuels.5c01986 .

[37] Kondratowicz, T. *et al.* Templated synthesis of multi-hierarchical layered double hydroxide microspheres. *Journal of Materials Chemistry A* **13** (35), 29138–29146 (2025). https://doi.org/10.1039/D5TA01511A .

[38] Dal Pozzo, A., Armutlulu, A., Rekhtina, M., Abdala, P. M. & Müller, C. R. CO2 Uptake and Cyclic Stability of MgO-Based CO2 Sorbents Promoted with Alkali Metal Nitrates and Their Eutectic Mixtures. *ACS Applied Energy Materials* **2** (2), 1295–1307 (2019). https://doi.org/10.1021/ACSAEM.8B01852 .

[39] Lin, X. *et al.* A one- and three-dimensional coupled model and simulation investigation for the large-scale oil-heating type Mg-based hydrogen storage tank. *Chemical Engineering Journal* **472**, 144943 (2023). https://doi.org/10.1016/j.cej.2023.144943 .

[40] Huang, K. *et al.* Disclosing the effects of heating strategies on the desorption performance of Mg-based hydrogen storage tanks at different scales. *International Journal of Hydrogen Energy* **106**, 1134–1143 (2025).

https://doi.org/10.1016/j.ijhydene.2025.02.055 .

[41] McCoy, S. T. & Rubin, E. S. An engineering-economic model of pipeline transport of CO2 with application to carbon capture and storage. *International Journal of Greenhouse Gas Control* **2** (2), 219–229 (2008). https://doi.org/10.1016/S1750-5836(07)00119-3 .

[42] Kim, T. W., Yoon, H. C. & Lee, J. Y. Review on carbon capture and storage (CCS) from source to sink; part 1: Essential aspects for CO2 pipeline transportation. *International Journal of Greenhouse Gas Control* **137**, 104208 (2024). https://doi.org/10.1016/j.ijggc.2024.104208 .

[43] Artanto, Y. *et al.* Performance of MEA and amine-blends in the CSIRO PCC pilot plant at Loy Yang Power in Australia. *Fuel* **101**, 264–275 (2012). https://doi.org/10.1016/J.FUEL.2012.02.023 .

[44] Papalas, T., Antzaras, A. & Lemonidou, A. Unveiling the dynamic CO2 capture performance of MgO promoted with molten salts and CaCO3 via fixed bed reactor experiments. *Reaction Chemistry & Engineering* **10**, 168–176 (2025). https://doi.org/10.1039/D4RE00432A .

[45] Saghafifar, M., Schnellmann, M. A. & Scott, S. A. Chemical looping electricity storage. *Applied Energy* **279**, 115553 (2020). https://doi.org/10.1016/J.APENERGY.2020.115553 .

[46] Saghafifar, M. & Scott, S. A. The use of high decomposition temperature materials for chemical looping electricity storage. *Chemical Engineering Journal* **423**, 128789 (2021). https://doi.org/10.1016/J.CEJ.2021.128789 .

[47] Saghafifar, M., Schnellmann, M. A. & Scott, S. A. Limits of performance of chemical looping air separation in packed bed coupled with electricity production. *International Journal of Greenhouse Gas Control* **118**, 103668 (2022). https://doi.org/10.1016/J.IJGGC.2022.103668 .

[48] Abdi Lanbaran, D., Wang, C., Wen, C., Wu, Z. & Li, B. Modeling the impact of temperature-dependent thermal conductivity on hydrogen desorption from magnesium hydride. *International Journal of Hydrogen Energy* **138**, 491–508 (2025). https://doi.org/10.1016/j.ijhydene.2025.05.128 .

[49] Incropera, F., Dewitt, D., Bergman, T. & Lavine, A. *Fundamentals of heat and mass transfer* (John Wiley & Sons, Ltd, Hoboken, NJ, 2007).

[50] Hänchen, M., Brückner, S. & Steinfeld, A. High-temperature thermal storage using a packed bed of rocks – Heat transfer analysis and experimental validation. *Applied Thermal Engineering* **31** (10), 1798–1806 (2011). https://doi.org/10.1016/j.applthermaleng.2010.10.034 .

[51] Papalas, T., Antzaras, A. N. & Lemonidou, A. A. Magnesite-derived MgO promoted with molten salts and limestone as highly-efficient CO2 sorbent. *Journal of CO2 Utilization* **53**, 101725 (2021). https://doi.org/10.1016/J.JCOU.2021.101725 .

[52] Dieterich, V., Buttler, A., Hanel, A., Spliethoff, H. & Fendt, S. Power-to-liquid *via* synthesis of methanol, DME or Fischer–Tropsch-fuels: a review. *Energy & Environmental Science* **13** (10), 3207–3252 (2020). https://doi.org/10.1039/D0EE01187H .

[53] Wu, J. *et al.* A review of PEM fuel cell durability: Degradation mechanisms and mitigation strategies. *Journal of Power Sources* **184** (1), 104–119 (2008). https://doi.org/10.1016/j.jpowsour.2008.06.006 .

[54] Kuhnert, E. *et al.* Impact of intermittent operation on photovoltaic-PEM electrolyzer systems: A degradation study based on accelerated stress testing. *International Journal of Hydrogen Energy* **55**, 683–695 (2024). https://doi.org/10.1016/j.ijhydene.2023.11.249 .

[55] Nguyen, E., Olivier, P., Pera, M.-C., Pahon, E. & Roche, R. Impacts of intermittency on low-temperature electrolysis technologies: A comprehensive review. *International Journal of Hydrogen Energy* **70**, 474–492 (2024). https://doi.org/10.1016/j.ijhydene.2024.05.217 .

[56] Ward, K. R., Bamisile, O., Ejiyi, C. J. & Staffell, I. Time-averaged wind power data hides variability critical to renewables integration. *Energy Strategy Reviews* **50**, 101235 (2023). https://doi.org/10.1016/j.esr.2023.101235 .

[57] van der Roest, E., Bol, R., Fens, T. & van Wijk, A. Utilisation of waste heat from PEM electrolysers – Unlocking local optimisation. *International Journal of Hydrogen Energy* **48** (72), 27872–27891 (2023). https://doi.org/10.1016/j.ijhydene.2023.03.374 .

[58] Scheepers, F. *et al.* Temperature optimization for improving polymer electrolyte membrane-water electrolysis system efficiency. *Applied Energy* **283**, 116270 (2021). https://doi.org/10.1016/j.apenergy.2020.116270 .

[59] Wirkert, F. J. *et al.* A modular design approach for PEM electrolyser systems with homogeneous operation conditions and highly efficient heat management. *International Journal of Hydrogen Energy* **45** (2), 1226–1235 (2020). https://doi.org/10.1016/j.ijhydene.2019.03.185 .

[60] Liu, J., Zhao, M. & Rong, L. Overview of hydrogen-resistant alloys for high-pressure hydrogen environment: on the hydrogen energy structural materials. *Clean Energy* **7** (1), 99–115 (2023). https://doi.org/10.1093/ce/zkad009 .

[61] Sinnott, R. *Chemical Engineering Design* 4th edn. No. 6 in Coulson & Richardson's Chemical Engineering (Elsevier Butterworth-Heinemann, Oxford, 2005).

[62] de Rango, P., Marty, P. & Fruchart, D. Hydrogen storage systems based on magnesium hydride: from laboratory tests to fuel cell integration. *Applied Physics A* **122** (2), 126 (2016). https://doi.org/10.1007/s00339-016-9646-1 .

[63] Amrollahi, Z., Ystad, P. A. M., Ertesvåg, I. S. & Bolland, O. Optimized process configurations of post-combustion CO2 capture for natural-gas-fired power plant – Power plant efficiency analysis. *International Journal of Greenhouse Gas Control* **8**, 1–11 (2012). https://doi.org/10.1016/j.ijggc.2012.01.005 .

[64] Dow Chemical Company. DOWTHERM™ A Heat Transfer Fluid | Dow Inc. (2025). URL https://www.dow.com/en-us/pdp.dowtherm-a-heat-transfer-fluid.238000z.html#overview.

[65] NIST. Thermophysical Properties of Fluid Systems (2022). URL https://webbook.nist.gov/chemistry/fluid/.

[66] Ibrahim, T. K. & Rahman, M. M. Effects of Isentropic Efficiency and Enhancing Strategies on Gas Turbine Performance. *Journal of Mechanical Engineering and Sciences* **4**, 383–396 (2013). https://doi.org/10.15282/jmes.4.2013.3.0036 .

[67] Tahan, M.-R. Recent advances in hydrogen compressors for use in large-scale renewable energy integration. *International Journal of Hydrogen Energy* **47** (83), 35275–35292 (2022). https://doi.org/10.1016/j.ijhydene.2022.08.128 .

[68] Jackson, S. & Brodal, E. Optimization of the Energy Consumption of a Carbon Capture and Sequestration Related Carbon Dioxide Compression Processes. *Energies* **12** (9), 1603 (2019). https://doi.org/10.3390/en12091603 .

[69] Erans, M. *et al.* Direct air capture: process technology, techno-economic and socio-political challenges. *Energy & Environmental Science* **15** (4), 1360–1405 (2022). https://doi.org/10.1039/D1EE03523A .

[70] Ohrelius, M., Berg, M., Wreland Lindström, R. & Lindbergh, G. Lifetime Limitations in Multi-Service Battery Energy Storage Systems. *Energies* **16** (7), 3003 (2023). https://doi.org/10.3390/en16073003 .

[71] Denholm, P., Cole, W. & Blair, N. Moving Beyond 4-Hour Li-Ion Batteries: Challenges and Opportunities for Long(er)-Duration Energy Storage. Tech. Rep. NREL/TP-6A40-85878, National Renewable Energy Laboratory (NREL), Golden, CO (United States) (2023). URL https://www.osti.gov/biblio/2000002.

[72] Biswas, S., Moreno Sader, K. & Green, W. H. Perspective on Decarbonizing Long-Haul Trucks Using Onboard Dehydrogenation of Liquid Organic Hydrogen Carriers. *Energy & Fuels* **37** (22), 17003–17012 (2023). https://doi.org/10.1021/acs.energyfuels.3c01919 .

[73] The Chemical Engineering Plant Cost Index ® (2014). URL https://www.chemengonline.com/pci-home/.

[74] Shiva Kumar, S. & Himabindu, V. Hydrogen production by PEM water electrolysis – A review. *Materials Science for Energy Technologies* **2** (3), 442–454 (2019). https://doi.org/10.1016/j.mset.2019.03.002 .

[75] Khan, M. H. A. *et al.* Designing optimal integrated electricity supply configurations for renewable hydrogen generation in Australia. *iScience* **24** (6) (2021). https://doi.org/10.1016/j.isci.2021.102539 .

[76] Mayyas, A. & Mann, M. Manufacturing competitiveness analysis for hydrogen refueling stations. *International Journal of Hydrogen Energy* **44** (18), 9121–9142 (2019). https://doi.org/10.1016/j.ijhydene.2019.02.135 .

[77] Staffell, I. Zero carbon infinite COP heat from fuel cell CHP. *Applied Energy* **147**, 373–385 (2015). https://doi.org/10.1016/j.apenergy.2015.02.089 .

[78] Mahon, D., Claudio, G. & Eames, P. An Experimental Study of the Decomposition and Carbonation of Magnesium Carbonate for Medium Temperature Thermochemical Energy Storage. *Energies* **14** (5), 1316 (2021). https://doi.org/10.3390/EN14051316 .

[79] Teir, S., Kuusik, R., Fogelholm, C.-J. & Zevenhoven, R. Production of magnesium carbonates from serpentinite for long-term storage of CO2. *International Journal of Mineral Processing* **85** (1), 1–15 (2007). https://doi.org/10.1016/j.minpro.2007.08.007 .

[80] Kenisarin, M. M. High-temperature phase change materials for thermal energy storage. *Renewable and Sustainable Energy Reviews* **14** (3), 955–970 (2010). https://doi.org/10.1016/J.RSER.2009.11.011 .

[81] Herrmann, U. & Kearney, D. W. Survey of Thermal Energy Storage for Parabolic Trough Power Plants. *Journal of Solar Energy Engineering* **124** (2), 145–152 (2002). https://doi.org/10.1115/1.1467601 .

[82] Pacheco, J. E., Showalter, S. K. & Kolb, W. J. Development of a Molten-Salt Thermocline Thermal Storage System for Parabolic Trough Plants. *Journal of Solar Energy Engineering* **124** (2), 153–159 (2002). https://doi.org/10.1115/1.1464123 .

[83] Díaz, H. & Guedes Soares, C. Review of the current status, technology and future trends of offshore wind farms. *Ocean Engineering* **209**, 107381 (2020). https://doi.org/10.1016/j.oceaneng.2020.107381 .

[84] Gonzalez-Rodriguez, A. G. Review of offshore wind farm cost components. *Energy for Sustainable Development* **37**, 10–19 (2017). https://doi.org/10.1016/j.esd.2016.12.001 .

[85] Wiser, R., Bolinger, M. & Lantz, E. Assessing wind power operating costs in the United States: Results from a survey of wind industry experts. *Renewable Energy Focus* **30**, 46–57 (2019). https://doi.org/10.1016/j.ref.2019.05.003 .

[86] Judge, F. *et al.* A lifecycle financial analysis model for offshore wind farms. *Renewable and Sustainable Energy Reviews* **103**, 370–383 (2019). https://doi.org/10.1016/j.rser.2018.12.045 .

[87] Shafiee, M., Brennan, F. & Espinosa, I. A. A parametric whole life cost model for offshore wind farms. *The International Journal of Life Cycle Assessment* **21** (7), 961–975 (2016). https://doi.org/10.1007/s11367-016-1075-z .

[88] Department for Energy Security & Net Zero. Electricity generation costs 2023 (2023). URL https://assets.publishing.service.gov.uk/media/6556027d046ed400148b99fe/electricity-generation-costs-2023.pdf.

[89] Poullikkas, A. An overview of current and future sustainable gas turbine technologies. *Renewable and Sustainable Energy Reviews* **9** (5), 409–443 (2005). https://doi.org/10.1016/j.rser.2004.05.009 .

[90] Rennert, K. *et al.* Comprehensive evidence implies a higher social cost of CO2. *Nature* **610** (7933), 687–692 (2022). https://doi.org/10.1038/s41586-022-05224-9 .

[91] Pindyck, R. S. The social cost of carbon revisited. *Journal of Environmental Economics and Management* **94**, 140–160 (2019). https://doi.org/10.1016/j.jeem.2019.02.003 .

[92] Köppl, A. & Schratzenstaller, M. Carbon taxation: A review of the empirical literature. *Journal of Economic Surveys* **37** (4), 1353–1388 (2023). https://doi.org/10.1111/joes.12531 .

[93] Caldera, U. & Breyer, C. Learning Curve for Seawater Reverse Osmosis Desalination Plants: Capital Cost Trend of the Past, Present, and Future. *Water Resources Research* **53** (12), 10523–10538 (2017). https://doi.org/10.1002/2017WR021402 .

[94] Pardillos-Pobo, D., González-Gómez, P. A., Laporte-Azcué, M. & Santana, D. Thermo-economic design of an electric heater to store renewable curtailment in solar power tower plants. *Energy Conversion and Management* **297**, 117710 (2023). https://doi.org/10.1016/j.enconman.2023.117710 .

[95] Okazaki, T. Electric thermal energy storage and advantage of rotating heater having synchronous inertia. *Renewable Energy* **151**, 563–574 (2020). https://doi.org/10.1016/j.renene.2019.11.051 .

[96] Sinnott, R. & Towler, G. *Chemical Engineering Design* 2nd edn (Elsevier, Oxford, 2020). URL https://linkinghub.elsevier.com/retrieve/pii/C20170015550.

[97] Rahman, M. M., Ibrahim, T. K. & Abdalla, A. N. Thermodynamic performance analysis of gas-turbine power-plant. *International Journal of Physical Sciences* **6** (14), 3539–3550 (2011). https://doi.org/10.5897/IJPS11.272 .

[98] Ebrahimi, A., Meratizaman, M., Akbarpour Reyhani, H., Pourali, O. & Amidpour, M. Energetic, exergetic and economic assessment of oxygen production from two columns cryogenic air separation unit. *Energy* **90**, 1298–1316 (2015). https://doi.org/10.1016/j.energy.2015.06.083 .

[99] Cherubini, F., Raugei, M. & Ulgiati, S. LCA of magnesium production: Technological overview and worldwide estimation of environmental burdens. *Resources, Conservation and Recycling* **52** (8), 1093–1100 (2008). https://doi.org/10.1016/j.resconrec.2008.05.001 .

[100] Gao, F., Nie, Z., Wang, Z., Gong, X. & Zuo, T. Life cycle assessment of primary magnesium production using the Pidgeon process in China. *The International Journal of Life Cycle Assessment* **14** (5), 480–489 (2009). https://doi.org/10.1007/s11367-009-0101-9 .

[101] Li, Q. *et al.* Life cycle assessment and life cycle cost analysis of a 40 MW wind farm with consideration of the infrastructure. *Renewable and Sustainable Energy Reviews* **138**, 110499 (2021). https://doi.org/10.1016/j.rser.2020.110499 .

[102] Verma, S., Paul, A. R. & Haque, N. Selected Environmental Impact Indicators Assessment of Wind Energy in India Using a Life Cycle Assessment. *Energies* **15** (11), 3944 (2022). https://doi.org/10.3390/en15113944 .

[103] Bonou, A., Laurent, A. & Olsen, S. I. Life cycle assessment of onshore and offshore wind energy-from theory to application. *Applied Energy* **180**, 327–337 (2016). https://doi.org/10.1016/j.apenergy.2016.07.058 .

[104] Garcia-Teruel, A., Rinaldi, G., Thies, P. R., Johanning, L. & Jeffrey, H. Life cycle assessment of floating offshore wind farms: An evaluation of operation and maintenance. *Applied Energy* **307**, 118067 (2022). https://doi.org/10.1016/j.apenergy.2021.118067 .

[105] Kaldellis, J. K. & Apostolou, D. Life cycle energy and carbon footprint of offshore wind energy. Comparison with onshore counterpart. *Renewable Energy* **108**, 72–84 (2017). https://doi.org/10.1016/j.renene.2017.02.039 .

[106] Yang, J. *et al.* The life-cycle energy and environmental emissions of a typical offshore wind farm in China. *Journal of Cleaner Production* **180**, 316–324 (2018). https://doi.org/10.1016/j.jclepro.2018.01.082 .

[107] Iyer, R. K., Prosser, J. H., Kelly, J. C., James, B. D. & Elgowainy, A. Life-cycle analysis of hydrogen production from water electrolyzers.

International Journal of Hydrogen Energy **81**, 1467–1478 (2024). https://doi.org/10.1016/j.ijhydene.2024.06.355 .

[108] Mori, M. *et al.* Life cycle sustainability assessment of a proton exchange membrane fuel cell technology for ecodesign purposes. *International Journal of Hydrogen Energy* **48** (99), 39673–39689 (2023). https://doi.org/10.1016/j.ijhydene.2023.05.255 .

[109] Frischknecht, R. *et al.* The ecoinvent Database: Overview and Methodological Framework (7 pp). *The International Journal of Life Cycle Assessment* **10** (1), 3–9 (2005). https://doi.org/10.1065/lca2004.10.181.1 .

[110] Mistry, M., Gediga, J. & Boonzaier, S. Life cycle assessment of nickel products. *The International Journal of Life Cycle Assessment* **21** (11), 1559–1572 (2016). https://doi.org/10.1007/s11367-016-1085-x .

[111] Shahbaz, F., Singh, I., Krishnan, P. & Celik, K. Life cycle assessment of brucite and synthetic MgO produced from reject brine using different alkalis. *Journal of Cleaner Production* **380**, 135071 (2022). https://doi.org/10.1016/j.jclepro.2022.135071 .

[112] Gaidajis, G. & Kakanis, I. Life Cycle Assessment of Nitrate and Compound Fertilizers Production—A Case Study. *Sustainability* **13** (1), 148 (2021). https://doi.org/10.3390/su13010148 .

[113] Zar, J. H. in *Spearman Rank Correlation* (John Wiley & Sons, Ltd, 2005). URL https://onlinelibrary.wiley.com/doi/abs/10.1002/0470011815.b2a15150. _eprint: https://onlinelibrary.wiley.com/doi/pdf/10.1002/0470011815.b2a15150.

[114] Staffell, I., Pfenninger, S. & Johnson, N. A global model of hourly space heating and cooling demand at multiple spatial scales. *Nature Energy* **8** (12), 1328–1344 (2023). https://doi.org/10.1038/s41560-023-01341-5 .

[115] Smith, C. & Torrente-Murciano, L. Cost efficiency versus energy utilization in green ammonia production from intermittent renewable energy. *Nature Chemical Engineering* **2** (4), 261–272 (2025). https://doi.org/10.1038/s44286-025-00207-9 .

[116] ECHEMI. Magnesium hydroxide Price and Market Analysis (2022). URL https://www.echemi.com/productsInformation/pid_Seven9059-magnesiumhydroxide.html.

[117] Franco, A. & Giovannini, C. Hydrogen Gas Compression for Efficient Storage: Balancing Energy and Increasing Density. *Hydrogen* **5** (2), 293–311 (2024). https://doi.org/10.3390/hydrogen5020017 .

[118] Shin, H. K. & Ha, S. K. A Review on the Cost Analysis of Hydrogen Gas Storage Tanks for Fuel Cell Vehicles. *Energies* **16** (13), 5233 (2023). https://doi.org/10.3390/en16135233 .

[119] Marcinkoski, J. *et al.* Technical Targets for Hydrogen-Fueled Long-Haul Tractor-Trailer Trucks. Tech. Rep. 19006, Department of Energy (2019). URL https://www.hydrogen.energy.gov/docs/hydrogenprogramlibraries/pdfs/19006_hydrogen_class8_long_haul_truck_targets.pdf.

[120] Tamarona, P. B., Pecnik, R. & Ramdin, M. Viability assessment of large-scale Claude cycle hydrogen liquefaction: A study on technical and economic perspective. *International Journal of Hydrogen Energy* **77**, 383–396 (2024). https://doi.org/10.1016/j.ijhydene.2024.06.021 .

[121] Connelly, E., Penev, M., Elgowainy, A. & Hunter, C. Current Status of Hydrogen Liquefaction Costs. Tech. Rep. 19001, Department of Energy (2019). URL https://www.hydrogen.energy.gov/docs/hydrogenprogramlibraries/pdfs/19001_hydrogen_liquefaction_costs.pdf.

[122] Mendoza-Moreno, P. V., Fulham, G. J. & Marek, E. J. Harnessing the enigmatic ortho-para isomeric conversion for energy-efficient and low-carbon production of liquid hydrogen. *Cell Reports Sustainability* **1** (11), 100243 (2024). https://doi.org/10.1016/j.crsus.2024.100243 .

[123] Nejat Veziroglu, T., Sherif, S. A. & Barbir, F. in *CHAPTER 7 - Hydrogen Energy Solutions* (eds Agardy, F. J. & Nemerow, N. L.) *Environmental Solutions* 143–180 (Academic Press, Burlington, 2005). URL https://www.sciencedirect.com/science/article/pii/B9780120884414500083.

[124] Ewe, H. H. & Selbach, H.-J. in *The Storage of Hydrogen* (ed.Justi, E. W.) *A Solar—Hydrogen Energy System* 243–263 (Springer US, Boston, MA, 1987). URL https://doi.org/10.1007/978-1-4613-1781-4_11.

[125] Bhouri, M., Bürger, I. & Linder, M. Feasibility analysis of a novel solid-state H2 storage reactor concept based on thermochemical heat storage: MgH2 and Mg(OH)2 as reference materials. *International Journal of Hydrogen Energy* **41** (45), 20549–20561 (2016). https://doi.org/10.1016/J.IJHYDENE.2016.09.125 .

[126] Usman, M. R. Hydrogen storage methods: Review and current status. *Renewable and Sustainable Energy Reviews* **167**, 112743 (2022). https://doi.org/10.1016/j.rser.2022.112743 .

[127] Barrie, P. J. JavaScript Programs To Calculate Thermodynamic Properties Using Cubic Equations of State. *Journal of Chemical Education* **82** (6), 958 (2005). https://doi.org/10.1021/ed082p958 .
