# Supplementary material for: Thermally coupled solid hydrogen storage and carbon capture for balancing intermittent renewable energy
Source: Nat Commun. 2026 Apr 21;17:5514. doi: 10.1038/s41467-026-72035-1 (PMC13287580; doi:10.1038/s41467-026-72035-1)
Supplement: Supplementary file 2 — Transparent Peer Review file [file 41467_2026_72035_MOESM2_ESM.pdf]

nature portfolio

Peer Review File

Thermally coupled solid hydrogen storage and carbon capture for balancing intermittent renewable energy

Corresponding Author: Dr Binjian Nie

This file contains all reviewer reports in order by version, followed by all author rebuttals in order by version.

Version 0:

Reviewer comments:

Reviewer #1

(Remarks to the Author)
The manuscript provides a detailed study on a novel system that thermally couples hydrogen storage (solid state) and carbon capture (point source) to balance the intermittent wind power. Experiments were firstly conducted to obtain performance parameters for hydrogen storage in Mg alloy and carbon capture in Mg looping. Then system efficiency and long-term operation (daily) were modelled. The idea is new and interesting, and the analyses are in-depth. The challenges and limitations of this work were also discussed in the manuscript. Here are some comments to improve the paper.
1. The process diagram and operating conditions were in the supporting documents. Therefore, it is quite hard to get this new idea clearly while reading the main text. It would be beneficial for the readers if some of these key operational concepts can be clearly identified at the very beginning of the paper. For example, the hydrogen reactor is at a constant pressure and temperature, the carbonation reactor has a pressure-swing at a constant temperature.
2. In figure 2 (d), it seems that CO_2 release is already achieved at the end of the carbonation step, which is very confusing. Also, these two steps are not corresponding to the actual operation of the reactor (i.e., the actual reactor is with pressure swing at a constant temperature). Therefore, I suggest the authors clarify this more, or rename them.
3. Also, in figure 2(d), the difference between the experimental measurement and the theoretical maximum (how was this obtained? At which temperature?) is huge. The reason, i.e., mass-transfer limitation, may not be the only cause.
4. In figure 2 (c), the pressure is swinging (pH_2 = 20 bar for adsorption, while helium sweeping for desorption (also at 20 bar?). However, in the system modelling, the pressure is at constant (pH_2 = 4.5 bar for adsorption, and pH_2 = 4.5 bar for desorption, although temperatures are slightly different due to heat integration). So what is the driving force for hydrogen desorption in the system?
5. On top of page 10, the overall efficiency was not shown in Figure 1 (c). Please update this.
6. At the bottom of page 12, the carbon intensity was discussed. Please briefly explain the scope of this carbon intensity to help readers to understand the discussion better. I know that this is explained in detail in the supporting information.
7. It would be nice to report the flow rates of the feed streams and some important streams in the process flow diagram (Figure S3). Perhaps show the base case study flow rates corresponding to the Sankey diagram in Figure 3 in a table in the supporting information.
8. The Introduction states, "In order to balance daily and seasonal variation in electricity supply and demand, medium- (>1 week) to long-duration (>1 month) energy storage is required." Why is short-term energy storage deemed less critical in this context? Can the system designed by the authors effectively address short-term intermittency issues?
9. The authors should provide justification for the conversion efficiencies (e.g., ηRT and $\eta store$) adopted in the Sankey diagram to clarify the rationale behind their assumptions.
10. In the "System Design and Round-Trip Efficiency" section, the authors note, "Possibly counter-intuitively, the round-trip efficiency of the system was maximized by allocating a fraction of the input electricity to calcine the TCES material via electrical heating (here, 256 kWh), rather than directing all of the energy to electrolysis." It would be beneficial to explain the underlying logic behind this counter-intuitive observation.
11. The modeling is based on both optimistic and pessimistic assumptions. Out of curiosity, how would the results be affected if purely theoretical values were used instead?

(Remarks on code availability)

Reviewer #2

(Remarks to the Author)
Abstract and Introduction
1. The adsorption and desorption rates of solid-state hydrogen storage materials are typically slow. How do the authors propose to integrate this relatively sluggish subsystem with the dynamic operation of a wind turbine and electrolyzer?
2. In Section S2 of the Supplementary Information, you mention that the listed studies represent different approaches and perspectives. It would be beneficial to include an additional column summarizing the categories or distinguishing features of the studies in Tables S1 and S2. The current tables are difficult to interpret.
3. On page 4, the discussion of the combined solid-state hydrogen storage and heat recovery should be expanded to include the design considerations of a hydrogen storage reactor. As noted in recent literature, reactor geometry optimization of metal hydrides can significantly enhance heat transfer performance.
4. In Figure 1(c), how are uniform heat transfer, hydrogen storage, and carbon storage modeled within each subsystem? Moreover, how is the transition between carbon release during hydrogen storage and carbon capture during hydrogen production represented?
5. In Figure 1(d), which component provides the heat in the 200–325 °C range? Alternatively, do both subsystems operate only within the overlapping temperature window? Have you considered system behavior during hot and cold start-up conditions?
6. The operating temperature of the PEM electrolyzer appears too low for efficient thermal integration. Please clarify.
7. In Figure 1(e), heat losses are inevitable during inter-system heat exchange. Is there an auxiliary heat source to compensate for these losses and ensure the required hydrogen production rate? Furthermore, did you assume complete absorption of hydrogen within the system?
Results and Discussion
9. On page 9, you report that the material absorbs 5.6 wt% H_2 in approximately 10 minutes. However, the hydrogen flow rate from the PEM electrolyzer exceeds this value. What happens to the excess hydrogen? Given the absorption time of 10 minutes and desorption time of 30 minutes, how is the system operated during the remaining periods of each hourly interval?
10. How are the experimental results extrapolated or scaled up for system-level applications?
11. On page 12, the comparison between your proposed system and the current UK electricity mix seems inappropriate. The proposed system is nearly 100% renewable with limited gas-turbine backup, while the grid mix includes fossil contributions. A fairer comparison would be against other renewable-based energy storage systems under equivalent boundary conditions.
12. In Figure 5, when the overcapacity factor increases, does the capacity of the PEM electrolyzer change proportionally? The relative capacities of the wind turbine, electrolyzer, hydrogen storage, and fuel cell will strongly influence the results. Please clarify whether this has been accounted for.
13. The results shown in Figure 7 appear deterministic. The LCOE should be expressed as a range reflecting data and parameter uncertainties.
Methods
14. In the experimental methods, please specify the temperature ramp-up and ramp-down rates. Were these dynamic parameters also included in the simulation? Which experimental parameters were used to calibrate or validate the model?
15. In Figure S2, what occurs if the system experiences unresolved surplus or deficit conditions? Also, where is the gas turbine integrated, as mentioned in Figure 1?
16. In Figure S3, what are the pressure ratios of the multi-stage compressors for the flue gas and CO2 streams? Given that a typical air compressor has a pressure ratio of about 5, one stage might be sufficient for the flue gas, but four to five stages could be required for CO2 compression.
17. The system includes numerous heat exchangers (e.g., HX1, HX4, HX6, HX7). Is there a heat exchange network design underlying these connections? For instance, what is the rationale for including both HX6 and HX7, and how were the interconnections determined?
18. For reactors R1 and R2, if both are maintained near 350 °C, additional thermal losses and reduced adsorption capacity should be expected. What is the rationale for selecting this temperature level? Under such conditions, neither system may achieve optimal adsorption performance.
19. Did you assume that R1 absorbs 100% of the hydrogen produced by the electrolyzer? Please justify whether this assumption is realistic.
20. What efficiencies were assumed for the electrolyzer and fuel cell? Please also provide a reference supporting the assumed six-year lifetime. Some numbers are missing in the SI.
21. In Section S4, several numerical values are presented without references, and some assumptions appear deterministic. Please provide citations for all key parameters and clarify the basis for the assumed values.
22. The reference year should be included in Table S5, since inflation affects the LCOE calculations.
23. The parameters in Table S5 appear to be sourced from different references. How were data uncertainties treated? How did you define the base, optimistic, and pessimistic scenarios? The lifetimes and degradation or make-up rates of the hydrogen storage alloy and magnesium carbonate should also be considered.

(Remarks on code availability)

Reviewer #3

(Remarks to the Author)
I co-reviewed this manuscript with one of the reviewers who provided the listed reports. This is part of the Nature Communications initiative to facilitate training in peer review and to provide appropriate recognition for Early Career

Researchers who co-review manuscripts.

(Remarks on code availability)

Reviewer #4

(Remarks to the Author)
The authors present a techno-economic study of a coupled system that combines Mg-based solid hydrogen storage with a magnesium-looping thermo-chemical energy storage / carbon-capture cycle (MgO ↔ MgCO3). The core innovation is the use of waste heat from the solid-state hydrogen storage reaction to drive Magnesium looping as a secondary process. This leads to a complex interconnected system for energy storage and carbon removal.

The study presents the technoeconomic assessments of a variety of technical scenarios based on a model of the energy flow.

The work supports the claims. No additional evidence needed.

There seems to be no flaws in the data analysis. The methods are sound and described in detail. Computational routines and data is made available.

No revision is needed. Maybe the authors could consider these remarks and possibly add some clarifictions:

A turbomachinery (compression/expansion) path is coupled with an electrolyser/fuel cell path. This might lead to challenges in the engineering implementation. Design, scale up, longterm attrition of the reactors will also lead to engineering challenges.

Is everything needed available on site, e.g. flue gas and if necessary energy (i.e. for Dunkelflaute) ? What is the scenario/ use case of the proposed system: As an add on to power stations using the power from the grid, as an add on to windparks as long term energy storage ? Is the proposed system rather an enhanced hydrogen energy storage or rather an enhanced carbon capture from flue gas ? How many such systems /what capacity could be deployed i.e. in the UK and what would be the expected benefit ?

Comparison of costs in Figure 8. Maybe include an uncertainty margin for the estimates (especially for the new technologies) unsicherheitsspanne

Caption Figure 2: Define PCT and TGA

Figure 7. Numbers in images are barely readable

(Remarks on code availability)
Brief overview over stored files.
No time to run and check.

Version 1:

Reviewer comments:

Reviewer #1

(Remarks to the Author)
The revised manuscript has addressed my comments and its quality has been improved.

(Remarks on code availability)

Reviewer #2

(Remarks to the Author)
Thank you for your detailed response. The questions have been thoroughly addressed, and the manuscript has significantly improved in clarity.
I have only one minor comment: Figure 9 could be moved to the Supporting Information (SI) because it is less visually compelling than the other figures. The overall concept is already clearly illustrated in Figure 1. In this context, the detailed process design shown in Figure 9 appears less essential to the main narrative.
Regarding the Graphical Abstract, I suggest refining the right-hand side. Instead of presenting three separate plots, it may be more effective to explicitly highlight the key advantages of integrating solid hydrogen storage with the carbon capture loop,

as these represent the core insights conveyed by those three figures. Emphasizing this integration would make the central contribution clearer and more impactful.

(Remarks on code availability)

Reviewer #3

(Remarks to the Author)
I co-reviewed this manuscript with one of the reviewers who provided the listed reports. This is part of the Nature Communications initiative to facilitate training in peer review and to provide appropriate recognition for Early Career Researchers who co-review manuscripts.

(Remarks on code availability)

Reviewer #4

(Remarks to the Author)
All issues of initial review are addressesd.

(Remarks on code availability)
Lack of time.

Open Access This Peer Review File is licensed under a Creative Commons Attribution 4.0 International License, which permits use, sharing, adaptation, distribution and reproduction in any medium or format, as long as you give appropriate credit to the original author(s) and the source, provide a link to the Creative Commons license, and indicate if changes were made.
In cases where reviewers are anonymous, credit should be given to 'Anonymous Referee' and the source.
The images or other third party material in this Peer Review File are included in the article’s Creative Commons license, unless indicated otherwise in a credit line to the material. If material is not included in the article’s Creative Commons license and your intended use is not permitted by statutory regulation or exceeds the permitted use, you will need to obtain permission directly from the copyright holder.
To view a copy of this license, visit https://creativecommons.org/licenses/by/4.0/

To the reviewers,
On behalf of our co-authors, we thank you for your helpful feedback and suggestions on our manuscript. We have revised the manuscript as requested, with a list of our modifications in response to each comment given below. Where multiple reviewers have addressed the same issue, we have included the revision under each comment. Some similar comments on related issues by the same reviewer have been grouped and addressed together in the manuscript.

Responses to reviewers and changes to the manuscript are given in red. Extracts from the revised manuscript are given in *italics.*

We have also uploaded copies of the manuscript with changes marked, to aid identifying sections that we have altered. Modified code has been uploaded to the Code Ocean repository (DOI 10.24433/CO.5706678.v3).

We thank you again for considering our manuscript, and look forward to hearing any further feedback.

Yours sincerely,
Binjian Nie and Alexander Harrison

REVIEWER COMMENTS

Reviewer #1 (Remarks to the Author):

The manuscript provides a detailed study on a novel system that thermally couples hydrogen storage (solid state) and carbon capture (point source) to balance the intermittent wind power. Experiments were firstly conducted to obtain performance parameters for hydrogen storage in Mg alloy and carbon capture in Mg looping. Then system efficiency and long-term operation (daily) were modelled. The idea is new and interesting, and the analyses are in-depth. The challenges and limitations of this work were also discussed in the manuscript. Here are some comments to improve the paper.

1. The process diagram and operating conditions were in the supporting documents. Therefore, it is quite hard to get this new idea clearly while reading the main text. It would be beneficial for the readers if some of these key operational concepts can be clearly identified at the very beginning of the paper. For example, the hydrogen reactor is at a constant pressure and temperature, the carbonation reactor has a pressure-swing at a constant temperature.

In response to this and subsequent comments, we have moved the process flow diagram from the Supplementary Information (SI) to the main manuscript (Methods

section, Figure 9). We have also adjusted Fig. 1e to include a brief summary of the process conditions

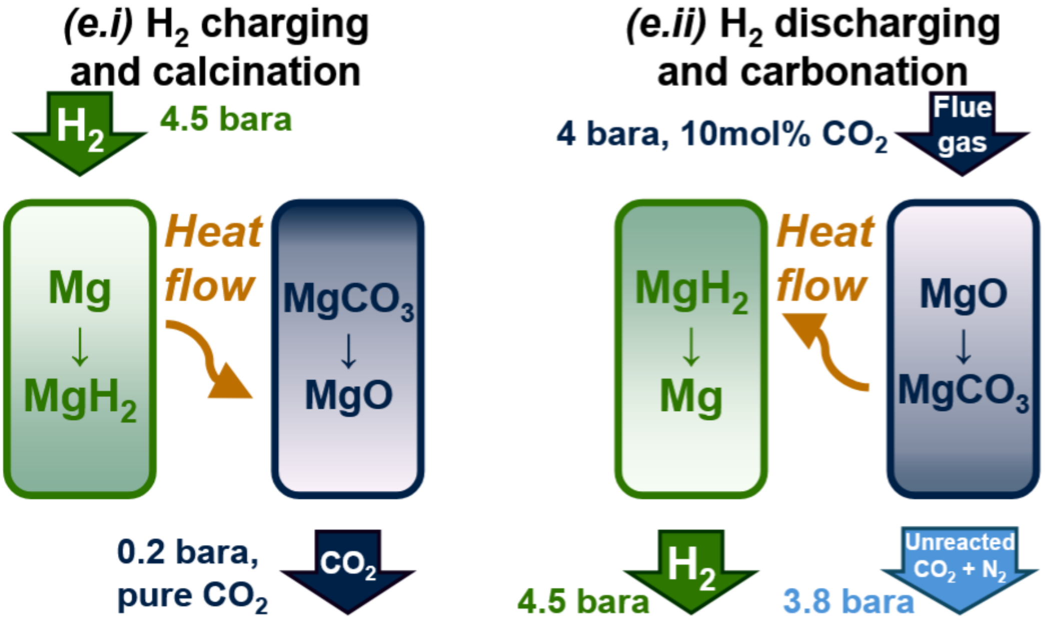

and added the following comment to the figure caption:

"Stream pressures shown correspond to operation at a nominal setpoint of 350°C, with a pressure swing between carbonation and calcination."

2. In figure 2 (d), it seems that CO2 release is already achieved at the end of the carbonation step, which is very confusing. Also, these two steps are not corresponding to the actual operation of the reactor (i.e., the actual reactor is with pressure swing at a constant temperature). Therefore, I suggest the authors clarify this more, or rename them.

We regret any confusion caused by the presentation of the carbonation measurements. The purpose of heating the sample of MgO-based sorbent in 0.33 bara CO2 was to determine the equilibrium temperature T_eq, at which the carbonation reaction MgO + CO2 -> MgCO3 stops, and the thermal decomposition reaction MgCO3 -> MgO + CO2 begins, in order to compare the measured value (370C) with the theoretical calculated value from the thermodynamic database used (FactPS, which predicted 365C, hence in reasonably good agreement). The subsequent cooling step in inert gas (nominal pCO2 = 10^-5 bara) was performed to remove any residual carbonate prior to the next heating cycle. We have adjusted the figure and caption, and associated text, as follows:

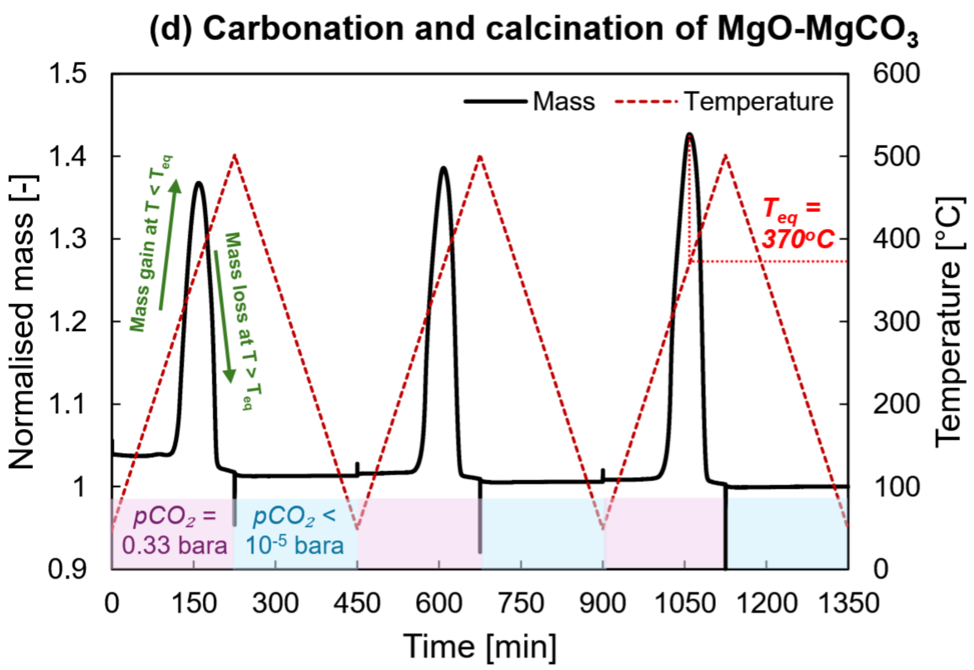

Caption: "Construction lines indicate the estimation of the equilibrium temperature (Teq) at pCO2 = 0.33 bara from the maximum mass gain; at temperatures exceeding Teq , the sample loses mass from carbonate decomposition."

Text: "Experimental measurements of hydrogen and CO2 sorption, shown in Fig. 2, were performed to estimate realistic maximum capacities of Mg and MgO-based sorbent materials respectively, and to validate the estimated material properties from thermodynamic calculations … Once the equilibrium temperature at pCO2 = 0.33 bara was exceeded, the sample then began to decarbonate at 370±1◦C, slightly higher than the theoretical equilibrium temperature of 365◦C. "

3. Also, in figure 2(d), the difference between the experimental measurement and the theoretical maximum (how was this obtained? At which temperature?) is huge. The reason, i.e., mass-transfer limitation, may not be the only cause.

To clarify, the theoretical maximum CO2 absorption corresponds to the theoretical mass gain determined from the reaction stoichiometry MgO + CO2 -> MgCO3 *i.e.* assuming full conversion with one mole of MgO absorbing one mole of CO2. In practice, for particulate magnesium and calcium based sorbents, the theoretical maximum is rarely achievable, as a result of the volume expansion during carbonation forming an impermeable "shell" of carbonate around the reactive oxide core. We have adjusted the text to briefly describe this phenomenon.

"The theoretical maximum CO2 uptake for complete conversion of MgO → MgCO3

is 87wt% (determined from reaction stoichiometry), however in practice sorbent rarely reach the theoretical maximum as a result of formation of an impermeable carbonate shell around the particles from volume expansion during carbonation, inhibiting inward CO2 transport [49]. Nevertheless, the measured CO2 capacity of 39wt% was
similar to reported experimental values [38, 40] of c. 40wt%. Therefore, for the pessimistic case a CO2 capacity of 35wt% was assumed, for the base case the reported value from literature of around 40wt% was used [40], and for the optimistic case, a higher value of 50wt% was used [38], allowing for improvements in sorbent design and composition to mitigate mass transfer limitation."

4. In figure 2 (c), the pressure is swinging (pH2 = 20 bar for adsorption, while helium sweeping for desorption (also at 20 bar?). However, in the system modelling, the pressure is at constant (pH2 = 4.5 bar for adsorption, and pH2 = 4.5 bar for desorption, although temperatures are slightly different due to heat integration). So what is the driving force for hydrogen desorption in the system?

We thank the reviewer for this observation. To clarify, the purpose of the cyclic H2-He experiments over the Mg-based material was to determine the maximum hydrogen uptake capacity of the sorbent, and to verify that the capacity did not meaningfully change over repeated cycles. For experimental convenience, we performed the hydrogen sorption and desorption isothermally in a batch reactor at 350C and a constant total pressure (20 bara). In order to induce hydrogen desorption, we opened the valve to the reactor, and displaced the gas contents using He to lower pH2 to near zero. In a practical process (*i.e.* as in the modelling section), inert gas would not be used to induce desorption (as this would dilute the hydrogen product): instead, a 5C temperature swing between hydrogenation and dehydrogenation was used, as described later in the manuscript. We have adjusted Fig. 2c and the accompanying text to clarify this point.

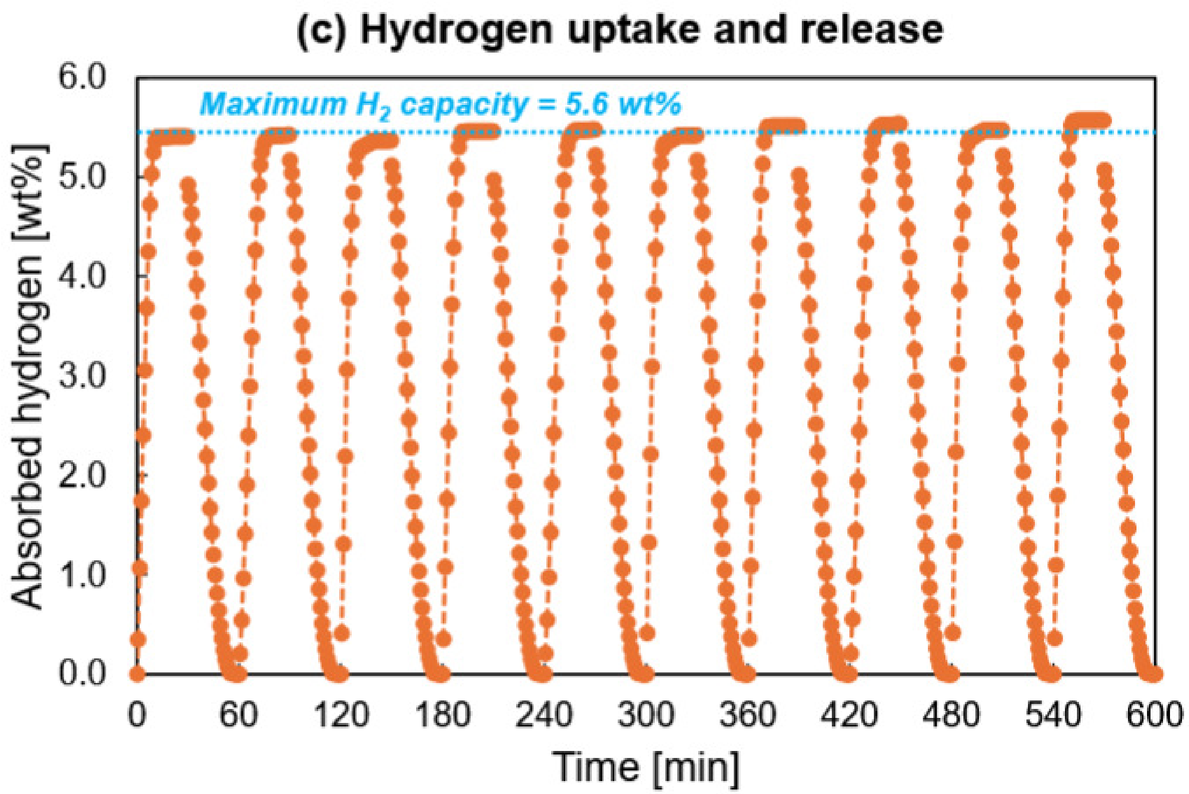

Caption: *"... (c) ten cycles of hydrogenation (pH2 = 20 bara) and dehydrogenation in He (20 bara total pressure, pH2 = 0 bara) at 350◦C for the Mg-based material prepared..."*

Text: *"Figure 2c shows ten cycles of hydrogen uptake at pH2 = 20 bara and release for the Mg-based alloy at 350◦C in a batch reactor, with the material absorbing 5.6±0.1 wt% H2, with no reduction in H2 capacity over 10 repeated cycles. Helium gas was used to lower the pH2 during desorption by displacing hydrogen gas; in a real hydrogen storage process, a temperature swing would be applied to induce desorption to avoid diluting the hydrogen produced [39]."*

5. On top of page 10, the overall efficiency was not shown in Figure 1 (c). Please update this.

We apologise for any confusion, as the sentence referred to was intended to introduce the methods used to estimate efficiency, rather than to indicate that the efficiency was shown in Fig. 1c. We have rephrased the sentence at the top of page 10 to remove the ambiguity as follows:

"For the storage system shown in Fig. 1c, the overall efficiency is determined by the electrical efficiency of the electrolyser and fuel cell for converting electricity into hydrogen..."

and have added the relevant efficiency parameters to the Sankey diagram in Fig. 3.

6. At the bottom of page 12, the carbon intensity was discussed. Please briefly explain the scope of this carbon intensity to help readers to understand the discussion better. I know that this is explained in detail in the supporting information. To clarify the scope of emissions, we have added the following note:

"The effective carbon intensity of the generated electricity (defined as the net tailpipe CO2 operating emissions as a result of CCGT utilisation and CO2 capture from the TCES cycle)"

7. It would be nice to report the flow rates of the feed streams and some important streams in the process flow diagram (Figure S3). Perhaps show the base case study flow rates corresponding to the Sankey diagram in Figure 3 in a table in the supporting information.

We thank the reviewer for this suggestion: we have added the following table of key flows to the Supplementary Information (Table S7).

Table S7 Stream conditions for conversion of 1 MWh of electricity into stored hydrogen, and subsequent converstion back to electricity, as shown in the Sankey diagram in the main manuscript (Fig. 3). Estimated flow rates assume that energy conversion is spread over a 24 h modelling period, giving units of mass flow *per* hour *per* megawatt-hour of electricity input (kg $h^{-1}MWh_{in}^{-1}$).

Stream	Temperature (°C)	Pressure (bara)	Total mass flow (kg MWh_{in}^{-1})	Day-averaged mass flowrate (kg h^{-1} MWh_{in}^{-1})
		Energy storage		
Electrolyser water feed	25	1	144	6.0
Electrolyser hydrogen outlet	50	20	16	0.7
Electrolyser oxygen outlet	50	20	128	5.3
Hydrogenation reactor feed	352.5	4.5	16	0.7
CO_2 reactor outlet	347.5	0.2	353	14.7
High-pressure CO_2 to pipeline	25	150	353	14.7
		Energy release		
Flue gas input	110	1	4708	196.2
Compressed flue gas to carbonation reactor	352.5	4	4708	196.2
Unreacted reactor outlet (CO_2 + N_2)	352.5	3.8	4355	181.5
CO_2 depleted flue gas exhaust	190	1	4355	181.5
Hydrogen reactor outlet	347.5	4.5	16	0.7
Fuel cell waste water outlet	80	1	144	6.0

8. The Introduction states, "In order to balance daily and seasonal variation in electricity supply and demand, medium- (>1 week) to long-duration (>1 month) energy storage is required." Why is short-term energy storage deemed less critical in this context? Can the system designed by the authors effectively address short-term intermittency issues?

We thank the reviewer for this comment: short term energy storage is indeed important for managing short-term variation in wind power output (e.g. hour-to-hour), for which grid scale battery technologies are relatively mature in comparison to the large scale, long duration energy storage required to balance seasonal variation in wind power output. However, the energy density and cost per unit energy stored on current batteries are insufficient to be cost-effective for monthly or longer balancing (as shown in the techno-economic modelling section, Fig. 8). We have added the following comment to the introduction to clarify the role of short- and long-term energy storage in a renewable electricity grid system:

"Grid-scale batteries can effectively balance short term variation in renewable energy supply over the course of a day [14, 15] with a relatively fast response time, but have a maximum feasible discharge time of around 4 h per cell [16], and high cost per unit of energy stored [17]. Therefore, in order to balance daily and seasonal variation in electricity supply and demand (shown in the SI, Section S1, Fig. S1), medium- (>1 week) to long-duration (>1 month) energy storage is required [12, 13, 18], with hydrogen-based energy storage showing greater energy density than battery systems, and lower long-term storage costs [13, 19, 20]"

For our modelled system, a battery subsystem is used to 'smooth out' hourly variation in wind farm output over the course of each day, such that our modelled system only has to balance energy intermittency on daily or longer timescales (in practice, as mentioned in the discussion, the average duration of hydrogen storage in our system is of the order ~1 month). To justify the use of our proposed battery system for smoothing hourly variation, we have added the following commentary to the SI:

S4.7 Battery subsystem
A lithium-ion battery system was included as part of the process, in order to balance short-term fluctuations in power supply and energy demand (shorter than the modelling time step of 24 h), allowing the power input and output of the PEM electrolyser and fuel cell respectively to be averaged over each full day [20, 70]. The energy capacity of the batteries was scaled to equal 1/6 of average daily energy demand i.e. up to 4 h of continuous discharging, the Balancing intermittent wind energy using H_2 storage and CO_2 capture S33 maximum discharge time of a typical commercial grid-scale Li-ion battery unit

[71].

As battery storage levels were not modelled explicitly, a simplified assessment was performed to determine whether 4 h of short-term storage capacity (i.e. the maximum discharge time of a commercial grid-scale Li-ion battery unit) would be sufficient to smooth hourly variation in wind turbine output over 24 h, such that daily wind turbine output could be considered as a single parameter. For each day in the dataset for the onshore and offshore locations, the maximum cumulative deviation in wind output from the daily average was estimated (shown for an example day in Fig. S5a). Then, normalising the cumulative deviation with respect to average windfarm output, and scaling according to fOCP as defined in the main manuscript, the minimum number of hours of battery capacity to smooth windfarm output was estimated for each day, shown in Fig. S5b), giving an average necessary capacity of 2.3 ± 2.0 h and 2.7 ± 1.9 h for offshore and onshore windfarms respectively.

However, as shown by the tail of longer capacities , some days with unusual weather patterns might require greater short-term storage capacity for 'true' smooth operation, requiring access to either external grid battery storage or additional energy imports from gas turbines. However, on days with a net energy deficit from the windfarm, energy would also be entering the system throughout the day from use of stored hydrogen in the fuel cell, partially counterbalancing short-term deficits in available wind power (and vice versa on days with a net energy surplus, with excess power used to generate hydrogen in the electrolyser), thereby decreasing the minimum necessary short-term storage capacity. Hence, for the purposes of this study, the interplay and scheduling requirements between short- and long-term storage (batteries and hydrogen respectively) was not modelled. Therefore, 4 h battery capacity was deemed sufficient for rough cost estimation, while noting that greater battery capacities might be required for sites with rapid hourly fluctuations in capacity factor."

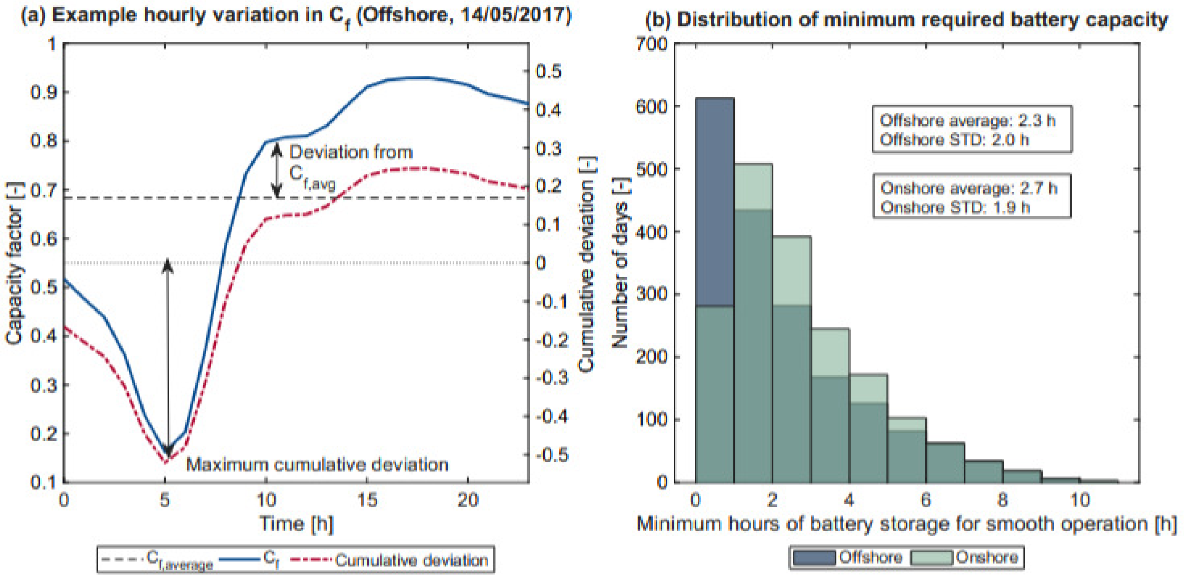

Figure S5 (a) Hourly variation in wind turbine capacity factor, and cumulative deviation from daily average capacity factor for a representative day (offshore North Sea wind farm, 14/05/2017), (b) histogram of estimated minimum battery capacity for each day in the dataset for onshore and offshore windfarms.

9. The authors should provide justification for the conversion efficiencies (e.g., ηRT and ηstore) adopted in the Sankey diagram to clarify the rationale behind their assumptions.

To clarify the scope of each efficiency value (ηRT, ηpassthrough and ηstore) we have revised the Sankey diagram, to show which energy flows are included and excluded within each efficiency metric (*i.e.* the round-trip efficiency corresponds to net electricity recoverable from the storage system per unit of electricity input, the passthrough efficiency corresponds to the ratio of fuel cell output to electrolyser input, and the storage efficiency corresponds to the ratio of the passthrough efficiency to the total round trip efficiency, corresponding to the fraction of total system losses that occur in the electrolyser and fuel cell. We have added the following note to the text to clarify the relationship between ηpassthrough and ηRT:

"From the definition of the passthrough efficiency, the maximum theoretical round-trip efficiency is given by ηRT = ηpassthrough = 23.4%, with full energy recovery from hydrogen storage."

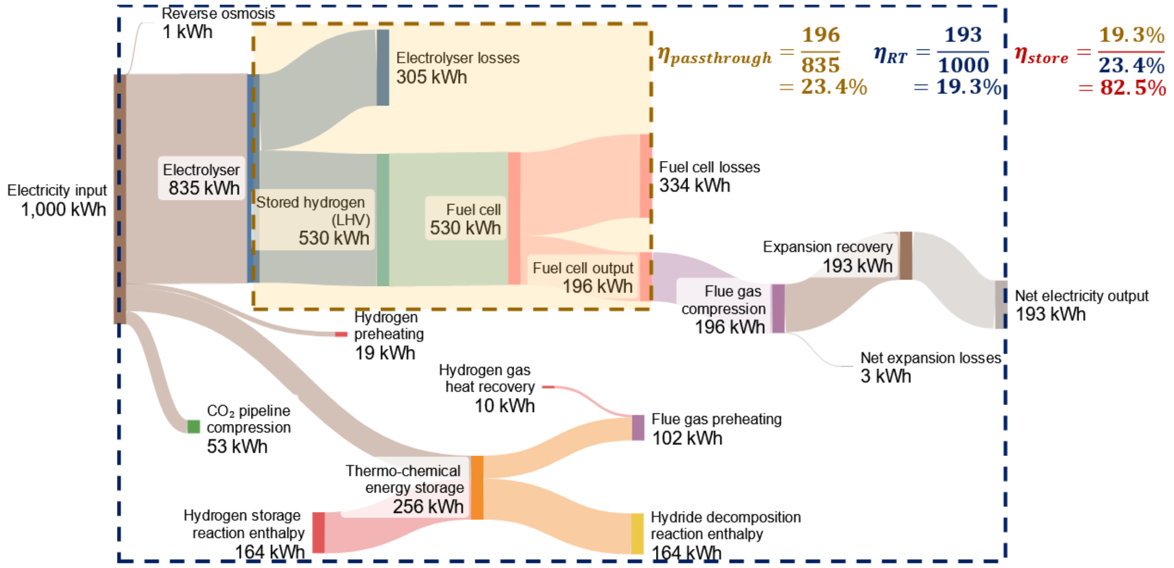

We have also added the following text in the accompanying paragraph next to the Sankey diagram, to justify the relatively low net energy loss associated with compression and expansion:

"The pressurised gas leaving the reactor (N2 and unreacted CO2) was then expanded to ambient pressure, generating electricity for delivery to users. As noted in the SI, Section S4.6, the gas leaving the reactor is at a higher temperature (here, 350◦C) than the flue gas compressor outlet (200◦C), allowing some conversion of heat to work via the Brayton cycle, and therefore giving relatively low net energy losses (here, 3 kWh) as a result of compression and expansion operations, despite irreversible losses in turbomachinery and consumption of gas in the carbonation reaction. Mass flowrates of process streams are given in the SI, Section S6, Table S7."

10. In the "System Design and Round-Trip Efficiency" section, the authors note, "Possibly counter-intuitively, the round-trip efficiency of the system was maximized by allocating a fraction of the input electricity to calcine the TCES material via electrical heating (here, 256 kWh), rather than directing all of the energy to electrolysis." It would be beneficial to explain the underlying logic behind this counter-intuitive observation.

In response to this comment, we have added a brief note to the main text explaining this phenomenon:

"...If all the available surplus power were directed towards electrolysis, a greater amount of hydrogen would be stored, however, as a result of energy losses in the electrolyser, less heat would be stored in the TCES material. Hence, during discharging, insufficient heat would be available from the TCES material to fully discharge the stored hydrogen, requiring the use of additional electrical heating at a point in the cycle where less energy is available in total (as the hydrogen is discharged during periods of net electricity deficit)."

11. The modeling is based on both optimistic and pessimistic assumptions. Out of curiosity, how would the results be affected if purely theoretical values were used instead?

Regarding the round trip efficiency, we have added the comment (mentioned above) discussing the maximum theoretical efficiency with perfectly efficient hydrogen conversion and no net turbomachinery losses.

"From the definition of the passthrough efficiency, the maximum theoretical round-trip efficiency is given by $\eta RT = \eta passthrough = 23.4\%$, with full energy recovery from hydrogen storage."

We also direct the reviewer to subsequent changes to the modelling approach discussed below, whereby for our techno-economic model, we use the base case assumptions for simple point-estimate comparisons between system configurations, then, perform Monte Carlo simulation by sampling parameters over the range between the pessimistic and optimistic values to estimate the overall distribution of plausible system costs (with the optimistic and pessimistic cases as lower and upper limits respectively).

Reviewer #2 (Remarks to the Author):
Reviewer #3 (Remarks to the Author):
I co-reviewed this manuscript with one of the reviewers who provided the listed reports. This is part of the Nature Communications initiative to facilitate training in peer review and to provide appropriate recognition for Early Career Researchers who co-review manuscripts.

Abstract and Introduction
1. The adsorption and desorption rates of solid-state hydrogen storage materials are typically slow. How do the authors propose to integrate this relatively sluggish subsystem with the dynamic operation of a wind turbine and electrolyzer?

As noted in response to comment 8 from Reviewer 1, for the purposes of our system model we assume that the battery storage system can be used to balance hourly variation in power supply from the windfarm, acting as a 'buffer' to minimise rapid changes in electrolyser power. Moreover, we assume that through scheduling power delivery to the electrolyser such that excess power is delivered continuously over the 24 h modelling step, compensating for the relatively slow rates of hydrogenation. However, as noted in the discussion, designing a control system for managing the several subprocesses within the system with relatively slow rates of reaction presents a considerable challenge for future work:

“Additionally, the system couples two relatively slow chemical reactions (hydrogen sorption and MgO carbonation) with turbomachinery for gas compression, each with different characteristic start-up and shut-down times, and with lag from turbomachinery introducing significant difficulties in process control, especially during the transition periods between carbon capture and CO2 release. While directly modelling the relative rates of each process was beyond the scope of this study, future work should consider the design of control systems able to allow the system to cope with varying load over time, incorporation of additional short-term electricity storage in the form of batteries, and short-term compressed gas storage using buffer tanks.”

2. In Section S2 of the Supplementary Information, you mention that the listed studies represent different approaches and perspectives. It would be beneficial to include an additional column summarizing the categories or distinguishing features of the studies in Tables S1 and S2. The current tables are difficult to interpret.
To aid interpretation of Tables S1 and S2, we have added a brief summary to indicate the topic of each paper to the first column *e.g.*

**Demonstration scale hydrogen storage reactor: Ren *et al.* (2023) [4]**	• Mobile pilot-scale H_2 storage system (designed and constructed with HyFun Energy Technology Co.) using pellets of Mg-Ni based alloy (*c.* total 1 t stored H_2, 14.4 t alloy, TRL 7) without heat recovery.	• Reactor compared with compressed H_2 for mobile applications, with *c.* 3× greater volumetric energy density.
	• Heat transfer using integrated tubular heat exchanger with heat transfer oil, heated using combustion of fuel oil.	• System able to charge or discharge within c.12 h, with estimated output of 1.6-3.2 MWh of electricity using a PEM fuel cell.
	• Use of heat recovery using molten salt PCMs, or MgO-$Mg(OH)_2$ TCES suggested for stationary applications, but no measured or calculated efficiencies reported.	• Stable hydrogen absorption and desorption for up to ~3,000 cycles.
**Experimental demonstration of coupled hydride storage with hydroxide TCES: Lutz *et al.* (2020) [5] and works cited therein**	• Bench-scale packed bed reactor experiments combining MgH_2 hydrogen storage (using 234 g of a Mg-based alloy with 10wt% Ni) with a concentric bed of MgO-$Mg(OH)_2$ TCES material (TRL 3-4).	• Steam pressure of 10 bar required at reactor operating temperature of 300°C (*i.e.* superheated).
	• Hydrogenation and dehydrogenation performed in separate steps, with radial temperature distribution through hydride and TCES beds monitored.	• Use of TCES material during hydrogen absorption resulted in *c.* 10°C lower temperature overshoot in external concentric bed, as a result of endothermic reaction $Mg(OH)_2 \rightarrow MgO + H_2O$.

3. On page 4, the discussion of the combined solid-state hydrogen storage and heat recovery should be expanded to include the design considerations of a hydrogen storage reactor. As noted in recent literature, reactor geometry optimization of metal hydrides can significantly enhance heat transfer performance.
In response to this suggestion, we have added the following brief commentary on geometric optimisation to the introduction:

"For practical reactor systems incorporating heat recovery from hydrogen storage, the geometric design of the reactor has a marked effect on heat transfer efficiency, with e.g. segmented beds [28], or optimised reactor aspect ratio [29] improving performance. However, for the purposes of system analysis, a 0-dimensional thermodynamic model provides useful insights into system behaviour and efficiency, allowing for future optimisation of both reactors, or

combining both hydrogen storage and TCES into a single vessel [34].”

4. In Figure 1(c), how are uniform heat transfer, hydrogen storage, and carbon storage modeled within each subsystem? Moreover, how is the transition between carbon release during hydrogen storage and carbon capture during hydrogen production represented?

As noted in response to the previous comment, the reactions as modelled here assume a 0-dimensional, thermodynamically limited process, and hence, heat and mass transfer were assumed to be uniform within each reactor. For the model, the system was assumed to switch between operating modes (hydrogen storage + calcination and hydrogen release + carbonation) within 24 h (i.e. one modelling time step), without modelling the transition explicitly. However, as noted in the discussion in response to comment 1, a detailed dynamic model of the system incorporating process control would need to manage these transition periods.

5. In Figure 1(d), which component provides the heat in the 200–325 °C range? Alternatively, do both subsystems operate only within the overlapping temperature window? Have you considered system behavior during hot and cold start-up conditions?
To address the point regarding the temperature range, we have modified Figure 1d to clarify that the purple shaded region corresponds to the temperature range 325C-415C where both reactions are feasible for the selected equipment, and added the following note to the figure caption.

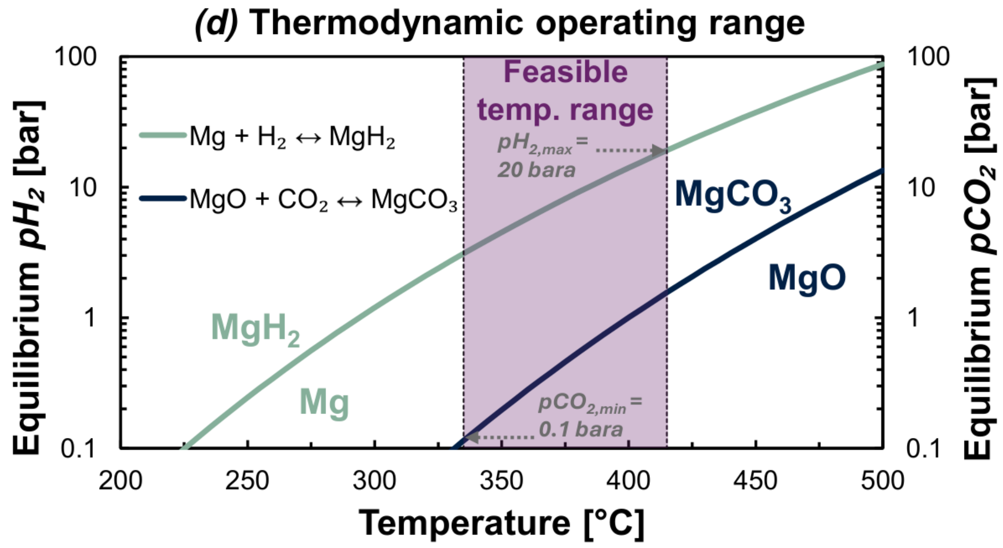

“...If the system were operated at a higher setpoint temperature (>415◦C), the equilibrium hydrogen pressure of MgH2 would exceed the output pressure of the PEM electrolyser, and would therefore require additional pre-compression of the H2

feed. Similarly, if the system were operated at a lower setpoint temperature (<335◦C), the equilibrium CO2 pressure during CO2 release from MgCO3 calcination would be lower than the partial pressure of CO2 in the flue gas feed)...”

As noted in response to a later comment, we did estimate system performance for temperatures below 335C, finding that the additional compression duty required to bring the CO2 released to pipeline pressure for sequestration decreased overall energy efficiency.

Regarding behaviour during start-up and shut-down, we assume that the system is kept at a consistent temperature throughout operation (*e.g.* 350C), and estimate the corresponding energy input required from electrical heater HX6 to compensate for ambient losses from both reactors. While lower thermal losses could be achieved by allowing the reactors to cool to ambient temperature during long periods of no hydrogen release or storage, the decision to shut-down or re-activate the system would require prediction of future weather conditions, and so was deemed beyond the scope of this study. Moreover, for the simulated systems, the longest periods of reactor inactivity in the 5-year modelling period were 27 and 16 days for on-shore or off-shore windfarms respectively (as shown in Tables S7 and S8), and therefore shutting down the system was not considered worthwhile. We have added a note to this effect to the Supplementary Information, Section S4.1:

“While lower thermal losses could potentially be achieved by allowing the reactors to cool to ambient temperature during long periods of no hydrogen release or storage, the decision to shut-down or re-activate the system would require daily prediction of future weather conditions, and so was deemed beyond the scope of this study. Moreover, for the simulated systems, the longest periods of reactor inactivity in the 5-year modelling period were 27 and 16 days for on-shore or off-shore windfarms respectively (as shown in Tables S7 and S8), and therefore shutting down the system was not considered worthwhile.”

6. The operating temperature of the PEM electrolyzer appears too low for efficient thermal integration. Please clarify.
This is correct - the PEM electrolyser and fuel cells were selected to allow for relatively rapid start-up and shut-down, such that the assumption that load could be managed over a 24 h modelling period would be justified. Therefore, the operating temperature of the PEM equipment was below the operating temperature of the hydrogen storage reactor, and so heat integration between the PEM equipment and the other streams was not attempted. Instead, the hydrogen released from the electrolyser was pre-heated to the reactor temperature using heat exchanger HX1 during hydrogen charging, and, heat was recovered from the hydrogen at ~350C released during discharging, using heat exchanger HX3 to pre-heat the CO2 feed to the carbonation reaction, thereby bringing the hydrogen to the fuel cell temperature.

Direct thermal integration between electrolysis and hydrogen storage has been investigated for other electrolyser types (e.g. solid oxide electrolysers in Wang *et al.,* 2024), but the relatively slow start-up and shut-down of higher temperature systems rendered them unsuitable for daily energy balancing as investigated here. We have added a brief note to this effect to the Supplementary Information, Section S4.1:

"As the operating temperatures of the PEM units were well below the temperatures of the reactors, the electrolyser and fuel cell were not heat-integrated with other units, in contrast with other studies modelling solid-oxide electrolysis [32], where waste heat from hydrogen storage was used to pre-heat the electrolyser feed."

7. In Figure 1(e), heat losses are inevitable during inter-system heat exchange. Is there an auxiliary heat source to compensate for these losses and ensure the required hydrogen production rate? Furthermore, did you assume complete absorption of hydrogen within the system?

Regarding heat losses, electric heating was used to provide supplementary heating to the system, and ensure an overall constant temperature was maintained. In practice, multiple electrical heaters located at various points in the process would be necessary in order to achieve fully isothermal operation (e.g. several heating coils connected to each reactor), but for ease of modelling, we considered the total electrical heating load as being supplied by a single unit, HX6, as shown in the revised process flow diagram (Fig. 9).

Regarding hydrogen absorption, we assumed that the system was limited by the equilibrium partial pressure of the hydride formation/decomposition reaction, and the experimentally-derived hydrogen capacity of the Mg-based materials, assuming that over the 24 hour modelling period, the system was able to approach thermodynamic equilibrium (De Rango *et al*, 2016), with some gaseous hydrogen remaining in the reactor headspace in order to maintain equilibrium. In practice, to achieve an additional driving force for absorption, the hydrogenation reaction could be operated above equilibrium pressure by using the electrolyser output directly (*i.e.* removing pressure regulation valve TV1). Hence, the round-trip efficiency parameters estimated here represent an upper-bound estimate for the case where thermodynamic equilibrium is achieved; if excess hydrogen generation were required to compensate for incomplete absorption, the effective round-trip efficiency would be decreased. We have added a brief note to this effect to the Supplementary Information, Section S4.3.

"Hydrogen absorption was assumed to be limited by the equilibrium partial pressure of the hydride formation reaction and the experimentally-derived hydrogen capacity of the Mg-based materials, assuming that over the 24 hour modelling period, the system was able to approach thermodynamic equilibrium [39], with some gaseous hydrogen remaining in the reactor headspace in order to maintain equilibrium.

Hence, the round-trip efficiency parameters estimated here represent an upper-bound estimate for the case where thermodynamic equilibrium is achieved; if excess hydrogen generation were required to compensate for incomplete absorption, the effective round-trip efficiency would be decreased. In practice, to achieve an additional driving force for absorption, the hydrogenation reaction could be operated above equilibrium pressure by using the electrolyser output directly (i.e. removing pressure regulation valve TV1)."

Results and Discussion

9. On page 9, you report that the material absorbs 5.6 wt% H_2 in approximately 10 minutes. However, the hydrogen flow rate from the PEM electrolyzer exceeds this value. What happens to the excess hydrogen? Given the absorption time of 10 minutes and desorption time of 30 minutes, how is the system operated during the remaining periods of each hourly interval?

10. How are the experimental results extrapolated or scaled up for system-level applications?

In response to comments 9 and 10, and a previous comment by another reviewer (Reviewer 1 Comment #4), we have added the following text to clarify that the experimental measurements reported in Fig. 2 were used solely to estimate the maximum H2/CO2 capacity of the Mg- and MgO-based materials respectively (used as input parameters for the model), and to verify that the assumed thermodynamic properties (i.e. equilibrium temperature of reaction) from literature databases were sufficiently accurate to use for estimating material behaviour. Hence, the absorption and desorption times used in experiments were arbitrary, and were selected for experimental convenience rather than real system operation (*e.g.* in real system operation, intermediate hydrogen buffer storage could be used to maintain the reactor gas headspace at equilibrium pressure until the absorption reaction had reached completion).

"Experimental measurements of hydrogen and CO2 sorption, shown in Fig. 2, were performed to estimate realistic maximum capacities of Mg and MgO-based sorbent materials respectively, and to validate the estimated material properties from thermodynamic calculations. … Figure 2c shows ten cycles of hydrogen uptake at pH2 = 20 bara and release for the Mg-based alloy at 350◦C in a batch reactor, with the material absorbing 5.6±0.1 wt% H2, with no reduction in H2 capacity over 10 repeated cycles. Helium gas was used to lower the pH2 during desorption by displacing hydrogen gas; in a real hydrogen storage process, a temperature swing would be applied to induce desorption to avoid diluting the hydrogen produced [39]."

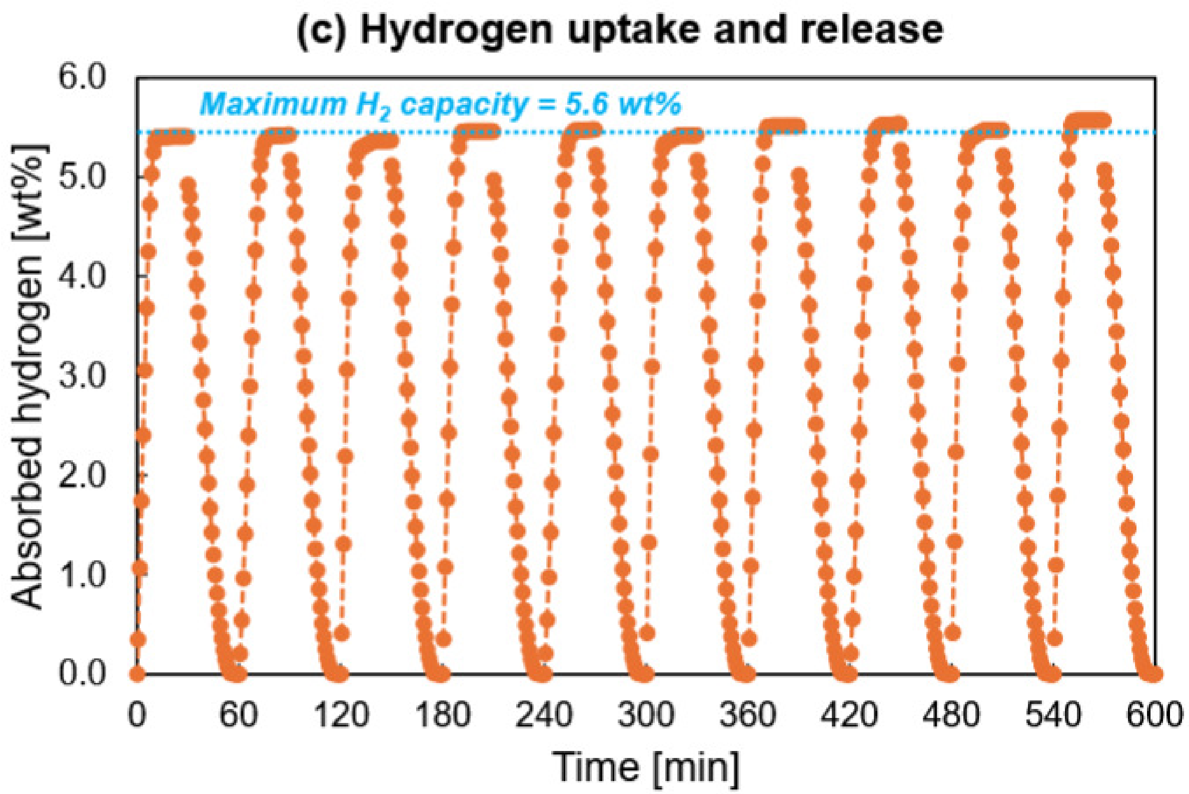

Caption: *"... (c) ten cycles of hydrogenation (pH2 = 20 bara) and dehydrogenation in He (20 bara total pressure, pH2 = 0 bara) at 350◦C for the Mg-based material prepared..."*

11. On page 12, the comparison between your proposed system and the current UK electricity mix seems inappropriate. The proposed system is nearly 100% renewable with limited gas-turbine backup, while the grid mix includes fossil contributions. A fairer comparison would be against other renewable-based energy storage systems under equivalent boundary conditions.

We thank the reviewer for this comment, and agree that a direct comparison between the proposed system and the current UK grid is unsuitable given the differing boundary conditions. We have removed this comparison, and added the following clarifying points regarding the system boundaries and use of back-up gas power in our system:

"The effective carbon intensity of the generated electricity (defined as the net tailpipe CO2 operating emissions as a result of CCGT utilisation and CO2 capture from the TCES cycle) is shown in Fig. S6c, with net CO2 capture during H2 storage (corresponding to the shaded blue regions), and net CO2 emissions as a result of electricity generation from backup gas turbines during periods of prolonged electricity deficit (corresponding to the shaded red regions). Across the five year modelling period, the system achieved an overall CO2 intensity of -7.2 kgCO2 per megawatt-hour of electricity supplied to users (i.e.

near net-zero emissions), within the operating boundaries of the process in Fig. 1c."

12. In Figure 5, when the overcapacity factor increases, does the capacity of the PEM electrolyzer change proportionally? The relative capacities of the wind turbine, electrolyzer, hydrogen storage, and fuel cell will strongly influence the results. Please clarify whether this has been accounted for.

Yes - for each simulated case, the capacities of the windfarm, electrolyser, fuel cell, and storage systems are dynamically adjusted based on the altered design parameters (windfarm overcapacity and hydrogen storage capacity). We have added the following explanation to the accompanying text next to Fig. 5.:

"To identify the effects of altering the system design parameters, we repeated the simulations for 121 combinations with 0-20 days worth of hydrogen storage capacity, and scaling the installed wind farm and process equipment according to overcapacity factors from 0-1.5 (where a factor of 0 means installing the wind farm capacity to exactly match the time-averaged electricity demand) … For each case, equipment sizing (e.g electrolyser and fuel cell capacity) were influenced by both overcapacity factor and hydrogen storage capacity, as both parameters affected the maximum available surplus energy that could be used in the electrolyser to produce hydrogen for storage, and, the maximum deficit between windfarm power output and user demand, determining the maximum required conversion of hydrogen into electricity in the fuel cell."

13. The results shown in Figure 7 appear deterministic. The LCOE should be expressed as a range reflecting data and parameter uncertainties.

We thank the reviewer for this suggestion and in response, have significantly reorganised our techno-economics and cost estimation section. Instead of using deterministic point-estimates for three representative cases (base, optimistic, pessimistic), for detailed estimation of LCOE and CAPEX/OPEX breakdowns, we have performed Monte Carlo simulation to estimate the uncertainty ranges for CAPEX/OPEX/LCOE, described in the SI, Section S7.1:

"S7.1 Sensitivity to input parameters: Monte Carlo simulation
To quantify the uncertainty in estimated CAPEX, OPEX, LCOE, and carbon intensity of electricity, Monte Carlo simulations were performed by sampling input values from the ranges reported in Tables 1 and S5. For each parameter, input values were sampled from a triangular distribution, with end points at the maximum and minimum values, and peak at the base value. For each simulation iteration, input values were sampled pseudo-randomly using the in-built rng function in MATLAB, using the 'twister' random number generation algorithm with an arbitrary 6-digit seed. The sensitivity of the Monte Carlo simulation to number of iterations and seed used is

shown in Fig. S9, indicating convergence at ≥5,000 iterations, and little sensitivity to input seed. For the simulations reported in Figs. 7 and 8, 10,000 iterations were used with seed '771923'.

The relative sensitivity of the model to each input parameter was roughly estimated by calculating the Spearman rank correlation coefficient [113] for each input variable, shown in Fig. S10. For both onshore and offshore system, CAPEX, OPEX, and LCOE showed the greatest sensitivity to assumed windfarm installation and operating costs, hydrogen storage alloy cost and lifetime, and PEM equipment installation cost and lifetime, in line with the CAPEX and OPEX compositions shown in Fig. 7c-f. Carbon intensity showed a strong correlation with assumed gas turbine efficiency and electrolyser efficiency, but no significant correlation with any other parameters"

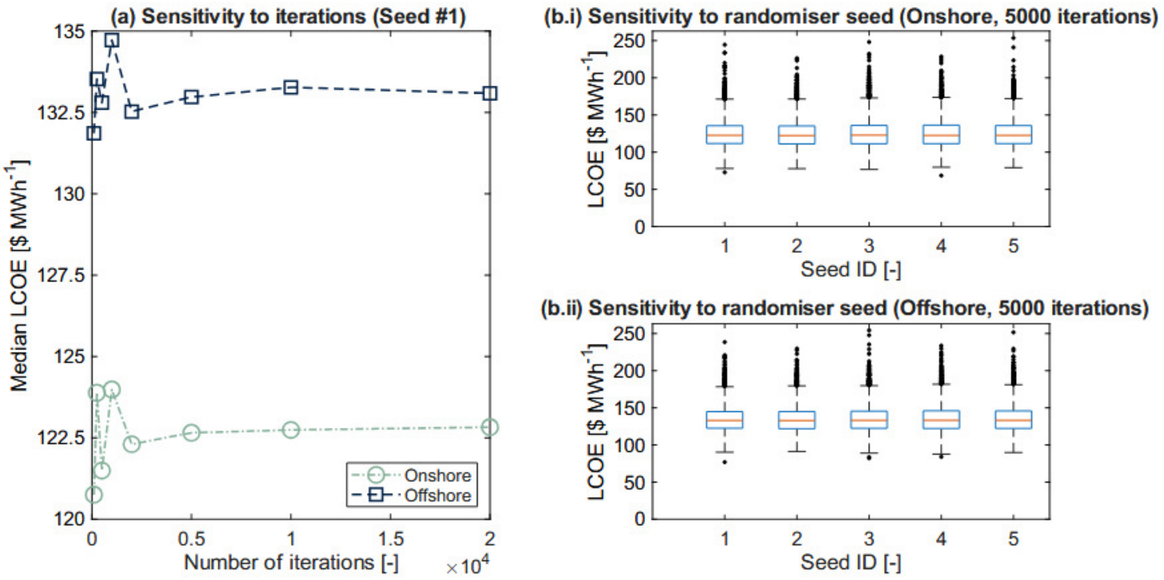

Figure S9 (a) Median estimated LCOE from Monte Carlo model as a function of number of model iterations (100-20,000 for onshore and offshore systems, showing convergence after *c.*5,000 iterations. (b) Distribution of estimated LCOE values over 5,000 model iterations for five different randomiser seeds, showing minimal difference in output for different seed inputs.

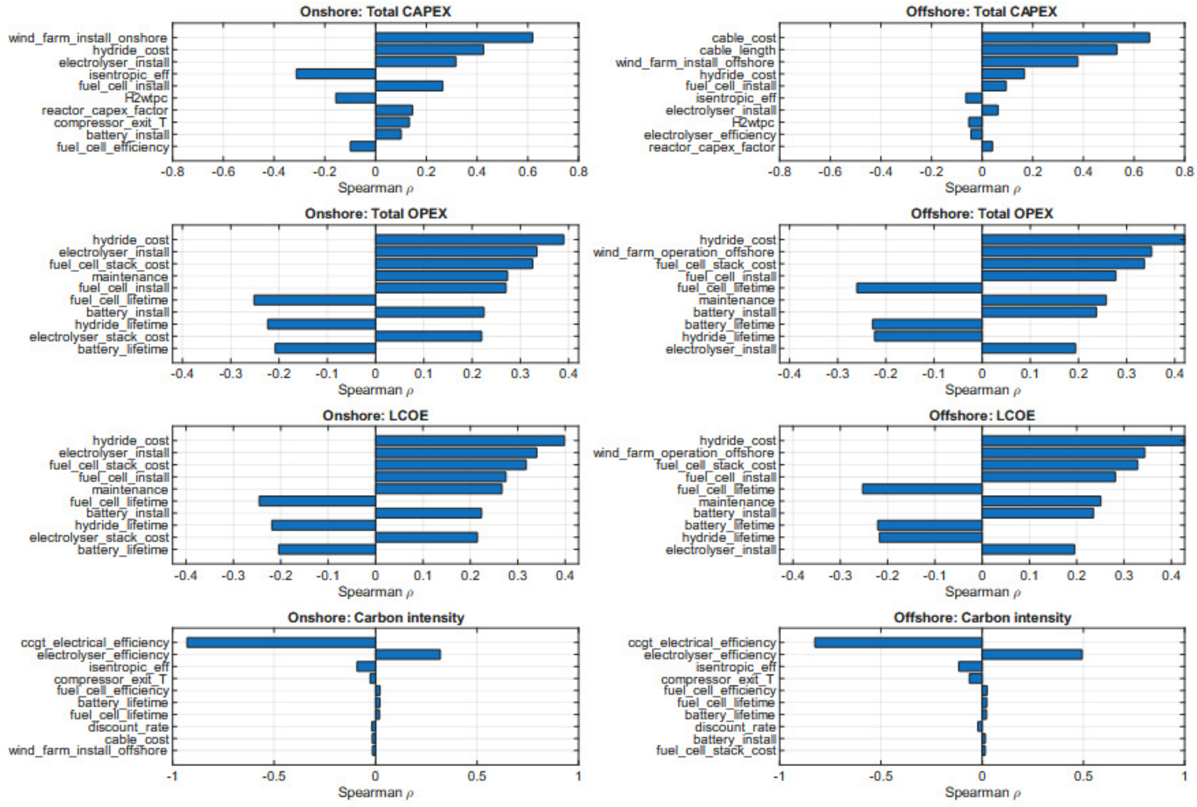

Figure S10 Tornado plots showing Spearman rank coefficients (ρ) for different model input parameters varied in the Monte Carlo model on CAPEX, OPEX, LCOE, and carbon intensity of generated electricity. A greater magnitude of ρ indicates a greater relative effect of each input parameter on the simulated output variable, with the sign indicating a positive or negative correlation.

We have also adjusted Figures 7 and 8b/c to reflect the estimated uncertainty ranges, and adjusted the reported values with uncertainty ranges in the accompanying text.

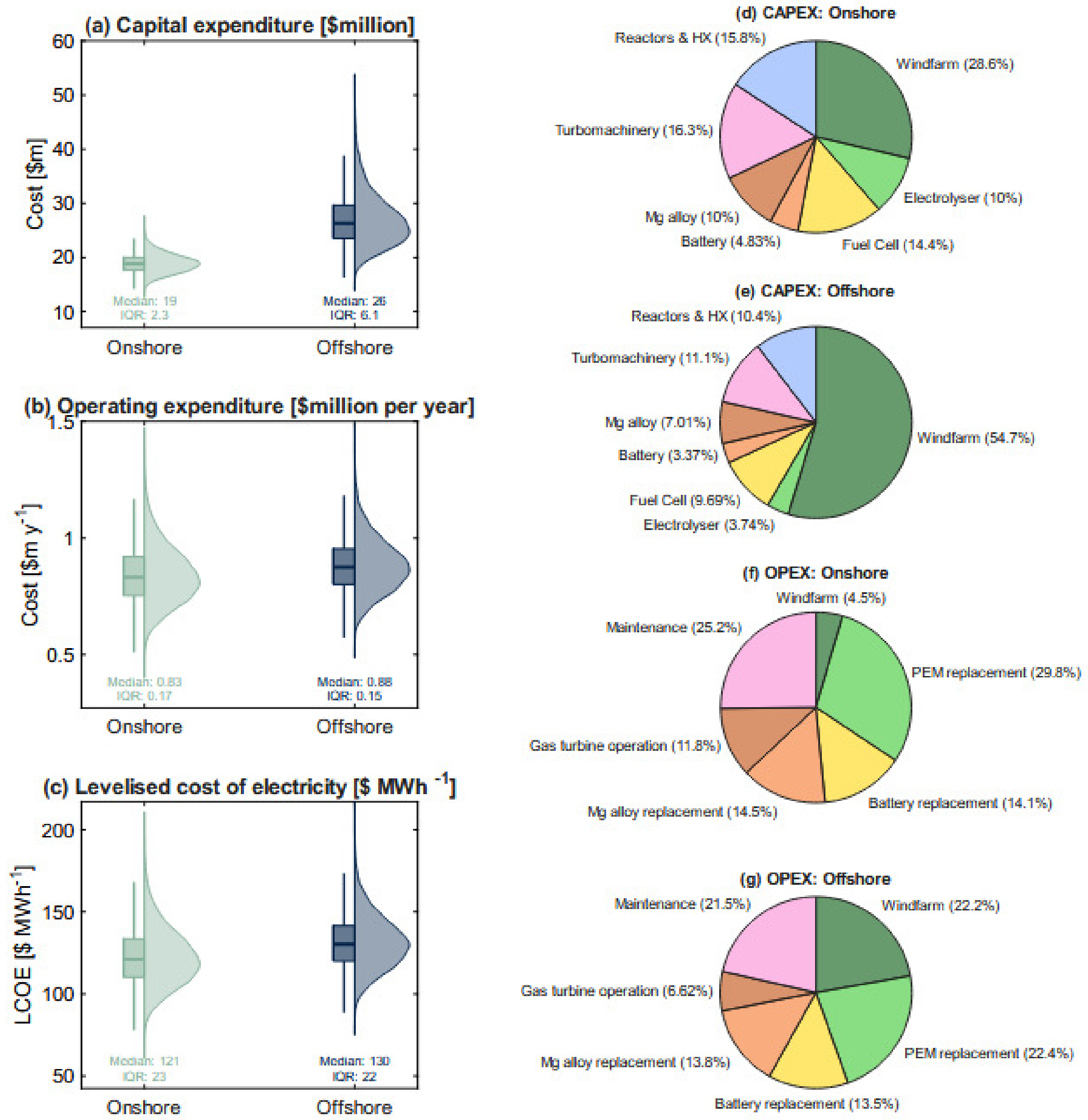

Figure 7 Distributions of estimated (a) capital expenditure (CAPEX), (b) operational expenditure (OPEX) and (c) levelised cost of electricity (LCOE) for onshore and offshore systems, both with $f_{OCP} = 0.3$ and 2 days of H_2 storage. Boxes show the interquartile range (IQR) with the median indicated by a horizontal line; whiskers extend to 1.5× the IQR. Violin widths represent the kernel density estimate (10,000 model iterations). Pie charts (d-g) show averaged distributions of CAPEX and OPEX for system costs corresponding to at least 1% of CAPEX or OPEX.

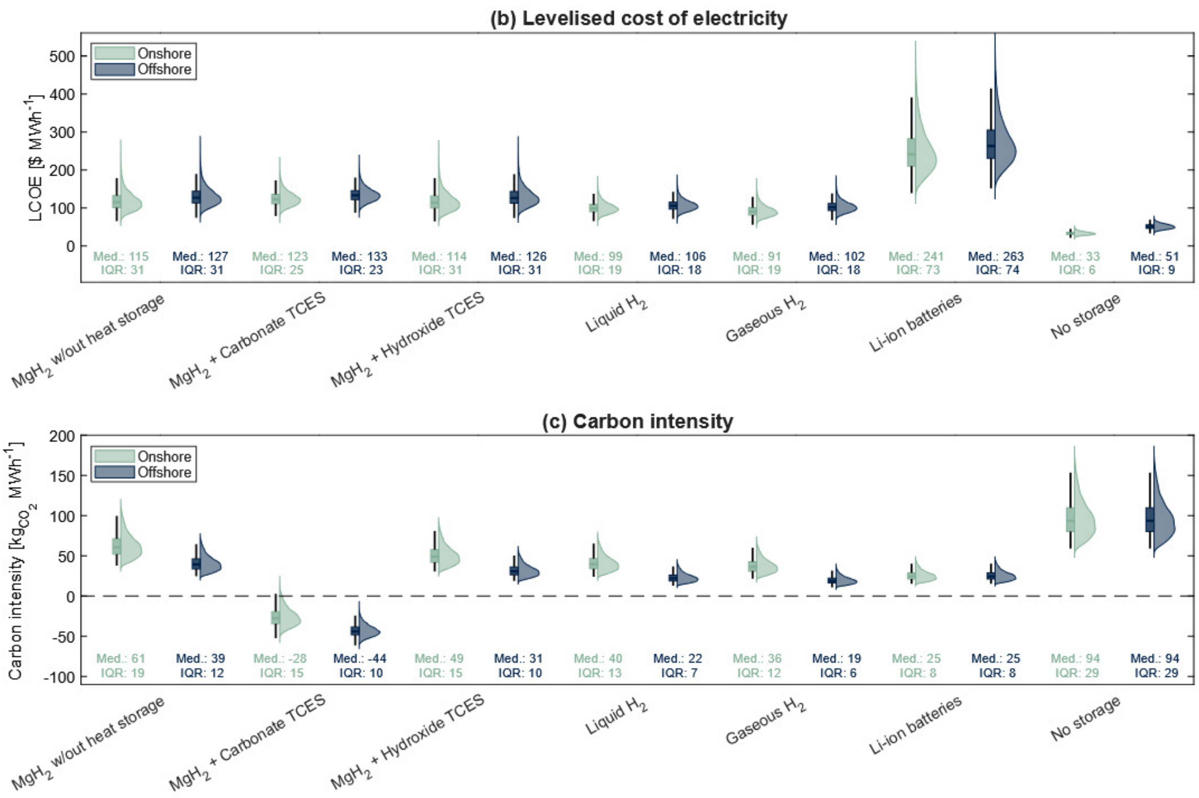

Figure 8 Comparison of **(a)** round trip energy efficiency (with the value for Li-ion batteries estimated from literature [56]) and storage efficiency as defined in Eqs. 5 and 7 **(b)** levelised cost of electricity and **(c)** carbon intensity of generated electricity for alternative system configurations. Cost and carbon intensity were estimated by Monte Carlo simulation for systems with $f_{OCP} = 0.3$ and 2 days of hydrogen or battery storage capacity as described in the SI, Section S7.1. Boxes in (b) and (c) show the interquartile range (IQR) with the median indicated by a horizontal line; whiskers extend to 1.5× the IQR. Violin widths in (b) and (c) represent the kernel density estimate (10,000 model iterations). In order to maintain a fair comparison between cases, electrical heating was assumed to be used for all MgH_2-based systems; alternative cases using different heating methods are discussed in the SI, Sections S4 and S8.

Methods

14. In the experimental methods, please specify the temperature ramp-up and ramp-down rates. Were these dynamic parameters also included in the simulation? Which experimental parameters were used to calibrate or validate the model?

In response to this comment, we have adjusted the methods section and figure caption for Figure 2 to explicitly state the ramp rates used:

"During carbonation, a sample (~20 mg) of MgO-based material was heated from 50◦C to 500◦C at 2◦C min−1 under flowing CO2 (50 mL min−1, BOC, 99.99+%), with purge and protective flow of N2 through the TGA chamber (each 50 mL min−1, BOC, 99.999+%), giving a nominal pCO2 of 0.33 bara. During calcination, the sample was cooled from 500◦C to 50◦C at -2◦C min−1 under N2 flow (50 mL min−1, nominal pCO2 < 10−5 bara)."

"(d) three carbonation-calcination cycles from 50-500◦C at a ramp rate of 2◦C min−1 for MgO-based material…"

As noted in the response to comments 9 and 10, the TGA measurements were used to estimate the maximum CO2 capacity attainable during MgO carbonation, and to verify that the equilibrium temperature of the reaction was in reasonably good agreement with the estimated values from the thermodynamic database used (FactPS). Hence, as the modelled reaction was assumed to be pseudo-isothermal for each carbonation or calcination step, the temperature ramp rates used in experiments were not incorporated into the modelled system.

15. In Figure S2, what occurs if the system experiences unresolved surplus or deficit conditions? Also, where is the gas turbine integrated, as mentioned in Figure 1?

To clarify this point, we have adjusted Figure S2 (see below) to show the calculation of net wind power curtailment in the event of unresolved daily surplus, and the calculation of net gas turbine usage (with associated CO2 emissions) in the event of an unresolved daily deficit. We have also added the following note in the neighbouring text:

"In the event of a net deficit in energy supplied from the windfarm and from stored H2, we calculated the required energy imports and CO2 emissions from natural gas turbine usage. Similarly, in the event of a net surplus in energy generated from the windfarm that exceeded available energy storage capacity, the excess energy generation was curtailed."

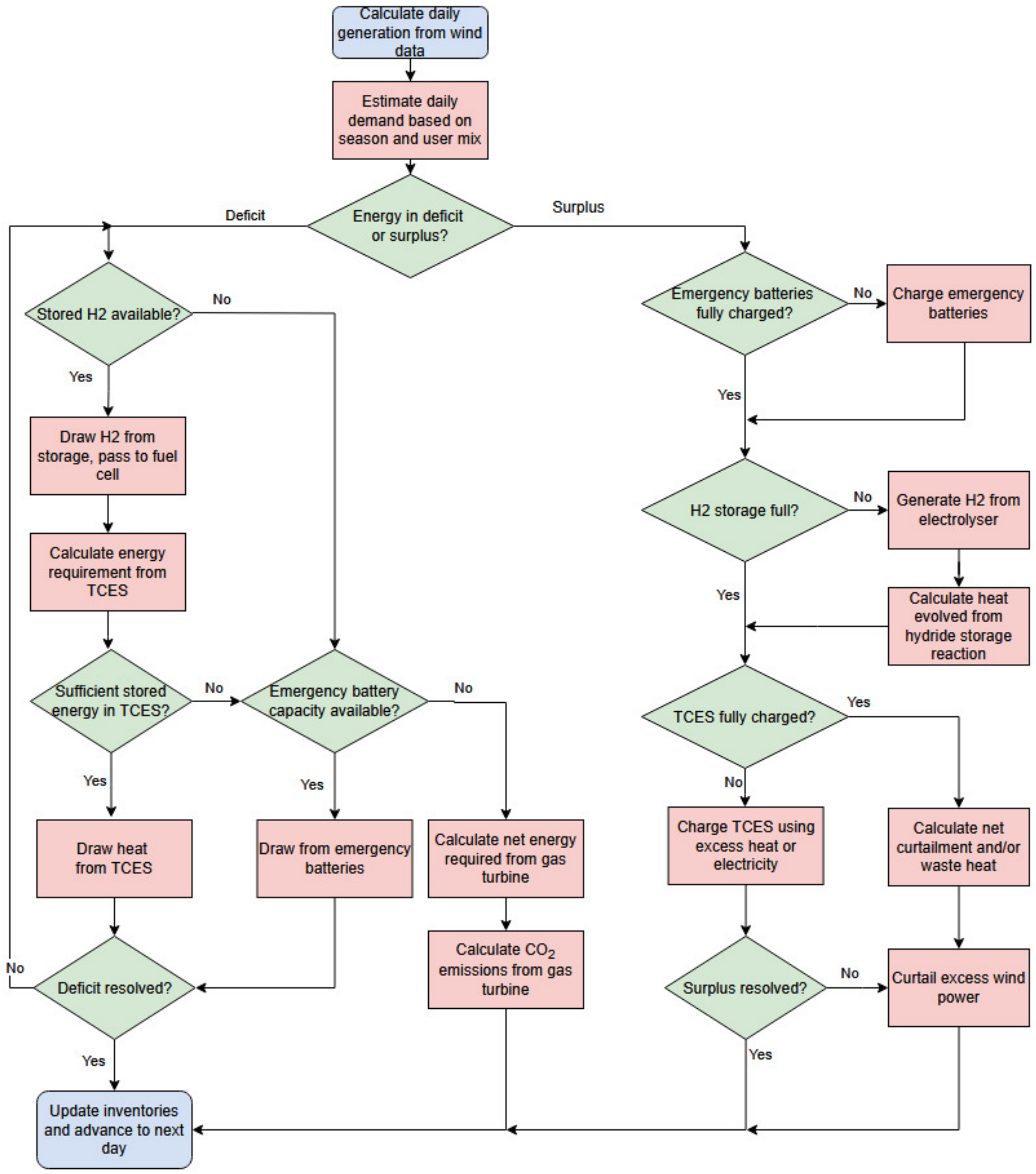

16. In Figure S3, what are the pressure ratios of the multi-stage compressors for the flue gas and CO2 streams? Given that a typical air compressor has a pressure ratio of about 5, one stage might be sufficient for the flue gas, but four to five stages could be required for CO2 compression.

In response to this query, we have added the following note to the 'Compressors and turbo-expanders' section of the SI:

"Under base case assumptions (ηisentropic = 0.85, T2 = 473 K), for flue gas compression from ambient pressure to 4 bara in CMP1a and CMP1b, pressure ratios of up to 4 were used in each compressor in series. For CO2 compression from the reactor R2 outlet to pipeline injection at 150 bara in CMP2a, CMP2b, and

CMP2c, overall pressure ratios of 5.8 were used for each of the three compressors in series, corresponding to centrifugal compressors with multiple impellers and sequential compression stages per unit [68].”

17. The system includes numerous heat exchangers (e.g., HX1, HX4, HX6, HX7). Is there a heat exchange network design underlying these connections? For instance, what is the rationale for including both HX6 and HX7, and how were the interconnections determined?

We have added the following paragraph to the supplementary information (Section S4.5) to clarify the purpose of each exchanger in the network during hydrogen storage and release.

“Heat exchangers HX1, HX4, HX6, and HX7 were connected in a heat transfer loop to transfer heat between unit operations using the heat transfer oil. During hydrogen storage, the hydrogen leaving the fuel cell at c. 50◦C is heated to 352.5◦C in HX1, with heat supplied from the heat transfer loop, before being fed to reactor R1. In reactor R1, the heat evolved from the exothermic hydrogenation reaction is transferred to the heat transfer loop using HXR1. Heat is then transferred from the heat transfer loop to drive the endothermic calcination reaction in reactor R2, via the heat transfer coil HXR2. In the event of a net surplus of heat evolved from the hydrogenation reaction relative to the heat used to pre-heat the hydrogen feed and drive calcination, any excess heat is removed in cooler HX7. Then, the pure CO2 released from R2 in the calcination reaction is intercooled in HX8a-8d in between compression stages prior to pipeline injection at 150 bara. During hydrogen release, heat is supplied to drive the endothermic dehydrogenation reaction from the heat transfer loop in HXR1. The flue gas feed to R2 is intercooled between compression stages in HX2a and HX2b. Then, the feed is pre-heated in HX3 and HX4, with HX3 transferring heat from the hot hydrogen outlet at 347◦C leaving R1, and HX4 supplying heat from the heat transfer loop. The reaction heat from the exothermic carbonation reaction in R2 is transferred to the heat transfer loop in HXR2. The hydrogen leaving HX3 is further cooled to 80◦C in HX5 before being transferred to the fuel cell, with no heat recovered from the outlet waste water. In the event of a net deficit in heat generated from the carbonation reaction relative to the heat required to pre-heat the reactor feed and drive the dehydrogenation reaction, additional supplementary heat is supplied to the heat transfer loop using electric heater HX6.”

We have also added a brief ‘purpose’ column to Table S4 in the supplementary information.

Code	Purpose	Hot Fluid	Cold Fluid	Heat transfer coefficient (kW $m^{-2}K^{-1}$)
HX1	Pre-heater for feed to hydrogen storage reactor	Heat transfer oil	Hydrogen	0.1
HX2a-b	Intercooling between R2 feed compression stages	Flue gas	Cooling water	0.2
HX3	Heat integration between hydrogen outlet and carbonation reactor feed	Hydrogen	Flue gas	0.05
HX4	Additional pre-heating for carbonation reactor feed	Heat transfer oil	Flue gas	0.1
HX5	Cooling hydrogen feed to fuel cell	Hydrogen	Cooling water	0.1
HX6	Supplementary electrical heating to resolve net reaction heat deficits	N/a (Electric heater)	Heat transfer oil	N/a
HX7	Supplementary cooling water to resolve net reaction heat surpluses	Heat transfer oil	Cooling water	0.15
HX8a-d	Intercooling between CO_2 compression stages	CO_2	Cooling water	0.2
R1	Heat transfer coil in R1 to supply/remove heat from (de)hydrogenation reaction	Heat transfer oil*	Hydrogen*	0.1
R2	Heat transfer coil in R2 to supply/remove heat from (de)carbonation reaction	CO_2*	Heat transfer oil*	0.1

18. For reactors R1 and R2, if both are maintained near 350 °C, additional thermal losses and reduced adsorption capacity should be expected. What is the rationale for selecting this temperature level? Under such conditions, neither system may achieve optimal adsorption performance.

To clarify this point, we have added the following note to the SI, Section S4.1:

"For the TCES system to operate, there must be a temperature driving force for heat transfer from the TCES reactor to or from the hydrogen storage reactor, and hence, the exothermic step (hydrogen storage or TCES carbonation) must operate at a higher temperature than the endothermic step (hydrogen discharging or TCES calcination). Over the thermodynamic overlap range shown in Fig 1 (335-415◦C) where both reactions are feasible, selection of temperature, and temperature difference between the reactors, is arbitrary, and for any selected temperature, will not be optimal for either reaction. However, the efficiency of hydrogen storage and release, and the resulting round-trip efficiency, depend on both the energy required to pre-heat the H2 and CO2 feeds and reactor temperature, and the compression duties as shown in Fig. S4. Therefore, altering the assumed reactor operating temperature, temperature difference between reactors, CO2 feed fraction, and per -pass CO2 conversion, affect the overall round trip efficiency, shown in Fig. S3, with optimal overall efficiency at a nominal operating temperature of 350◦C, with a temperature difference of c.5◦C between reactors."

with a further explanation of the effect of raising or lowering reactor temperature given in the subsequent section (S4.1).

The following addition was also made to the main manuscript, at the start of the **"System Design and Round-Trip Efficiency"** section:

"As shown in the SI, Fig. S3, a reactor operating temperature of 350◦C provided the optimal trade-off in thermodynamic efficiency for both reactions, giving the highest overall round-trip efficiency of conditions investigated."

19. Did you assume that R1 absorbs 100% of the hydrogen produced by the electrolyzer? Please justify whether this assumption is realistic.
In response to this comment and comment #7, we have added the following note to the Supplementary Information, Section S4.3:

"Hydrogen absorption was assumed to be limited by the equilibrium partial pressure of the hydride formation reaction and the experimentally-derived hydrogen capacity of the Mg-based materials, assuming that over the 24 hour modelling period, the system was able to approach thermodynamic equilibrium [39], with some gaseous hydrogen remaining in the reactor headspace in order to maintain equilibrium. Hence, the round-trip efficiency parameters estimated here represent an upper-bound estimate for the case where thermodynamic equilibrium is achieved; if excess hydrogen generation were required to compensate for incomplete absorption, the effective round-trip efficiency would be decreased. In practice, to achieve an additional driving force for absorption, the hydrogenation reaction could be operated above equilibrium pressure by using the electrolyser output directly (i.e. removing pressure regulation valve TV1)."

20. What efficiencies were assumed for the electrolyzer and fuel cell? Please also provide a reference supporting the assumed six-year lifetime. Some numbers are missing in the SI.
21. In Section S4, several numerical values are presented without references, and some assumptions appear deterministic. Please provide citations for all key parameters and clarify the basis for the assumed values.

22. The reference year should be included in Table S5, since inflation affects the LCOE calculations.
23. The parameters in Table S5 appear to be sourced from different references. How were data uncertainties treated? How did you define the base, optimistic, and pessimistic scenarios? The lifetimes and degradation or make-up rates of the hydrogen storage alloy and magnesium carbonate should also be considered.

In response to comments 20-23, we have reorganised the source data used as an input for the cost-estimation model. Cost parameters for key items of process equipment (*e.g.* windfarm, fuel cell, electrolyser), are presented in the main manuscript, Table 1 (with reference year for each item). Other values used from literature are presented, with reference years, in the SI, Tables S5 and S16.

Table 1 Parameters used for estimation of capital and operating costs for each scenario, with corresponding literature source(s). Reference years correspond to the range of publication dates of the cited literature sources.

Item	Unit	Base	Pess.	Opt.	Ref(s)	Ref. years(s)
Fuel cell capital cost	$\$ \text{ kW}^{-1}_{installed}$	2687	4000	1620	[81–83]	2015-2025
Electrolyser capital cost	$\$ \text{ kW}^{-1}_{installed}$	931	2000	400	[84–89]	2022-2024
Fuel cell/electrolyser lifetimes	years	8.5	4.5	14	[90–95]	2015-2024
Battery capital cost	$\$ \text{ kWh}^{-1}_{capacity}$	281	405	134	[17, 96–100]	2021-2023
Onshore wind capital cost	$\$ \text{ kW}^{-1}_{nameplate}$	1680	2800	1050	[101–106]	2021-2024
Offshore wind capital cost	$\$ \text{ kW}^{-1}_{nameplate}$	3785	6500	1839	[102, 105–110]	2020-2025
Hydrogen storage alloy cost	$\$ \text{ kg}^{-1}$	16	8	60	[17, 111]	2024
Hydrogen storage alloy lifetime	y	16	8	25	[4]	2023

With the following explanatory text added to the accompanying paragraph:
"Capital and operating costs were estimated using reported values from literature (shown in Table 1, with additional parameters given in the SI, Tables S5 and S16), using the mean of recent reported values as the base case estimate, and maximum and minimum reported values for the optimistic and pessimistic cases as appropriate (i.e. maximum lifetime, minimum cost for the optimistic case and vice versa for the pessimistic case). For input parameters with limited available data, or common items of process equipment (e.g. turbomachinery), approximate cost estimation correlations from literature were used [96] where available, adjusted for inflation using the Chemical Engineering Plant Cost Index (CEPCI) [73]. To quantify the uncertainty in estimated system parameters, Monte Carlo simulation was performed by randomly sampling input parameters over the reported range, as described in the SI, Section S7.1."

Given difficulties in directly estimating relative inflation of different items of process equipment, cost data from sources older than 2019 (selected as an arbitrary cutoff year) were adjusted to $_2019 using CEPCI, while noting that differential rates of inflation or cost-decreases (e.g. as a result of improved efficiency in windfarm manufacturing) between items of equipment introduces significant uncertainty into the final estimated values of CAPEX, OPEX, and LCOE, reflected in the estimated uncertainty ranges.

Regarding the efficiency of the electrolyser and fuel cell, we have added the following explanation next to Figure 3:

"The main energy conversions when electricity is converted to hydrogen, and back to electricity when needed, are shown in the Sankey diagram in Fig. 3, estimated using

base-case modelling assumptions (summarised in the SI, Section S4, Figs. S4 and S3, and Section S5, Table S5), with assumed PEM electrolyser and fuel cell efficiencies of 0.019 kgH2 kWh−1 and 12.3 kWh kg−1 H2 (37% LHVH2) respectively, based on reported values from literature [52, 74, 77]. As shown in the SI, Fig. S3, a reactor operating temperature of 350◦C provided the optimal trade-off in thermodynamic efficiency for both reactions, giving the highest overall round-trip efficiency of conditions investigated. The reactors were assumed to be insulated and nominally isothermal, with additional heat supplied to compensate for ambient heat losses by electrical heating as shown in the process flow diagram, Fig. 9."

Regarding the degradation and make-up rates of the solid materials, we have added the following note to the supplementary information, Section S4.1:

"The Mg-based alloy was assumed to have an approximate lifetime of 3,000 H2 absorption-desorption cycles in the base-case scenario based on reported values from literature, corresponding to around 16 years of operation [4]. The cost of material makeup was therefore estimated at 1/16 of capital cost per year, not including any scrap value or recycling costs, corresponding to around 14% of operating expenditure as shown in Fig. 7. Given the relatively low cost of the MgO-based CO2 sorbents used for TCES (<1% total capital expenditure for all modelled cases), degradation over time was not modelled explicitly. However, some MgO-based sorbents are vulnerable to sintering, resulting in a loss of CO2 capacity over repeated carbonation and calcination cycles [38, 52]. Therefore, for practical operation, the MgO-based sorbent would likely need to be replaced or regenerated periodically, in order to maintain system performance, but this was not expected to have a significant effect on total operating expenditure."

Reviewer #4 (Remarks to the Author):

The authors present a techno-economic study of a coupled system that combines Mg-based solid hydrogen storage with a magnesium-looping thermo-chemical energy storage / carbon-capture cycle ($MgO \leftrightarrow MgCO_3$). The core innovation is the use of waste heat from the solid-state hydrogen storage reaction to drive Magnesium looping as a secondary process. This leads to a complex interconnected system for energy storage and carbon removal.

The study presents the technoeconomic assessments of a variety of technical scenarios based on a model of the energy flow.

The work supports the claims. No additional evidence needed.

There seems to be no flaws in the data analysis. The methods are sound and described in detail. Computational routines and data is made available.

No revision is needed. Maybe the authors could consider these remarks and possibly add some clarifictions:

A turbomachinery (compression/expansion) path is coupled with an electrolyser/fuel cell path. This might lead to challenges in the engineering implementation. Design, scale up, longterm attrition of the reactors will also lead to engineering challenges.

We thank the reviewer for this comment. While the primary purpose of this study was to establish the overall operation of the system in terms of thermodynamic efficiency and approximate cost-effectiveness, we recognise that practical design and implementation of our proposed system would present significant challenges meriting future study, in particular, in the design of a control system able to cope with the different characteristic rates associated with each process. We have added the following comment to the discussion, noting these issues:

"Additionally, the system couples two relatively slow chemical reactions (hydrogen sorption and MgO carbonation) with turbomachinery for gas compression, each with different characteristic start-up and shut-down times, and with lag from turbomachinery introducing significant difficulties in process control, especially during the transition periods between carbon capture and CO2 release. While directly modelling the relative rates of each process was beyond the scope of this study, future work should consider the design of control systems able to allow the system to cope with varying load over time, incorporation of additional short-term electricity storage in the form of batteries, and short-term compressed gas storage using buffer tanks."

Costs associated with reactor/material attrition are not modelled explicitly, but are assumed to comprise a portion of the material replacement costs (estimated for the Mg-based hydrogen storage material and the MgO-based CO2 sorbent), and the fraction of OPEX assigned to general maintenance (as a fraction of CAPEX *per* year of operation).

Is everything needed available on site, e.g. flue gas and if necessary energy (i.e. for Dunkelflaute) ? What is the scenario/ use case of the proposed system: As an add on to power stations using the power from the grid, as an add on to windparks as long term energy storage ? Is the proposed system rather an enhanced hydrogen energy storage or rather an enhanced carbon capture from flue gas ?

How many such systems /what capacity could be deployed i.e. in the UK and what would be the expected benefit ?

The system we propose here acts primarily as long-term energy storage, as a complement to new or existing wind infrastructure, such that variable windfarm

output can be converted into continuous electricity supply to consumers. However, the system requires access to flue gas in order to capture CO2 and offset the emissions associated with the use of backup gas turbine power generation during sustained periods of limited wind (as shown in Figs. 4 and 6). Therefore, the system needs to be located with a source of industrial CO2 emissions. Such sites in the UK could be at one of the seven industrial clusters identified by the UK government (DESNZ, 2025; Sovacool *et al.*, 2022) as suitable for carbon capture projects (responsible for ~50% of total UK industrial CO2 emissions); for instance, at the Teesside industrial cluster, offshore wind power, hydrogen production, CCGT power generation and carbon capture are already in the process of being co-located alongside extant industrial activity (Herman *et al.*, 2025).

Another consideration in siting is the construction of sufficient long-term storage capacity for compressed hydrogen, with Fig. 5 showing how the overall cost of electricity is dependent on the ratio of rated windfarm output to storage capacity.

To clarify these points regarding siting, we have added the following point to the end of the 'Challenges and Future Outlook' section

"The system we describe in this work comprises a combined system for converting the intermittent power output from a windfarm into a continuous power supply to consumers, using hydrogen storage to manage short term fluctuation. Assuming relatively efficient electricity transmission via the power grid, the long term energy storage would not need to be co-located with the windfarm, provided that storage capacity and backup gas turbine capacity was allocated in an appropriate ratio to windfarm output. However, in order to have access to sufficient flue gas, the hydrogen storage system does need to be co-located with an otherwise-unabated CO2 point source (e.g. a steel or cement plant). Such suitable sites in the UK could be at one of the industrial clusters identified by the UK government as suitable for carbon capture projects, which are responsible for c. 50% of non-power related UK industrial CO2 emissions (c. 36.1 Mt y−1) [63–65]"

Comparison of costs in Figure 8. Maybe include an uncertainty margin for the estimates (especially for the new technologies) unsicherheitsspanne

In response to this and previous comments, we have substantially altered the estimation method used for the values in Figure 8. Rather than using point estimates for three representative cases, we performed Monte Carlo simulation sampling over the input parameter range (described above) to estimate the distribution of overall costs for each case. The revised version of Figures 8b and 8c, showing cost ranges represented as box-violin plots, is shown below.

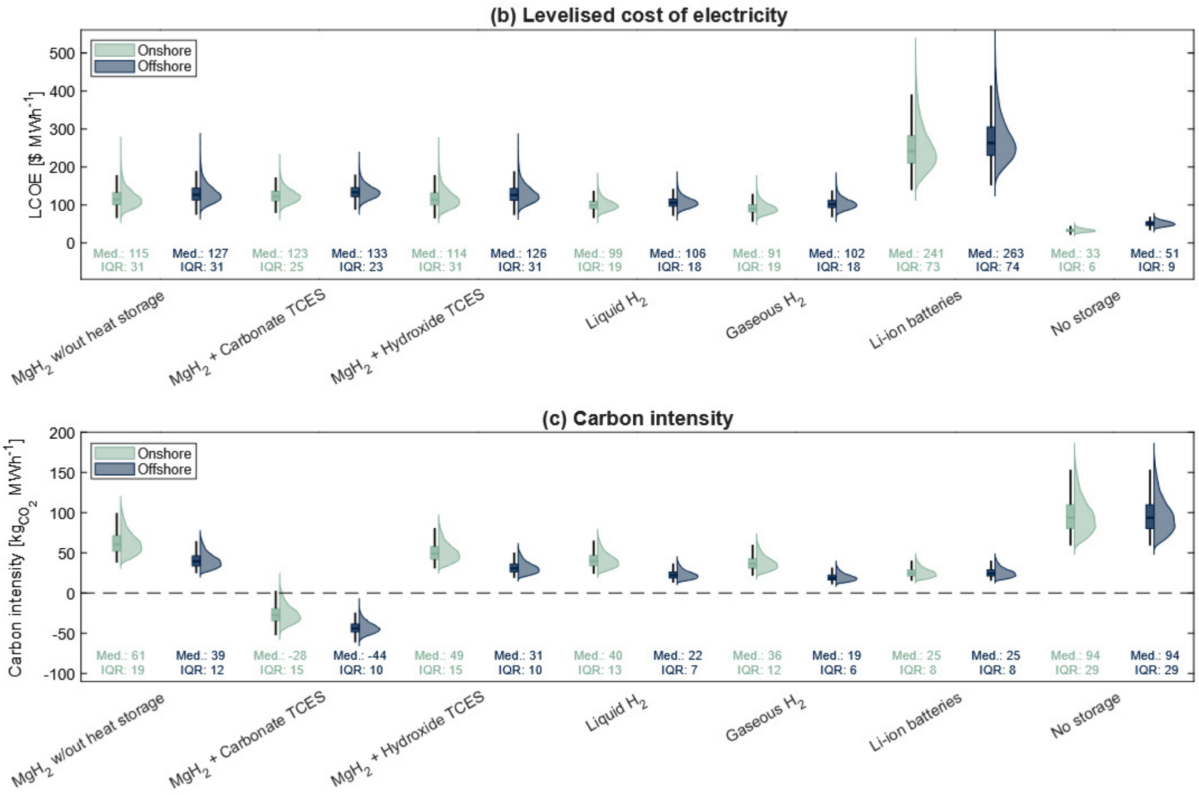

Caption Figure 2: Define PCT and TGA

We have adjusted the caption as follows:

"Figure 2 (a) Hydrogen absorption pressure-composition-temperature (PCT) isotherms for the Mg-based hydrogen storage alloy prepared here, (b) fitted equilibrium curve of hydrogen partial pressure against temperature, as compared with values reported by Lin et al. [41] for an Mg-based alloy material, and values calculated for Mg + H2 ⇌ MgH2 using values from the FactPS [42] and NASA Glenn coefficient thermodynamic databases [43] (with the latter only providing data for MgH2 up to 327◦C), (c) ten cycles of hydrogenation (pH2 = 20 bara) and dehydrogenation in He at 350◦C for the Mg-based material prepared, showing complete conversion during each 30 min step, (d) three carbonation-calcination cycles for MgO-based material in the thermogravimetric analyser (TGA), normalised with respect to the final mass. Irreversible mass loss in the first cycle corresponds to removal of adsorbed impurities from exposure to laboratory air."

Figure 7. Numbers in images are barely readable

As indicated in responses to previous comments, we have re-drawn Figure 7 to reflect the range of uncertainty in total costs (CAPEX, OPEX, LCOE), represented as box-violin plots, and the breakdown of averaged CAPEX and OPEX costs for

onshore and offshore cases. The revised figure, with larger numbers, is shown below:

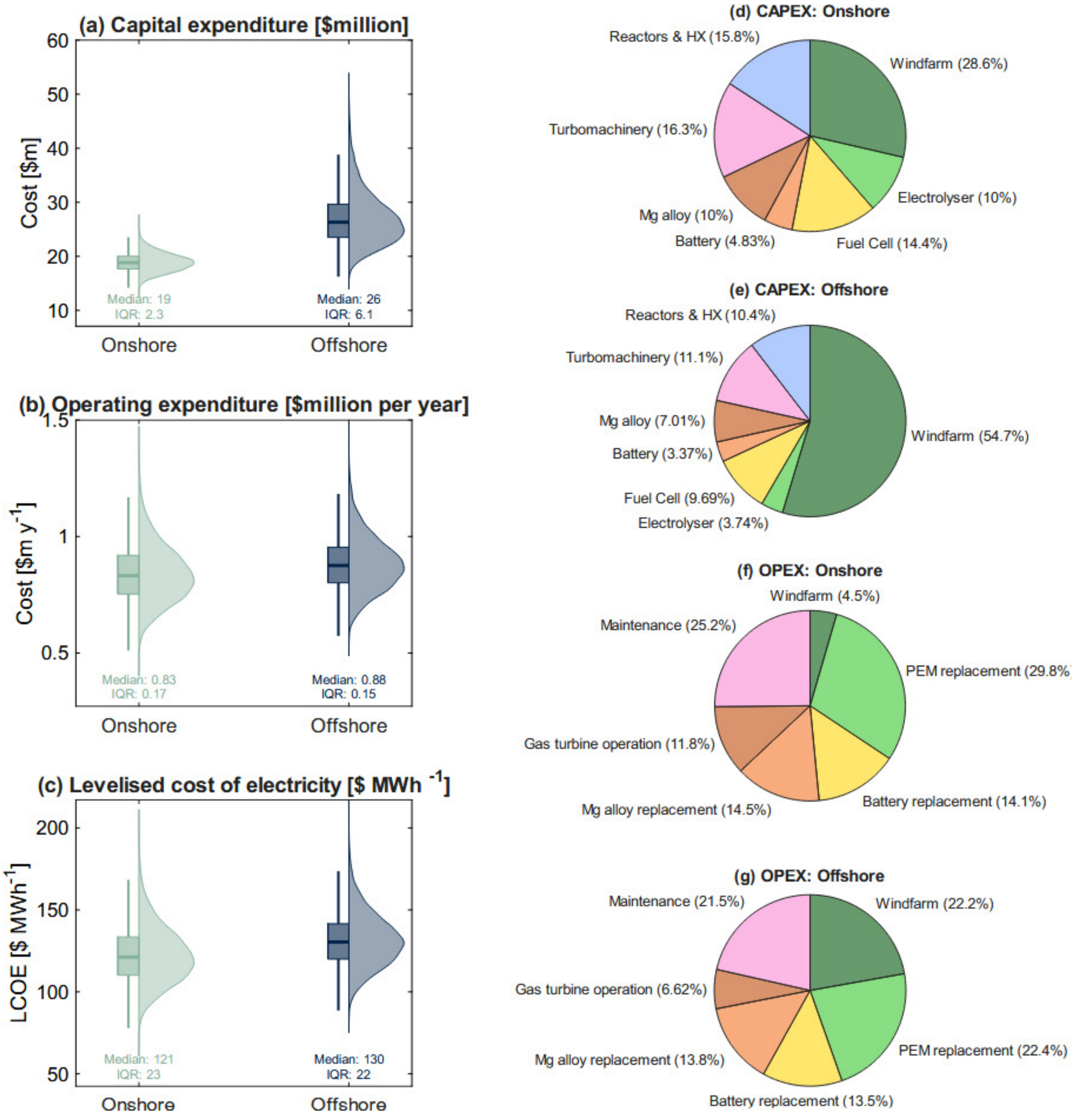

Additional works cited in responses to reviewer comments:

de Rango, P., Marty, P. & Fruchart, D. Hydrogen storage systems based on magnesium hydride: from laboratory tests to fuel cell integration. *Applied Physics A* **122**, 126 (2016). https://doi.org/10.1007/s00339-016-9646-1

Department for Energy Security and Net Zero. UK carbon capture, usage and storage (CCUS) (2025). URL https://www.gov.uk/government/collections/uk-carbon-capture-usage-and-storage-ccus

Herman, K. S., Iskandarova, M., & Sovacool, B. K. Imagining a net-zero Teesside: actors, networks, and expectations in industrial decarbonisation megaprojects. *Environmental Research Communications*, **7**(1), 015007 (2025). https://doi.org/10.1088/2515-7620/ad8f99

Sovacool, B. K., Geels, F. W. & Iskandarova, M. Industrial clusters for deep decarbonization. *Science* **378** (6620), 601–604 (2022). https://doi.org/10.1126/science.add0402

Wang, X. et al. A techno-economic study of photovoltaic-solid oxide electrolysis cell coupled magnesium hydride-based hydrogen storage and transportation toward large-scale applications of green hydrogen. *Energy & Environmental Science* **17**, 8429–8456 (2024). https://doi.org/10.1039/D4EE04224G .

Responses to reviewers (Revision 2)

To the anonymous peer reviewers,
We thank you again for your assessment of our manuscript, and your suggestions for improvement. Please find below our responses to your additional comments on the 2nd revised version of the manuscript.

Yours sincerely,
Binjian Nie and Alexander Harrison

Reviewer #1 (Remarks to the Author):

The revised manuscript has addressed my comments and its quality has been improved.

We thank the reviewer for this assessment and are glad that our modifications were sufficient.

Reviewer #2 (Remarks to the Author):

Thank you for your detailed response. The questions have been thoroughly addressed, and the manuscript has significantly improved in clarity.
I have only one minor comment: Figure 9 could be moved to the Supporting Information (SI) because it is less visually compelling than the other figures. The overall concept is already clearly illustrated in Figure 1. In this context, the detailed process design shown in Figure 9 appears less essential to the main narrative.

In response to this comment, we have removed Figure 9 from the main manuscript, and moved the figure back to the Supporting Information, Section 4.1 (now designated Figure S3).

Regarding the Graphical Abstract, I suggest refining the right-hand side. Instead of presenting three separate plots, it may be more effective to explicitly highlight the key advantages of integrating solid hydrogen storage with the carbon capture loop, as these represent the core insights conveyed by those three figures. Emphasizing this integration would make the central contribution clearer and more impactful.

We thank the reviewer for their suggestion. However, we have been requested by the editorial team to remove the graphical abstract altogether, in line with journal policies, and so have removed the figure from the manuscript.

Reviewer #3 (Remarks to the Author):

I co-reviewed this manuscript with one of the reviewers who provided the listed reports. This is part of the Nature Communications initiative to facilitate training in peer review and to provide appropriate recognition for Early Career Researchers who co-review manuscripts.

Reviewer #4 (Remarks to the Author):

All issues of initial review are addressesd.

Reviewer #4 (Remarks on code availability):

Lack of time.

We thank reviewers 3 and 4 for their assessment of our manuscript and their helpful comments, and are glad that any concerns were adequately addressed.
